# Supplementary material for: Violence, self-harm and drug or alcohol misuse in adolescents admitted to hospitals in England for injury: a retrospective cohort study
Source: BMJ Open. 2015 Feb 7;5(2):e006079. doi: 10.1136/bmjopen-2014-006079 (PMC4322205; doi:10.1136/bmjopen-2014-006079)
Supplement: Author's manuscript [file bmjopen-2014-006079.draft_revisions.pdf]

BMJ Open

Victimisation, self-harm and drug or alcohol misuse in adolescents admitted to hospitals in England for injury: a retrospective cohort study

Journal:	*BMJ Open*
Manuscript ID:	bmjopen-2014-006079
Article Type:	Research
Date Submitted by the Author:	10-Jul-2014
Complete List of Authors:	Herbert, Anne; UCL Institute of Child Health, Population, Policy and Practice Programme<br>Gilbert, Ruth; UCL Institute of Child Health, Population, Policy and Practice Programme<br>Gonzalez-Izquierdo, Arturo; UCL Institute of Child Health, Population, Policy and Practice Programme; UCL Institute of Child Health, Centre for Paediatric Epidemiology and Biostatistics London<br>Li, Leah; UCL Institute of Child Health, Population, Policy and Practice Programme
**Primary Subject Heading**:	Paediatrics
Secondary Subject Heading:	Mental health, Health services research
Keywords:	MENTAL HEALTH, Non-accidental injury < PAEDIATRICS, Substance misuse < PSYCHIATRY, Suicide & self-harm < PSYCHIATRY

SCHOLARONE™
Manuscripts

For peer review only

Victimisation, self-harm and drug or alcohol misuse in adolescents admitted to hospitals in England for injury: a retrospective cohort study

Annie Herbert, Centre for Paediatric Epidemiology & Biostatistics, University College London Institute of Child Health, 30 Guilford Street, London, WC1N 1EH, Tel: +44207 905 2790, Fax: +44207 905 2793, annie.herbert.12@ucl.ac.uk

Annie Herbert, M.Sc.[1], Ruth Gilbert, M.D.[1], Arturo González-Izquierdo, Ph.D.[1], Leah Li, Ph.D.[1]

1. Centre for Paediatric Epidemiology & Biostatistics, University College London Institute of Child Health, 30 Guilford Street, London, WC1N 1EH, UK

Short title: Adversity in adolescents admitted to hospital for injury

Key words: Wounds and Injuries, Violence, Self-injurious Behaviour, Substance-related Disorders, Adolescent

Word count: 2,509

No. figures: 2

No tables: 2

No references: 32

No supplementary files for online only publication: 5

For peer review only - http://bmjopen.bmj.com/site/about/guidelines.xhtml

ABSTRACT

Objectives

Of adolescents in the general population in England, we aimed to determine 1) the proportion that has an emergency admission to hospital for injury related to adversity (victimisation, self-harm or drug or alcohol misuse) and 2) the risk of recurrent emergency admissions for injury in adolescents admitted with adversity-related injury compared with those admitted with accident-related injury only.

Design

We used hospital administrative data (Hospital Episode Statistics) to identify 10-19 year olds with emergency admissions for injury in England in 1998-2011. We used Office for National Statistics mid-year estimates for population denominators.

Results

Approximately 4.3% of adolescents in the general population had one or more emergency admissions for adversity-related injury (140 512/3 254 046; girls 4.6%, boys 4.1%), accounting for 59.9% of all emergency admissions for injury in girls and 29.6% in boys. Admissions for self-harm or drug or alcohol misuse commonly occurred in the same girls and boys. Recurrent emergency admissions for injury were more common in adolescents with adversity-related injury (girls 17%; boys 17%) than in those with accident-related injury only (girls 5%; boys 7%), particularly for adolescents with adversity-related injury related to multiple types of adversity (girls 21%; boys 24%).

Conclusion

For peer review only

Hospital-based interventions should be developed to reduce the risk of future injury in adolescents admitted for adversity-related injury.

For peer review only

For peer review only - http://bmjopen.bmj.com/site/about/guidelines.xhtml

What is already known on this subject

- Adolescents exposed to adversity (victimisation, self-harm or drug or alcohol misuse) often use health services, and repetitively.
- Many adolescents in the general population are exposed to multiple types of adversity during their adolescence.

What this study adds

- One in 20 adolescents in England have an emergency admission to hospital for adversity-related injury between 10 and 19 years old.
- Many adolescents admitted with adversity-related injury (particularly girls) are admitted with multiple types of adversity, self-harm and drug or alcohol misuse being the most common combination.
- Readmission is more common in these adolescents than those admitted with accident-related injury only.

Strengths and limitations of this study

- Hospital Episodes Statistics (HES) captured data on all admissions to National Health Service hospitals in England at 10-19 years old in this study's cohort.
- The longitudinal link between admissions for each individual in HES data allowed us to study the burden of multiple emergency admissions for injury over time.
- However, victimisation, self-harm and drug or alcohol misuse are not always recognised at an admission, or consistently recorded, and

For peer review only

For peer review only - http://bmjopen.bmj.com/site/about/guidelines.xhtml

therefore this study's estimates of prevalence of adversity are likely to be underestimates.

For peer review only

For peer review only - http://bmjopen.bmj.com/site/about/guidelines.xhtml

INTRODUCTION

Many adolescents exposed to adversity such as victimisation (maltreatment or assault), self-harm, or drug or alcohol misuse use secondary health services,[1 2] often repetitively.[3 4] For example, in a self-report survey of 15-16 year olds in England, 12.6% of those who had self-harmed had presented to hospital.[2] It is also estimated that approximately one-third of patients attending a hospital in England for self-harm re-attend for self-harm in the following year.[4] Improved management of adolescents exposed to adversity could reduce risk of repetition as well as the burden on secondary care.[5-7]

An admission to hospital provides the 'teachable moment',[8] where both adolescents and their families may be more likely to engage an intervention than if they had received it elsewhere. Hospital-based interventions to reduce the risk of future harm could benefit these adolescents by reducing episodes of injury, and may reduce recurrent emergency (i.e., acute or unplanned) admissions for injury.

To-date, there is a lack of evidence on how different types of adversity related injury occur in the same adolescents, over time. In addition, policy makers and service providers need know how many adolescents have an emergency admission to hospital for adversity-related injury, their characteristics and their specific rates of re-admission if they are to be feasibly targeted for intervention.

In this study we used administrative hospital data and Office for National Statistics (ONS) mid-year population estimates to estimate the number of adolescents in the general population who ever have an

For peer review only - http://bmjopen.bmj.com/site/about/guidelines.xhtml

For peer review only

emergency admission to hospital for injury. We then estimated the prevalence of emergency admissions for injury related to victimisation, self-harm and drug or alcohol misuse (alone and co-occurring) in the general population. Finally we determined the risk of recurrent emergency admissions for injury in adolescents who had at least one admission between 10 and 19 years for adversity-related injury compared with adolescents only ever admitted for accidental injury during the same period.

For peer review only

METHODS

Study population

Using administrative data from all admissions to National Health Service hospitals in England (Hospital Episode Statistics [HES]) in 1998-2011,[9] we derived a retrospective cohort of adolescents who turned 10 years old in 1998-2002, who could be observed throughout adolescence until 19 years old (Supplementary Table 1).[10] Each individual also had to have at least one emergency admission for injury between 10 and 19 years old.

Admission data

The Health and Social Care Information Centre provided pseudonymised data on hospital admissions, use of which did not require Research Ethics Committee approval.[11] We analysed any hospital transfers or admissions within one day after discharge as the same admission, as previously described.[12] We used the variable for method of admission ('admimeth') to define an emergency admission. We used all International Classification of Diseases-10 (ICD-10) diagnosis codes recorded during an admission to categorise admissions as being for injury related to adversity, an accident or other causes (Supplementary Table 2).[13]

Types of injury and age at emergency admission

We defined an emergency admission for injury as being for related to adversity, comprising victimisation (maltreatment/assault), self-harm or drug or alcohol misuse, using mutually exclusive clusters of ICD-10 codes (Supplementary Table 2). Victimisation was defined by previously validated

For peer review only - http://bmjopen.bmj.com/site/about/guidelines.xhtml

codes, which would trigger consideration of maltreatment by carers or of assault.[12 14 15] We defined self-harm using codes that mentioned either 'self-harm' or 'self-poisoning', and excluded 'undetermined intent'. Drug or alcohol misuse was defined by codes that mentioned 'alcohol', 'drugs', 'noxious substance' or 'solvent'. We considered alcohol use (Z72.1) to be misuse only in adolescents younger than 18. We defined an injury as being related to accidents only if no adversity codes were present but there were codes from the ICD-10 *Accidents* subchapter (V01-X59).[13]

We grouped age at each admission as 10-14 years, 15-17 years and 18-19 years, to reflect age of onset of puberty (10-14 years), age of sitting secondary school exams (15-18 years) and the legal age for buying alcohol (18 years).[16-19]

Classification of adolescents according to types of injury and age at emergency admissions

We classed adolescents into groups according to all of their emergency admissions for injury between 10 and 19 years old. Adolescents were classed as belonging to the 'AdvRI' group (any adversity-related injury between 10 and 19 years old), 'AccRI Only' group (no adversity-related injury but one or more accident-related injuries) or 'Other Causes' group (no adversity-related or accident-related injuries) (Supplementary Figure 1). Among adolescents in the AdvRI group, we determined the proportion exposed to victimisation-, self-harm- and drug or alcohol misuse-related injury at age 10-19, respectively. We further classified the AdvRI group into seven mutually exclusive sub-groups: Victimisation Only, Self-harm Only, Drug or Alcohol Misuse Only (DA Only), Victimisation and Self-harm (V+SH),

For peer review only - http://bmjopen.bmj.com/site/about/guidelines.xhtml

Victimisation and Drug or Alcohol Misuse (V+DA), Self-harm and Drug or Alcohol Misuse (SH+ DA) and Victimisation, Self-harm and Drug or Alcohol Misuse (V+SH+DA).

We also grouped adolescents as above, according to their emergency admissions for injury at 10-14 years only, 15-17 years only and 18-19 years only (Supplementary Figure 2).

Defining an adolescent as having a chronic condition

We used a previously validated cluster of ICD-10 codes to identify adolescents who had been affected by a chronic condition in any admission between 10 and 19 years old (Supplementary Table 2).

Population denominators

We used ONS mid-year population estimates to derive population denominators.[20 21] These data are freely available online broken down by sex and year of age.

Analyses

We estimated the proportion of adolescents in the general population who had an emergency admission for injury between 10 and 19 years old, by using the number of adolescents in our derived retrospective cohort as the numerator and ONS mid-year estimates for 10 year olds in 1998-2002 as the population denominator. We then calculated these proportions by types of injury at 10-19 years old (adversity-related [AdvRI group and seven mutually exclusive subgroups] and accidents only [AccRI Only group]) and by age group, as described above.

For peer review only - http://bmjopen.bmj.com/site/about/guidelines.xhtml

We calculated the proportion of adolescents in the AdvRI group (and subgroups) and in the AccRI Only group, who had an emergency admission for injury twice and three or more times between 10 and 19 years old. We also calculated the proportions of adolescents with two or three or more admissions of any type (including non-emergency and non-injury).

We reported all results separately for girls and boys, since differences between girls and boys have been reported for prevalence of adversity in the general population.[1 22-24] We calculated 95% confidence intervals for all proportions but did not present them as they were all too narrow to convey any useful information (within one unit of the sample estimate). Analyses were carried out in StataSE 12.

For peer review only

For peer review only - http://bmjopen.bmj.com/site/about/guidelines.xhtml

RESULTS

There were 1 033 702 adolescents in HES admissions data in 1998-2011, of which 402 916 formed the study cohort (462 476 emergency admission for injury, 802 682 admissions of any type [including non-emergency and non-injury]) (Table 1), representing 12.4% (402 916/3 254 046) of the adolescent population. Twice as many boys as girls had an emergency admission for injury during adolescence (144 158/1 588 942 girls in the population [8.7%]; 258 503/1 665 104 boys [16.3%]).

For peer review only - http://bmjopen.bmj.com/site/about/guidelines.xhtml

For peer review only

Table 1. Characteristics of adolescents* between 10 and 19 years old

	**Adolescent Population†**	**Adolescents with emergency admission(s) for injury between 10 and 19 years old, n (row %)**			
**Characteristics**	**Total**	**Total**	**AdvRI**	**AccRI Only**	**Other Causes**
**All**	**3,254,046**	**402,916 (100.0)**	**140,512 (34.9)**	**234,665 (58.2)**	**27,739 (6.9)**
**Age****					
**Girls**	**1,588,942**	**144,158 (100.0)**	**72,768 (50.5)**	**59,528 (41.3)**	**11,862 (8.2)**
10-14 years	.	65,208 (100.0)	23,289 (35.7)	37,303 (57.2)	4,616 (7.1)
15-17 years	.	48,286 (100.0)	31,620 (65.5)	12,882 (26.7)	3,784 (7.8)
18-19 years	.	30,664 (100.0)	17,859 (58.2)	9,343 (30.5)	3,462 (11.3)
**Boys**	**1,665,104**	**258,503 (100.0)**	**67,704 (26.2)**	**174,962 (67.7)**	**15,837 (6.1)**
10-14 years	.	121,821 (100.0)	17,660 (14.5)	97,511 (80.0)	6,650 (5.5)
15-17 years	.	79,223 (100.0)	25,004 (31.6)	49,366 (62.3)	4,853 (6.1)
18-19 years	.	57,459 (100.0)	25,040 (43.6)	28,085 (48.9)	4,334 (7.5)
Missing (Sex)	.	255 (100.0)	40 (15.7)	175 (68.6)	40 (15.7)
		**Adolescents with emergency admission(s) for injury between 10 and 19 years old, n (column %)**			
		**Total**	**AdvRI**	**AccRI Only**	**Other Causes**
**All**	**3,254,046**	**402,916 (100.0)**	**140,512 (100.0)**	**234,665 (100.0)**	**27,739 (100.0)**
**Chronic Condition**	**.**	**117,190 (29.0)**	**47,001 (33.4)**	**52,509 (22.4)**	**16,706 (60.0)**

For peer review only

For peer review only - http://bmjopen.bmj.com/site/about/guidelines.xhtml

200 AdvRI = Adversity-related Injury, AccRI Only = Accident-related Injury Only.
201 *Adolescents whose entire ten years of adolescence (ages 10-19) occurred in 1998-2011.
202 †Based on ONS mid-year England statistics for 10 year olds in 1998-2002.[20]
203 **At first emergency admission for injury

For peer review only

For peer review only - http://bmjopen.bmj.com/site/about/guidelines.xhtml

Types of injury and age at emergency admission

One-third of the cohort (140 152, 4.3% of the population) had a record of an emergency admission for adversity-related injury between 10 and 19 years old (the AdvRI group; 153 633 emergency admissions for adversity-related injury) (Table 1), with similar rates between sexes (72 768, 4.3% girls in the population; 67 704, 4.1% boys).

The remaining two-thirds of the cohort (262 404, 8.1% of the adolescent population) had emergency admissions for injury which were never related to adversity (Table 1). Among these adolescents, 234 665 (89.4%) had an accident-related injury (the AccRI Only group, 7.2% of the population) and 27 739 (10.6%) had no accident-related injury (Other Causes group, 0.9% of the population). A higher proportion of the Other Causes group were affected by a chronic condition between 10 and 19 years old (16 706/27 739, 60%), compared with the AdvRI group (47 001/140 512, 33%) or AccRI Only group (52 509/234 665, 22%).

These groupings (AdvRI [and subgroups], AccRI, Other Causes) are provided in Supplementary Table 3, as proportions of adolescents in the general population and within individual age groups.

Types of adversity-related injury

Among adolescents in the AdvRI group (girls 72 768, boys 67,704) (Figure 1), the most common type of adversity was drug or alcohol misuse (girls 91.1%, boys 58.0%). A higher proportion of boys than girls were exposed to victimisation (girls 17.5%, boys 51.7%), but a higher proportion of girls than boys were exposed to self-harm (girls 74.6%; boys 31.0%).

For peer review only - http://bmjopen.bmj.com/site/about/guidelines.xhtml

Girls in the AdvRI group were most likely to be exposed to multiple types of adversity between 10 and 19 years old (73.6%) (Figure 1), especially SH+DA (61.7%, i.e., most of the 73.6%). Fewer boys in the AdvRI group were exposed to multiple types of adversity (36.2%), the most common combination also being SH+DA (22.7%).

For most of the adolescents who exposed to multiple types of adversity, the combination of types was recorded at the same admission. For example, among the 352 adolescent girls who were exposed to victimisation and self-harm between 10 and 19 years old (Figure 1), 85.3% had both victimisation and self-harm codes present simultaneously at at least one emergency admission for injury (V+DA 86.1%, SH+ DA 99.7%, V+ SH+ DA 83.2%; boys: V+SH 63.2%, V+DA 84.5%, SH+DA 99.0%, V+SH+DA 59.1%) (data not shown).

Emergency readmissions for injury

Adolescent girls in the AdvRI group (50.5% of all girls in the cohort) accounted for 59.9% of the total number of emergency admissions for injury coming from girls (data not shown), compared with girls in the AccRI Only group (41.3% of all girls) who accounted for 15.1%. Boys in the AdvRI group (26.2% of all boys in the cohort) accounted for 29.6% compared with 61.7% contributed by boys in the AccRI Only group (67.7% of all boys).

More adolescents in the AdvRI group were readmitted for injury (i.e., had two or more emergency admissions for injury) between 10 and 19 years old (both girls and boys 17%) (Figure 2), than in the AccRI Only group (girls 5%; boys 7%). Among adolescents admitted for AdvRIs related to multiple

For peer review only - http://bmjopen.bmj.com/site/about/guidelines.xhtml

252 types of adversity (Table 2), the proportion readmitted was even higher
253 (multiple types: girls 21%, boys 24%; single type: girls 7%, boys 12%).

For peer review only

For peer review only - http://bmjopen.bmj.com/site/about/guidelines.xhtml

Table 2. Proportion of adolescent girls and boys with 1, 2 or 3+ emergency admission(s) for injury or 1, 2 or 3+ admissions of any type, by types of adversity between 10 and 19 years old*

	Girls (%)							Boys (%)						
		Emergency admission(s) for injury			Admission(s) of any type				Emergency admission(s) for injury			Admission(s) of any type		
**Adolescent group***	**No. girls**	**1**	**2**	**3+**	**1**	**2**	**3+**	**No. boys**	**1**	**2**	**3+**	**1**	**2**	**3+**
**All**	**144,158**	**88.6**	**8.3**	**3.1**	**57.6**	**20.5**	**22.0**	**258,503**	**90.3**	**8.0**	**1.8**	**68.5**	**18.7**	**12.9**
**AdvRI**	**72,768**	**82.6**	**12.0**	**5.3**	**53.8**	**21.0**	**25.2**	**67,704**	**83.4**	**12.5**	**4.1**	**64.7**	**19.4**	**15.9**
Any V	12,710	73.1	15.7	11.2	46.7	21.0	32.3	35,003	82.6	12.9	4.5	64.9	19.4	15.7
Any SH	54,315	79.3	13.9	6.8	51.2	21.5	27.3	21,087	76.7	15.8	7.5	57.0	20.7	22.3
Any DA	66,255	81.8	12.5	5.7	53.5	21.1	25.4	39,264	80.9	13.7	5.4	62.6	19.6	17.8
**Single adversity**	**19,194**	**92.9**	**6.1**	**1.0**	**61.2**	**19.6**	**19.2**	**43,214**	**87.6**	**10.2**	**2.2**	**68.5**	**18.8**	**12.8**
V Only	4,047	91.8	6.7	1.5	57.6	20.2	22.2	25,908	87.0	10.7	2.3	68.1	19.2	12.7
SH Only	2,114	90.8	7.7	1.5	54.4	20.8	24.8	2,188	87.6	10.0	2.4	62.8	19.6	17.6
DA Only	13,033	93.5	5.7	0.8	63.4	19.3	17.3	15,118	88.5	9.4	2.1	69.9	17.9	12.2

For peer review only

For peer review only - http://bmjopen.bmj.com/site/about/guidelines.xhtml

		Girls (%)							Boys (%)					
		Emergency admission(s) for injury			Admission(s) of any type				Emergency admission(s) for injury			Admission(s) of any type		
**Adolescent group***	**No. girls**	**1**	**2**	**3+**	**1**	**2**	**3+**	**No. boys**	**1**	**2**	**3+**	**1**	**2**	**3+**
**Multiple adversity**	**53,574**	**79.0**	**14.2**	**6.9**	**51.1**	**21.5**	**27.3**	**24,490**	**76.1**	**16.5**	**7.5**	**58.0**	**20.7**	**21.4**
V + SH	352	80.4	14.8	4.8	49.7	23.0	27.3	344	71.8	21.8	6.4	53.8	24.4	21.8
V + DA	1,373	84.6	13.0	2.3	54.0	22.4	23.6	5,591	78.3	16.4	5.3	63.4	20.1	16.5
SH + DA	44,911	81.8	13.1	5.1	52.9	21.6	25.5	15,395	79.7	14.8	5.5	59.1	21.0	19.8
V + SH + DA	6,938	59.5	21.5	19.0	38.7	21.2	40.2	3,160	54.8	24.2	21.0	43.0	19.5	37.4
No adversity	71,390	94.7	4.5	0.8	61.4	19.9	18.7	190,799	92.7	6.4	0.9	69.8	18.4	11.8
**AccRI Only**	**59,528**	**95.3**	**4.1**	**0.6**	**66.6**	**18.7**	**14.7**	**174,962**	**92.6**	**6.5**	**0.9**	**71.4**	**18.1**	**10.5**
**Other Causes**	**11,862**	**91.6**	**6.3**	**2.1**	**35.4**	**25.8**	**38.7**	**15,837**	**93.4**	**5.0**	**1.6**	**51.7**	**22.1**	**26.2**

V = Victimisation, SH = Self-harm, DA = Drug or Alcohol Misuse, AdvRI = Adversity-related Injury, AccRI Only = Accident-related Injury Only.

* Each adolescent classified by all adversities/accidents seen at any emergency admission(s) for injury between 10 and 19 years old.

For peer review only

Similarly, a higher proportion of adolescents in the AdvRI group had two more admissions of any type (including non-emergency and non-injury) between 10 and 19 years old (girls 36.2%; boys 35.3%; Table 2) compared with adolescents in the AccRI Only group (girls 33.4%; boys 28.6%). This proportion was even higher for adolescents in the AdvRI group who were admitted with multiple types of adversity (multiple types: girls 48.8%, boys 42.1%; single type: girls 38.8%, boys 31.6%).

For peer review only

For peer review only - http://bmjopen.bmj.com/site/about/guidelines.xhtml

DISCUSSION

More than one in 20 adolescents in England had at least one emergency admission for adversity-related injury between 10 and 19 years old. These adolescents accounted for a third of all adolescents with emergency admissions for injury and for a disproportionate number of re-admissions for injury, particularly adolescents admitted with multiple types of adversity-related injury. Targeting adolescents admitted with adversity-related injury could reduce their risk of future harm and the rate of re-admissions to hospital, which are some of the most expensive types of healthcare and the UK government is keen to reduce.[25]

Longitudinally-linked admissions allowed us to study the entire ten years of adolescence in 402 916 adolescents, to distinguish between types of adversity that co-occurred during adolescence or at the same admission, and to study re-admissions.

One weakness of this study was our reliance on diagnostic codes recorded in administrative data. Maltreatment and drug or alcohol misuse have been shown to be under-recorded using ICD-10,[26 27] but false positives are rare.[15] To address under-recording, we used what we considered to be sensitive clusters of codes for adversity. Other factors related to recording or coding practices,[12 14 15 27 28] for example, new guidelines for defining maltreatment,[14] can also affect ascertainment. Because of the relative insensitivity but good specificity of the coding clusters, some adolescents who were classified in the AccRI group may in fact belong to the AdvRI group, but did not have their adversity recognised or recorded. Consequently, our prevalence estimates of admission for different types of

For peer review only - http://bmjopen.bmj.com/site/about/guidelines.xhtml

adversity-related injury are likely to provide a lower bound for the true prevalence.

Our prevalence estimates of admission for injury related to individual types of adversity from the general adolescent population are consistent with previous reports for emergency admissions for assault-related injury in 2004-2009 and for all admissions (emergency and non-emergency) for self-harm and drug or alcohol misuse.[1 2 24 29] Previous studies have reported higher rates of drug or alcohol misuse in boys than in girls in the general adolescent population.[24] We found higher rates in girls. This difference could indicate that girls exposed to drug or alcohol misuse are more likely to be injured, present to hospital, or be admitted after presenting to hospital, as a result. Our estimated rates of re-admission of any type (including non-emergency and non-injury) for victimisation (girls 53.3%, boys 35.1%) (Table 2) and self-harm (girls 48.8%, boys 43.0%) were higher than previously reported (11% for victimisation, 33% for self-harm).[3 4] These discrepancies are likely to be because we considered the whole ten years of adolescence and re-admission of any type, whereas previous studies looked at re-attendance up to the following year and for the same type of adversity-related injury.[3 4]

The results of this study should inform policy initiatives and national guidelines. Firstly, a substantial proportion of adolescents are affected by adversity and they account for a large proportion (29.6-59.9%) of all emergency admissions to hospital for injury in this age group. Secondly, we show the large burden of injury admission for all three types of adversity, yet there are currently no national clinical guidelines for managing assault. Finally, these results show that adolescents often present with multiple types

For peer review only - http://bmjopen.bmj.com/site/about/guidelines.xhtml

For peer review only

of adversity (especially in girls), even though guidelines exist only for managing individual problems.[30-32]

In addition, policy makers need to be aware of the widely varying aetiological pathways to admission with adversity-related injury. Our approach to defining this group of adolescents is not designed to reflect the complexity or severity of these cases. For example, admission for multiple types of adversity-related injury is a poor proxy indicator of severity. Effective interventions will need to be tailored to the individual, based on specialist clinical assessment. However, all three types of adversity are likely to reflect a combination of underlying psychosocial need and environmental and social stressors. This intervention may reduce the risk of future harm including the incidence of other types of harm not seen in hospital, e.g., further adversity-related injury not leading to admission, that are likely to be more frequent in these adolescents, and which the hospital administrative data we used does not capture. Further work in admissions data linked to other administrative datasets, e.g., accident and emergency data, could tell us if the risk of these other types of harm is also increased in adolescents admitted with adversity-related injury.

For peer review only

For peer review only - http://bmjopen.bmj.com/site/about/guidelines.xhtml

Contributors

Annie Herbert - helped to conceive and design the study, analysed and interpreted the data, drafted the article, revised it critically for important intellectual content and approved the final version to be published.

Leah Li - helped to conceive and design the study, interpreted the data, revised the article critically for important intellectual content and approved the final version to be published.

Arturo González-Izquierdo - helped to conceive and design the study, acquired and interpreted the data, revised the article critically for important intellectual content and approved the final version to be published.

Ruth Gilbert – helped to conceive and design the study, acquired and interpreted the data, revised the article critically for important intellectual content and approved the final version to be published.

Competing interests None.

Funding AH was supported by the Policy Research Unit in the Health of Children, Young People and Families, which is funded by the Department of Health Policy Research Programme (grant reference number: 109/0001). This report is an independent research commissioned by the Department of Health. The views expressed in this publication are those of the authors and not necessarily those of the Department of Health. AH is also supported by the University College London Impact studentship. The study sponsors played no part in the design, data analysis and interpretation of this study; the

For peer review only - http://bmjopen.bmj.com/site/about/guidelines.xhtml

writing of the manuscript or the decision to submit the paper for publication, and the authors' work was independent of their funders.

Ethics Both Hospital Episode Statistics (HES) admissions data and Office for National Statistics (ONS) mid-year population estimates are derived from routinely collected administrative data. HES data were pseudonymised before we received them and therefore we did not require Research Ethics Committee approval.

Data sharing No additional data available.

For peer review only

REFERENCES

1. Bellis MA, Hughes K, Wood S, et al. National five-year examination of inequalities and trends in emergency hospital admission for violence across England. Injury prevention : journal of the International Society for Child and Adolescent Injury Prevention 2011;**17**(5):319-25 doi: 10.1136/ip.2010.030486[published Online First: Epub Date]|.

2. Hawton K, Rodham K, Evans E, et al. Deliberate self harm in adolescents: self report survey in schools in England. Bmj 2002;**325**(7374):1207-11

3. Meuleners LB, Lee AH, Hendrie D. Interpersonal violence hospitalisations for adolescents: A population-based study. Journal of paediatrics and child health 2010;**46**(11):686-90 doi: 10.1111/j.1440-1754.2010.01818.x[published Online First: Epub Date]|.

4. Lilley R, Owens D, Horrocks J, et al. Hospital care and repetition following self-harm: multicentre comparison of self-poisoning and self-injury. The British Journal of Psychiatry 2008;**192**(6):440-45

5. Snider C, Lee J. Youth violence secondary prevention initiatives in emergency departments: a systematic review. Cjem 2009;**11**(2):161-8

6. Ougrin D, Boege I, Stahl D, et al. Randomised controlled trial of therapeutic assessment versus usual assessment in adolescents with self-harm: 2-year follow-up. Archives of disease in childhood 2013;**98**(10):772-6 doi: 10.1136/archdischild-2012-303200[published Online First: Epub Date]|.

7. Crawford MJ, Patton R, Touquet R, et al. Screening and referral for brief intervention of alcohol-misusing patients in an emergency department: a pragmatic randomised controlled trial. The Lancet;**364**(9442):1334-39 doi: http://dx.doi.org/10.1016/S0140-6736(04)17190-0[published Online First: Epub Date]|.

8. Johnson SB, Bradshaw CP, Wright JL, et al. Characterizing the teachable moment: is an emergency department visit a teachable moment for intervention among assault-injured youth and their parents? Pediatric emergency care 2007;**23**(8):553-59

9. Inpatient HES Data Dictionary. Leeds: Health and Social Care Information Centre, 2010.

For peer review only

For peer review only - http://bmjopen.bmj.com/site/about/guidelines.xhtml

10. World Health Organisation. Adolescent Health. Secondary Adolescent Health 2013. http://www.who.int/topics/adolescent_health/en/.
11. National Health Service Act, 2006.
12. Gonzalez-Izquierdo A, Woodman J, Copley L, et al. Variation in recording of child maltreatment in administrative records of hospital admissions for injury in England, 1997-2009. Archives of disease in childhood 2010;**95**(11):918-25 doi: 10.1136/adc.2009.180216[published Online First: Epub Date]|.
13. International Statistical Classification of Diseases and Related Health Problems 10th Revision. Secondary International Statistical Classification of Diseases and Related Health Problems 10th Revision. http://apps.who.int/classifications/icd10/browse/2010/en.
14. Saperia J, Lakhanpaul M, Kemp A, et al. When to suspect child maltreatment: summary of NICE guidance. Bmj 2009;**339**:b2689 doi: 10.1136/bmj.b2689[published Online First: Epub Date]|.
15. Gonzalez-Izquierdo A, Ward A, M OD, et al. Cross-country comparison of victimisation-related injury admission in children and adolescents in England and Western Australia. BMC Health Serv Res 2013;**13**(1):260 doi: 10.1186/1472-6963-13-260[published Online First: Epub Date]|.
16. Patton GC, Viner R. Pubertal transitions in health. Lancet 2007;**369**(9567):1130-9 doi: 10.1016/S0140-6736(07)60366-3[published Online First: Epub Date]|.
17. Gill T. How old are GCSE candidates? Statistics Report Series, 2010.
18. Directgov. AS and A levels. Secondary AS and A levels 2012. http://webarchive.nationalarchives.gov.uk/20121015000000/http://www.direct.gov.uk/en/EducationAndLearning/QualificationsExplained/DG_10039018.
19. Buying Alcohol. Secondary Buying Alcohol 2013. https://www.drinkaware.co.uk/check-the-facts/alcohol-and-the-law/buying-alcohol.
20. All releases of Population Estimates for UK, England and Wales, Scotland and Northern Ireland: Office for National Statistics, 2013.
21. Office for National Statistics. Mid-year population estimates short methods guide: Office for National Statistics, 2010.
22. Radford L. Child abuse and neglect in the UK today. NSPCC Research: NSPCC, 2011.

For peer review only - http://bmjopen.bmj.com/site/about/guidelines.xhtml

23. *Truth hurts: report of the national inquiry into self-harm among young people*. London: Mental Health Foundation, 2006.
24. Hibell B, Guttormsson U, Ahlström S, et al. The 2011 ESPAD Report. Substance use among students in 2012;**36**
25. National Audit Office. Emergency admissions to hospital: managing the demand, 2013.
26. Scott D, Tonmyr L, Fraser J, et al. The utility and challenges of using ICD codes in child maltreatment research: A review of existing literature. Child abuse & neglect 2009;**33**(11):791-808 doi: S0145-2134(09)00202-6 [pii]
10.1016/j.chiabu.2009.08.005[published Online First: Epub Date]|.
27. McKenzie K, Harrison JE, McClure RJ. Identification of alcohol involvement in injury-related hospitalisations using routine data compared to medical record review. Australian and New Zealand journal of public health 2010;**34**(2):146-52 doi: 10.1111/j.1753-6405.2010.00499.x[published Online First: Epub Date]|.
28. Farrar S, Yi D, Sutton M, et al. Has payment by results affected the way that English hospitals provide care? Difference-in-differences analysis. Bmj 2009;**339** doi: 10.1136/bmj.b3047[published Online First: Epub Date]|.
29. Hawton K, Rodham K, Evans E, et al. Adolescents Who Self Harm: A Comparison of Those Who Go to Hospital and Those Who Do Not. Child Adol Ment H-Uk 2009;**14**(1):24-30 doi: DOI 10.1111/j.1475-3588.2008.00485.x[published Online First: Epub Date]|.
30. *Self-harm. The short term physical and psychological management and secondary prevention of self-harm in primary and secondary care.* London: National Institute for Health and Clinical Excellence, 2011.
31. *Drug misuse: Psychosocial interventions*. London: National Institute for Health and Clinical Excellence, 2007.
32. *Alcohol-use disorder : diagnosis, assessment and management of harmful drinking and alcohol dependence*. London: National Institute for Health and Clinical Excellence, 2011.

For peer review only

For peer review only - http://bmjopen.bmj.com/site/about/guidelines.xhtml

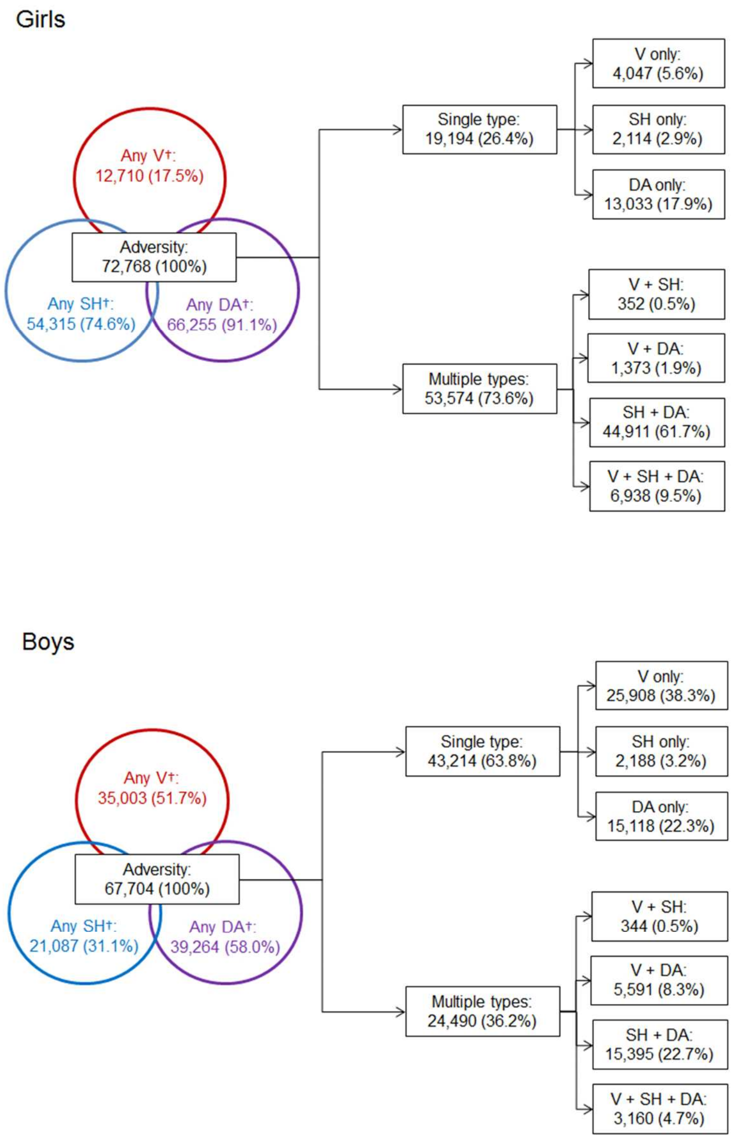

Figure 1. Number (%) of adolescents with adversity-related injury, by types of adversity between 10 and 19 years old* and sex.

V = victimisation, SH = self-harm, DA = drug or alcohol misuse

*Each adolescent classified by all adversity recorded at any emergency admission(s) for injury between 10 and 19 years old.

†'Any V', 'Any SH' and 'Any DA' are not mutually exclusive.
214x315mm (96 x 96 DPI)

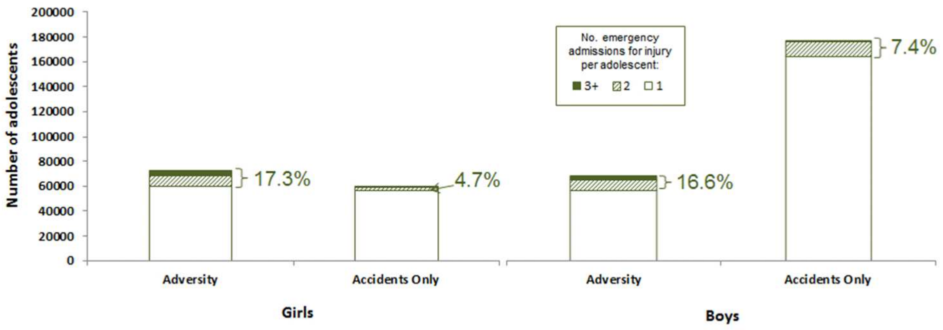

Figure 2. Number of adolescents with 1, 2 and 3 or more emergency admission(s) for injury between 10 and 19 years old*, by types of injury between 10 and 19 years old† and sex

AdvRI = Adversity-related Injury, AccRI Only = Accident-related Injury Only

*Percentages are of adolescents who have 2 or more emergency admissions for injury between 10-19 years old.

†Each adolescent classified by all adversity/accidents recorded at any emergency admission(s) for injury.
251x104mm (96 x 96 DPI)

review only

Supplementary Tables

Supplementary Table 1. Cohort (shaded) who have at least one emergency admission for injury between 10 and 19 years old in 1998-2011 (N = 402,916)

1998	*1999*	*2000*	*2001*	*2002*	…	*2007*	*2008*	*2009*	*2010*	*2011*
6	7	8	9	10	…	15	16	17	18	19
7	8	9	10	11	…	16	17	18	19	20
8	9	10	11	12	…	17	18	19	20	21
9	10	11	12	13	…	18	19	20	21	22
10	11	12	13	14	…	19	20	21	22	23

For peer review only

Supplementary Table 2. Definitions and International Classification of Diseases (ICD-10) diagnostic codes used to classify emergency admissions for injury, chronic conditions and types of adversity*

ADMISSIONS AND ADMISSION TYPES

Admission

Admissions were defined by linked episodes of inpatient care, including day cases (any case > 4 hours stay). Any admission which occurred within one day or was a hospital transfer from a previous admission was considered to be the same admission. An episode may have up to 20 diagnosis codes entered, but 88% of admissions in this cohort had episodes with four or less diagnoses recorded (data not shown).

Emergency (acute; unplanned) admission

An admission was considered to be an emergency if the method of admission variable (‘admimeth’) was 21-24 or 28.(1)

Injury

An admission was considered to be for injury if any of the diagnosis codes in the first episode of the admission were S or T codes (Chapter XIX of the ICD -10).

CHRONIC CONDITIONS

ICD-10 codes as per Hardelid et al, 2013,(2) excluding E24.4, F10-F19, G31.2, G40.5, G62.1, G72.1, K29.2, K70, K85.2, K86.0, O35.4, R78.1-R78.5, Z50.2, Z50.3, Z86.4, X64-X84, Y10-Y34, Z91.5, F30.0-F30.2, F30.8-F32.3, F32.8-F334, F33.8-F34.1, F34.8-F34.9, F38.0-F38.1, F38.8, F39-F40.2, F40.8- F41.3, F41.8-F41.9.

ADVERSITY CLUSTERS AND DESCRIPTIONS*	ICD-10 CODES
**Victimisation**	
Maltreatment	
Maltreatment syndromes	T74

For peer review only - http://bmjopen.bmj.com/site/about/guidelines.xhtml

Effects of other deprivation (extreme neglect)	T73
Perpetrator of neglect and other maltreatment syndromes	Y06, Y07
Assault	
Assault by bodily force and sexual assault	Y04, Y05
Other types of assault	X85 - Y03, Y08 - Y09
Undetermined cause	
Events of undetermined intent	Y20 - Y34
Examination and observation following other inflicted injury	Z04.5
Examination and observation for other reasons: request for expert evidence	Z04.8
Adverse social circumstances	
Neonatal withdrawal symptoms from maternal use of drugs of addiction	P96.1
Other problems related to physical environment	Z58.8
Problem related to physical environment, unspecified	Z58.9
Homelessness	Z59.0
Inadequate housing	Z59.1
Lack of adequate food	Z59.4
Extreme poverty	Z59.5
Insufficient social insurance and welfare support	Z59.7
Problem related to housing and economic circumstances, unspecified	Z59.9
Problems related to social environment	Z60
Problems related to negative life events in childhood	Z61
Other problems related to upbringing	Z62

For peer review only

Other problems related to primary support group	Z63
Discord with counsellors	Z64.4
Problems related to other legal circumstances	Z65.3
Other specified problems related to psychosocial circumstances	Z65.8
Problem related to unspecified psychosocial circumstances	Z65.9
Problems related to lifestyle	Z72.3 - Z72.9
Problems related to care-provider dependency	Z74
Health supervision and care of foundling	Z76.1
Health supervision and care of other healthy infant and child	Z76.2
Family history of mental and behavioural disorders	Z81
Personal history of other specified risk-factors, not elsewhere classified	Z91.8
**Self-harm**	
Intentional self-poisoning by and exposure to…	
…drugs	X60-X63
…other and unspecified drugs, medicaments and biological substances	X64
…alcohol	X65
…organic solvents and halogenated hydrocarbons and their vapours	X66
…other gases and vapours	X67

For peer review only

…pesticides	X68
…other and unspecified chemicals and noxious substances	X69
Intentional self-harm by…	
…hanging, strangulation and suffocation	X70
…drowning and submersion	X71
…firearm discharge	X72-X74
…explosive material	X75
…smoke, fire and flames, or steam, hot vapours and hot objects	X76-X77
…sharp/blunt objects	X78-X79
...jumping from a high place	X80
…jumping or lying before a moving object, or crashing a motor vehicle	X81-82
…other specified means	X83
…unspecified means	X84
Personal history of self-harm	Z91.5
**Alcohol/drug misuse**	
Alcohol	
Alcohol-induced pseudo-Cushing's syndrome	E24.4
Mental and behavioural disorders due to use of alcohol	F10
Degeneration of nervous system due to alcohol	G31.2
Alcoholic polyneuropathy	G62.1
Alcoholic myopathy	G72.1

For peer review only

Alcoholic cardiomyopathy	I42.1
Alcoholic gastritis	K29.2
Alcoholic liver disease	K70
Alcohol-induced acute pancreatitis	K85.2
Alcohol-induced chronic pancreatitis	K86.0
Maternal care for (suspected) damage to fetus from alcohol	O35.4
Fetus and newborn affected by maternal use of alcohol†	P04.3
Fetal alcohol syndrome (dysmorphic)†	Q86.0
Finding of alcohol in blood	R78.0
Poisoning: antidotes and chelating agents, not elsewhere classsified	T50.6
Toxic effect of alcohol	T51
Accidental poisoning by exposure to alcohol	X45
Intentional self-poisoning by and exposure to alcohol**	X65
Poisoning by exposure to alcohol, undetermined intent	Y15
Evidence of alcohol involvement determined by blood alcohol level	Y90
Evidence of alcohol involvement determined by level of intoxication‡	Y91
Alcohol rehabilitation	Z50.2
Alcohol abuse counselling and surveillance	Z71.4
Alcohol use‡	Z72.1
Drugs, medicaments and biological substances (illicit drugs)	
Mental and behavioural disorders due to psychoactive	F11 - F17, F19

substance use	
~~Maternal care for (suspected) damage to fetus by drugs~~[†]	O35.5
~~Neonatal jaundice due to drugs or toxins transmitted from mother or given to newborn~~[†]	~~P58.4~~
~~Neonatal withdrawal symptoms from maternal use of drugs of addiction~~[†]	~~P96.1~~
Finding of drugs not normally found in blood	R78.1 - R78.5
Poisoning by drugs, medicaments and biological substances	T36 - T50 (not inc. T50.6)
~~Intentional self-poisoning by and exposure to antiepileptic, sedative-hypnotic, antiparkinsonism and psychotropic drugs, not elsewhere classified~~[**]	~~X61~~
~~Intentional self-poisoning by and exposure to other drugs acting on the autonomic nervous system~~[**]	~~X63~~
~~Intentional self-poisoning by and exposure to other and unspecified drugs, medicaments and biological substances~~[**]	~~X64~~
~~Assault by drugs, medicaments and biological substances~~[§]	~~X85~~
Poisoning, undetermined intent	Y10 - Y14
Drug rehabilitation	Z50.3
Drug abuse counselling and surveillance	Z71.5
Drug use	Z72.2
Environmental/ Domestic substances	
Mental and behavioural disorders due to use of volatile solvents	F18
Accidental poisoning by and exposure to noxious	X40 – X44, X46 - X49

For peer review only

substances	
~~Intentional self-poisoning by and exposure to organic solvents and halogenated hydrocarbons and their vapours~~‡	~~X66~~
Intentional self-poisoning by and exposure to noxious substances	X69
~~Assault by unspecified chemical or noxious substance~~§	~~X90~~
Poisoning by chemical or noxious substance, undetermined intent	Y16 - Y19
Codes mentioning both alcohol and drugs	
Special epileptic syndromes - (related to alcohol, drugs, etc.)	G40.5
Blood-alcohol and blood-drug test	Z04.0
~~Personal history of psychoactive substance abuse - (alcohol, drug, tobacco)~~***	~~Z86.4~~
**Accidents**	
Transport accidents	V01 - V99
Falls	W00 - W19
Exposure to inanimate mechanical forces	W20 - W49
Exposure to animate mechanical forces	W50 - W64
Accidental drowning and submersion	W65 - W74
Other accidental threats to breathing	W75 - W84
Exposure to electric current, radiation and extreme ambient air temperature and pressure	W85 - W99
Exposure to smoke, fire and flames	X00 - X09

For peer review only

Contact with heat and hot substances	X10 - X19
Contact with venomous animals and plants	X20 - X29
Exposure to forces of nature	X30 - X39
Accidental poisoning by and exposure to noxious substances	X40 - X49
Overexertion, travel and privation	X50 - X57
Accidental exposure to other and unspecified factors	X58 - X59

* 'Strikethrough' (~~Phrase~~, ~~code)~~ indicates that this was not included in the adversity definition.
† Codes relating to foetuses or neonates (P04.3, Q86.0, P58.4 and P96) were not expected in adolescent presentations; this was checked and did not appear.
** Formed part of the self-harm cluster only; self-poisoning considered most egregious element of diagnosis.
‡ If subject under 18 years old.
§ Formed part of the victimisation cluster only; assault considered most egregious element of the diagnosis.
***Not possible to distinguish whether for drugs, alcohol or tobacco

For peer review only

Supplementary Table 3. Population prevalence* of emergency admission(s) for injury, by types of adversity within age periods† and sex

	All girls	All boys	Girls			Boys		
**Adolescent group†**	**10-19y**	**10-19y**	**10-14y**	**15-17y**	**18-19y**	**10-14y**	**15-17y**	**18-19y**
All	8.66	16.27‡	3.92	3.12§	2.11§	7.67‡	5.29‡§	0.40‡§
**AdvRI**	**4.37**	**4.26‡**	**1.31**	**2.04§**	**1.27§**	**0.72‡**	**1.53‡§**	**1.74‡§**
Any Victimisation	0.76	2.20‡	0.25	0.31§	0.19§	0.33‡	0.80‡§	0.90‡§
Any Self-harm	3.26	1.33‡	0.90	1.58§	0.97§	0.22‡	0.47‡§	0.55‡§
Any Drug or Alcohol Misuse	3.98	2.47‡	1.16	1.87§	1.15§	0.39‡	0.84‡§	1.01‡§
**Single adversity**	**1.15**	**2.72‡**	**0.44**	**0.49§**	**0.32§**	**0.53‡**	**0.99‡§**	**1.08‡§**
Victimisation Only	0.24	1.63‡	0.09	0.10§	0.08§	0.28‡	0.64‡§	0.69‡§
Self-harm Only	0.13	0.14	0.05	0.06§	0.03§	0.05	0.04‡§	0.04‡
Drug or Alcohol Misuse Only	0.78	0.95‡	0.29	0.33§	0.21§	0.21‡	0.31‡§	0.35‡§

For peer review only

	All girls	All boys	Girls			Boys		
**Adolescent group†**	**10-19y**	**10-19y**	**10-14y**	**15-17y**	**18-19y**	**10-14y**	**15-17y**	**18-19y**
**Multiple adversity**	**3.22**	**1.54‡**	**0.88**	**1.55§**	**0.95§**	**0.19‡**	**0.54‡§**	**0.66‡§**
V+SH	0.02	0.02	0.01	0.01	0.004§	0.005‡	0.01§	0.01‡
V+DA	0.08	0.35‡	0.03	0.03	0.02§	0.02‡	0.11‡§	0.15‡§
SH+DA	2.70	0.97‡	0.72	1.34§	0.84§	0.14‡	0.37‡§	0.45‡§
V+SH+DA	0.42	0.20‡	0.12	0.17§	0.09§	0.02‡	0.05‡§	0.06‡§
No adversity	4.29	12.01‡	2.79	1.08§	0.84§	6.95‡	3.76‡§	2.26‡§
**AccRI Only**	**0.71**	**1.00‡**	**0.32**	**0.26§**	**0.24§**	**0.47‡**	**0.36‡§**	**0.31‡§**
**Other Causes**	**3.58**	**11.01‡**	**2.47**	**0.82§**	**0.60§**	**6.48‡**	**3.40‡§**	**1.95‡§**

V = Victimisation, SH = Self-harm, DA = Drug or Alcohol Misuse, AdvRI = Adversity-related Injury, AccRI Only = Accident-related Injury Only

*Due to large denominators, confidence intervals for population prevalence were too narrow to provide any meaningful interpretation and so are not shown.

†Each adolescent classified by all adversity/accidents seen at any emergency injury admission(s) at 10-19 years, 10-14 years, 15-17 years and 18-19 years, respectively.

‡Statistically significantly different from girls of the same age (according to 95% confidence intervals).

§Statistically significantly different from 10-14 year olds of the same sex (according to 95% confidence intervals).

For peer review only

REFERENCES

1.Inpatient HES Data Dictionary. Leeds: Health and Social Care Information Centre, 2010.
2.Hardelid P DN, Davey J, Primbramska I, Gilbert R. Overview of child deaths in the four UK countries. London: 2013.

For peer review only

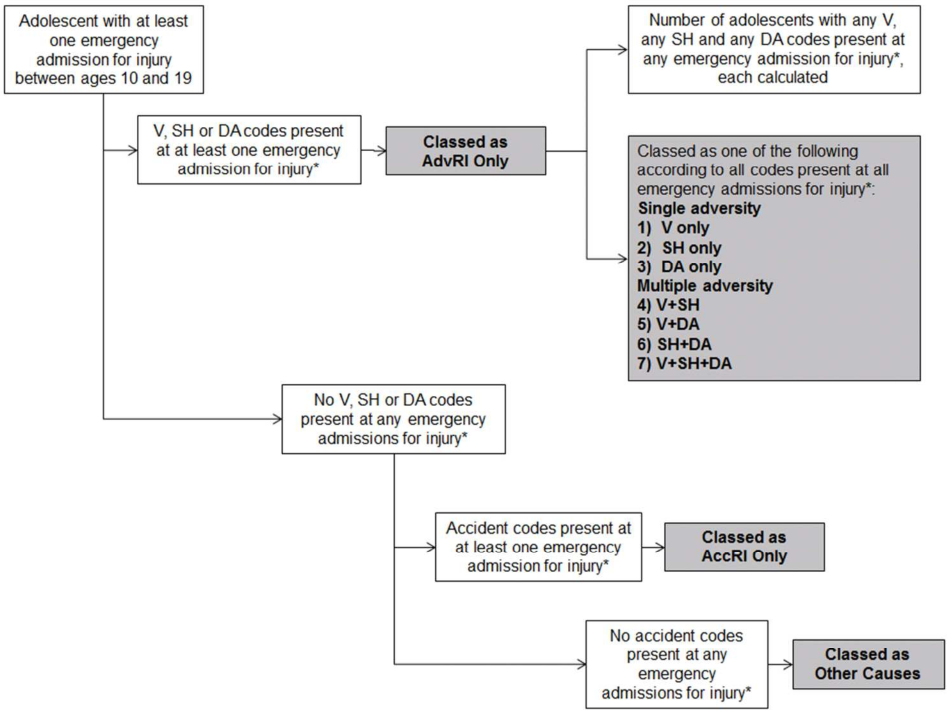

Supplementary Figure 1. Classification of groups of adolescents.

V = victimisation, SH = self-harm, DA = Drug or alcohol misuse, AdvRI = Adversity-related injury, AccRI Only = Accident-related injury only.

*between ages 10 and 19.
262x201mm (96 x 96 DPI)

only

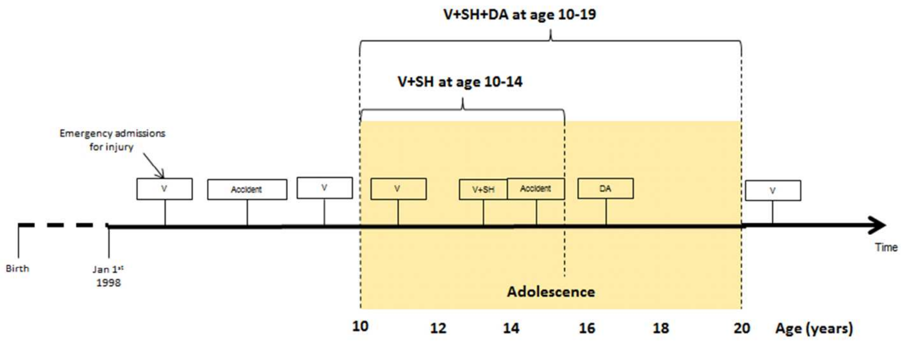

Supplementary Figure 2. Example of classification of groups of adolescents within age groups and across adolescence (ages 10-19).

V = victimisation, SH = self-harm, DA = drug or alcohol misuse.
254x102mm (96 x 96 DPI)

STROBE Statement—checklist of items that should be included in reports of observational studies

	Item No	Recommendation
**Title and abstract**	1	(*a*) Indicate the study's design with a commonly used term in the title or the abstract
		(*b*) Provide in the abstract an informative and balanced summary of what was done and what was found
**Introduction**		
Background/rationale	2	Explain the scientific background and rationale for the investigation being reported
Objectives	3	State specific objectives, including any prespecified hypotheses
**Methods**		
Study design	4	Present key elements of study design early in the paper
Setting	5	Describe the setting, locations, and relevant dates, including periods of recruitment, exposure, follow-up, and data collection
Participants	6	(*a*) *Cohort study*—Give the eligibility criteria, and the sources and methods of selection of participants. Describe methods of follow-up<br>*Case-control study*—Give the eligibility criteria, and the sources and methods of case ascertainment and control selection. Give the rationale for the choice of cases and controls<br>*Cross-sectional study*—Give the eligibility criteria, and the sources and methods of selection of participants
		(*b*) *Cohort study*—For matched studies, give matching criteria and number of exposed and unexposed. N/A<br>*Case-control study*—For matched studies, give matching criteria and the number of controls per case
Variables	7	Clearly define all outcomes, exposures, predictors, potential confounders, and effect modifiers. Give diagnostic criteria, if applicable
Data sources/ measurement	8*	For each variable of interest, give sources of data and details of methods of assessment (measurement). Describe comparability of assessment methods if there is more than one group
Bias	9	Describe any efforts to address potential sources of bias. We only analysed those who could be observed between ages 10 and 19, so no bias due to different lengths of admission trajectories.
Study size	10	Explain how the study size was arrived at. All available administrative data.
Quantitative variables	11	Explain how quantitative variables were handled in the analyses. If applicable, describe which groupings were chosen and why
Statistical methods	12	(*a*) Describe all statistical methods, including those used to control for confounding
		(*b*) Describe any methods used to examine subgroups and interactions
		(*c*) Explain how missing data were addressed. N/A
		(*d*) *Cohort study*—If applicable, explain how loss to follow-up was addressed. We only analysed those who could be observed between ages 10 and 19.<br>*Case-control study*—If applicable, explain how matching of cases and controls was addressed<br>*Cross-sectional study*—If applicable, describe analytical methods taking account of sampling strategy
		(*e*) Describe any sensitivity analyses. N/A

Continued on next page

For peer review only - http://bmjopen.bmj.com/site/about/guidelines.xhtml

**Results**		
Participants	13*	~~(a) Report numbers of individuals at each stage of study—eg numbers potentially eligible, examined for eligibility, confirmed eligible, included in the study, completing follow-up, and analysed~~
		(b) Give reasons for non-participation at each stage. N/A
		(c) Consider use of a flow diagram. We didn't use a flow diagram as eligibility could be described using one sentence.
Descriptive data	14*	~~(a) Give characteristics of study participants (eg demographic, clinical, social) and information on exposures and potential confounders~~
		~~(b) Indicate number of participants with missing data for each variable of interest~~
		(c) *Cohort study*—Summarise follow-up time (eg, average and total amount). N/A
Outcome data	15*	~~*Cohort study*—Report numbers of outcome events or summary measures over time.~~ We state how many emergency admissions for adversity-related injury the AdvRI group had. We also provide a table indicating the number of emergency admissions for injury and admissions (of any type) during ages 10-19.
		Case-control study—Report numbers in each exposure category, or summary measures of exposure
		Cross-sectional study—Report numbers of outcome events or summary measures
Main results	16	~~(*a*) Give unadjusted estimates and, if applicable, confounder-adjusted estimates and their precision (eg, 95% confidence interval). Make clear which confounders were adjusted for and why they were included.~~ We gave prevalence estimates stratified by age-group and sex.
		~~(*b*) Report category boundaries when continuous variables were categorized~~
		(*c*) If relevant, consider translating estimates of relative risk into absolute risk for a meaningful time period. N/A
Other analyses	17	~~Report other analyses done—eg analyses of subgroups and interactions,~~ and sensitivity analyses
**Discussion**		
Key results	18	~~Summarise key results with reference to study objectives~~
Limitations	19	~~Discuss limitations of the study, taking into account sources of potential bias or imprecision. Discuss both direction and magnitude of any potential bias~~
Interpretation	20	~~Give a cautious overall interpretation of results considering objectives, limitations, multiplicity of analyses, results from similar studies, and other relevant evidence~~
Generalisability	21	~~Discuss the generalisability (external validity) of the study results~~
**Other information**		
Funding	22	~~Give the source of funding and the role of the funders for the present study and, if applicable, for the original study on which the present article is based~~

*Give information separately for cases and controls in case-control studies and, if applicable, for exposed and unexposed groups in cohort and cross-sectional studies.

Note: An Explanation and Elaboration article discusses each checklist item and gives methodological background and published examples of transparent reporting. The STROBE checklist is best used in conjunction with this article (freely available on the Web sites of PLoS Medicine at http://www.plosmedicine.org/, Annals of Internal Medicine at http://www.annals.org/, and Epidemiology at http://www.epidem.com/). Information on the STROBE Initiative is available at www.strobe-statement.org.

For peer review only

For peer review only - http://bmjopen.bmj.com/site/about/guidelines.xhtml

BMJ Open

Violence, self-harm and drug or alcohol misuse in adolescents admitted to hospitals in England for injury: a retrospective cohort study

Journal:	*BMJ Open*
Manuscript ID:	bmjopen-2014-006079.R1
Article Type:	Research
Date Submitted by the Author:	29-Dec-2014

Complete List of Authors:	Herbert, Anne; UCL Institute of Child Health, Population, Policy and Practice Programme<br>Gilbert, Ruth; UCL Institute of Child Health, Population, Policy and Practice Programme<br>Gonzalez-Izquierdo, Arturo; UCL Institute of Child Health, Population, Policy and Practice Programme<br>Li, Leah; UCL Institute of Child Health, Population, Policy and Practice Programme

**Primary Subject Heading**:	Paediatrics
Secondary Subject Heading:	Mental health, Health services research
Keywords:	MENTAL HEALTH, Non-accidental injury < PAEDIATRICS, Substance misuse < PSYCHIATRY, Suicide & self-harm < PSYCHIATRY

SCHOLARONE™
Manuscripts

For peer review only

Violence, self-harm and drug or alcohol misuse in adolescents admitted to hospitals in England for injury: a retrospective cohort study

Annie Herbert, Centre for Paediatric Epidemiology & Biostatistics, University College London Institute of Child Health, 30 Guilford Street, London, WC1N 1EH, Tel: +44207 905 2790, Fax: +44207 905 2793, annie.herbert.12@ucl.ac.uk

Annie Herbert, M.Sc.[1], Ruth Gilbert, M.D.[1], Arturo González-Izquierdo, Ph.D.[1], Leah Li, Ph.D.[1]

1. Centre for Paediatric Epidemiology & Biostatistics, University College London Institute of Child Health, 30 Guilford Street, London, WC1N 1EH, UK

Short title: Adversity in adolescents admitted to hospital for injury

Key words: Wounds and Injuries, Violence, Self-injurious Behaviour, Substance-related Disorders, Adolescent

Word count: 2,565

No. figures: 2

No tables: 2

No references: 35

No supplementary files for online only publication: 5

For peer review only - http://bmjopen.bmj.com/site/about/guidelines.xhtml

ABSTRACT

Objectives

Of adolescents in the general population in England, we aimed to determine 1) the proportion that has an emergency admission to hospital for injury related to adversity (violence, self-harm or drug or alcohol misuse) and 2) the risk of recurrent emergency admissions for injury in adolescents admitted with adversity-related injury compared with those admitted with accident-related injury only.

Design

We used longitudinally-linked administrative hospital data (Hospital Episode Statistics) to identify 10-19 year olds with emergency admissions for injury (including day cases lasting more than four hours), in England in 1998-2011. We used Office for National Statistics mid-year estimates for population denominators.

Results

Approximately 4.3% (n=141 248) of adolescents in the general population (n=3 254 046) had one or more emergency admissions for adversity-related injury (girls 4.6%, boys 4.1%), accounting for 50.0% of all emergency admissions for injury in girls and 29.1% in boys. Admissions for self-harm or drug or alcohol misuse commonly occurred in the same girls and boys. Recurrent emergency admissions for injury were more common in adolescents with adversity-related injury (girls 17.3%; boys 16.5%) than in those with accident-related injury only (girls 4.7%; boys 7.4%), particularly for

For peer review only - http://bmjopen.bmj.com/site/about/guidelines.xhtml

adolescents with adversity-related injury related to multiple types of adversity (girls 21.1%; boys 24.2%).

Conclusion

Hospital-based interventions should be developed to reduce the risk of future injury in adolescents admitted for adversity-related injury.

For peer review only

For peer review only - http://bmjopen.bmj.com/site/about/guidelines.xhtml

What is already known on this subject

- Adolescents exposed to adversity (violence, self-harm or drug or alcohol misuse) often use health services, and repetitively.
- Many adolescents in the general population are exposed to multiple types of adversity during their adolescence.

What this study adds

- One in 20 adolescents in England has an emergency admission to hospital for adversity-related injury between 10 and 19 years old.
- Many adolescents admitted with adversity-related injury (particularly girls) are admitted with multiple types of adversity, self-harm and drug or alcohol misuse being the most common combination.
- Re-admission is more common in these adolescents than those admitted with accident-related injury only.

Strengths and limitations of this study

- Hospital Episodes Statistics (HES) captured data on all admissions to National Health Service hospitals in England at 10-19 years old in this study's cohort.
- The longitudinal link between admissions for each individual in HES data allowed us to study the burden of multiple emergency admissions for injury over time.
- However, violence, self-harm and drug or alcohol misuse are not always recognised at an admission, or consistently recorded, and

For peer review only - http://bmjopen.bmj.com/site/about/guidelines.xhtml

therefore this study's estimates of prevalence of adversity are likely to be underestimates.

For peer review only

For peer review only - http://bmjopen.bmj.com/site/about/guidelines.xhtml

INTRODUCTION

Many adolescents exposed to adversity such as violence, self-harm, or drug or alcohol misuse use secondary health services,[1 2] often repetitively.[3 4] For example, in a self-report survey of 15-16 year olds in England, 12.6% of those who had self-harmed had presented to hospital.[2] It is also estimated that approximately one-third of patients attending a hospital in England for self-harm re-attend for self-harm in the following year.[4] Improved management of adolescents exposed to adversity could reduce risk of repetition as well as the burden on secondary care.[5-7]

An admission to hospital provides the 'teachable moment'.[8] That is, both adolescents and their families may be more likely to engage with an intervention than if they had received it elsewhere. Hospital-based interventions to reduce the risk of future harm could benefit these adolescents by reducing episodes of injury, and may reduce recurrent emergency (i.e., acute or unplanned) admissions for injury.

To date, there is a lack of evidence on how different types of adversity-related injury occur in the same adolescents over time. In addition, policy makers and service providers need to know how many adolescents have an emergency admission to hospital for adversity-related injury, their characteristics and their specific rates of re-admission if they are to be feasibly targeted for intervention.

In this study we used administrative hospital data and Office for National Statistics (ONS) mid-year population estimates to estimate the number of adolescents in the general population who have ever had an emergency admission to hospital for injury. We then estimated the

For peer review only - http://bmjopen.bmj.com/site/about/guidelines.xhtml

prevalence of emergency admissions for injury related to violence, self-harm and drug or alcohol misuse (alone and co-occurring) in the general population. Finally, we determined the risk of recurrent emergency admissions for injury in adolescents who had at least one admission between 10 and 19 years for adversity-related injury compared with adolescents only ever admitted for accidental injury during the same period.

For peer review only

For peer review only - http://bmjopen.bmj.com/site/about/guidelines.xhtml

METHODS

Study population

Using administrative data from all admissions to National Health Service hospitals in England (Hospital Episode Statistics [HES]) in 1998-2011,[9] we derived a retrospective cohort of adolescents who turned 10 years old in 1998-2002, who could be observed throughout adolescence until 19 years old (Supplementary Table 1).[10] Each individual also had to have at least one emergency admission for injury between 10 and 19 years old.

Admission data

The Health and Social Care Information Centre provided pseudonymised data on hospital admissions, use of which did not require Research Ethics Committee approval.[11] An admission is defined by the National Health Service as any hospital case lasting longer than four hours, and so includes long day cases as well as overnight stays. We analysed any hospital transfers or admissions within one day after discharge as the same admission, as previously described.[12] We used the variable for method of admission ('admimeth') to define an emergency admission. We used all International Classification of Diseases-10 (ICD-10) diagnosis codes recorded during an admission to categorise admissions as being for injury related to adversity or an accident (Supplementary Table 2).[13]

Types of injury and age at emergency admission

We defined an emergency admission for injury as being related to adversity, comprising violence (maltreatment/assault/undetermined causes of injury),

For peer review only - http://bmjopen.bmj.com/site/about/guidelines.xhtml

self-harm or drug or alcohol misuse, using mutually exclusive clusters of ICD-10 codes (Supplementary Table 2). Violence was defined by previously-validated codes, which would trigger consideration of violence by carers, peers or strangers.[12 14 15] We defined self-harm using codes that mentioned either 'self-harm' or 'self-poisoning'. Drug or alcohol misuse was defined by codes that mentioned 'alcohol', 'drugs', 'noxious substance' or 'solvent'. We defined an injury as being related to accidents only if no adversity codes were present but there were codes from the ICD-10 *Accidents* subchapter (V01-X59).[13]

We grouped age at each admission to reflect age of onset of puberty (10-14 years), age of sitting secondary school exams (15-17 years) and the legal age for buying alcohol (18-19 years).[16-19]

Classification of adolescents according to types of injury and age at emergency admissions

We classed adolescents into groups according to all of their emergency admissions for injury between 10 and 19 years old. Adolescents were classed as belonging to the 'adversity' group (any adversity-related injury between 10 and 19 years old), 'accidents only' group (no adversity-related injury but one or more accident-related injuries) or 'other causes' group (no adversity-related or accident-related injuries) (Supplementary Figure 1). Among adolescents in the adversity group, we determined the proportion exposed to violence-, self-harm- and drug or alcohol misuse-related injury at age 10-19, respectively. We further classified the adversity group into seven mutually exclusive sub-groups: violence only, self-harm only, drug or alcohol misuse only, violence and self-harm, violence and drug or alcohol misuse,

For peer review only - http://bmjopen.bmj.com/site/about/guidelines.xhtml

self-harm and drug or alcohol misuse, and violence, self-harm and drug or alcohol misuse.

We also grouped adolescents as above, according to their emergency admissions for injury at 10-14 years only, 15-17 years only and 18-19 years only (Supplementary Figure 2).

Population denominators

We used ONS mid-year population estimates to derive population denominators.[20 21] These data are freely available online broken down by sex and year of age.

Analyses

We estimated the proportion of adolescents in the general population who had an emergency admission for injury between 10 and 19 years old. We used the number of adolescents in our derived retrospective cohort as the numerator and ONS mid-year estimates for 10 year olds in 1998-2002 as the population denominator. We then calculated these proportions by types of injury at 10-19 years old (adversity-related [adversity group and seven mutually exclusive subgroups] and accidents only [accidents only group]) and by age group, as described above.

We calculated the proportion of adolescents in the adversity group (and subgroups) and in the accidents only group who had an emergency admission for injury twice and three or more times between 10 and 19 years old. We also calculated the proportions of adolescents with two or three or more admissions of any type (including non-emergency and non-injury).

We reported all results separately for girls and boys since differences between girls and boys have been reported for prevalence of adversity in the

For peer review only - http://bmjopen.bmj.com/site/about/guidelines.xhtml

For peer review only

general population.[1 22-24] We calculated 95% confidence intervals for all proportions but did not present them here as they were all too narrow to convey any useful information (within one unit of the sample estimate). Analyses were carried out in StataSE 12.

For peer review only

For peer review only - http://bmjopen.bmj.com/site/about/guidelines.xhtml

RESULTS

There were 1 033 702 adolescents in HES admissions data in 1998-2011, of which 402 916 formed the study cohort (462 476 emergency admission for injury when considering multiple presentations from the same adolescent, 802 682 admissions of any type [including non-emergency and non-injury, 662 727 of which were overnight stays]) (Table 1), representing 12.4% (402 916/3 254 046) of the adolescent population. Twice as many boys as girls had an emergency admission for injury during adolescence (144 158/1 588 942 girls in the population [8.7%]; 258 503/1 665 104 boys [16.3%]).

For peer review only

Table 1. Characteristics of adolescents whose entire ten years of adolescence (ages 10-19) occurred in 1998-2011.

	**Adolescent population***	**Adolescents with emergency admission(s) for injury between 10 and 19 years old, n (row %)**			
**Characteristics**	**Total**	**Total**	**Adversity**	**Accidents only**	**Other causes**
**All**	**3,254,046**	**402,916 (100.0)**	**141,248 (35.1)**	**233,907 (58.1)**	**27,761 (6.9)**
**Age****					
**Girls**	**1,588,942**	**144,158 (100.0)**	**72,805 (50.5)**	**59,528 (41.3)**	**11,888 (8.2)**
10-14 years	.	65,208 (100.0)	23,178 (35.5)	37,388 (57.3)	4,642 (7.1)
15-17 years	.	48,286 (100.0)	31,573 (65.4)	12,922 (26.8)	3,791 (7.9)
18-19 years	.	30,664 (100.0)	18,054 (58.9)	9,155 (29.9)	3,455 (11.3)
**Boys**	**1,665,104**	**258,503 (100.0)**	**68,403 (26.5)**	**174,267 (67.4)**	**15,833 (6.1)**
10-14 years	.	121,821 (100.0)	17,667 (14.5)	97,478 (80.0)	6,676 (5.5)
15-17 years	.	79,223 (100.0)	25,014 (31.6)	49,345 (62.3)	4,864 (6.1)
18-19 years	.	57,459 (100.0)	25,722 (44.8)	27,444 (47.8)	4,293 (7.5)
Missing (Sex)	.	255 (100.0)	40 (15.7)	175 (68.6)	40 (15.7)

*Based on ONS mid-year England statistics for 10 year olds in 1998-2002.[20]
**At first emergency admission for injury

For peer review only

Types of injury and age at emergency admission

One-third of the cohort (141 248, 4.3% of the population) had a record of an emergency admission for adversity-related injury between 10 and 19 years old (the adversity group; 157 004 emergency admissions for adversity-related injury in total) (Table 1), with similar rates between sexes (72 805, 4.6% girls in the population; 68 403, 4.1% boys).

The remaining two-thirds of the cohort (261 668, 8.1% of the adolescent population) had emergency admissions for injury which were never related to adversity (Table 1). Among these adolescents, 233 907 (89.4%) had an accident-related injury (the accidents only group, 7.2% of the population) and 27 761 (10.6%) had no accident-related injury (other causes group, 0.9% of the population). A high proportion of the other causes group were affected by a chronic condition[1] between 10 and 19 years old (11 221/27 761, 40.4%), compared with the adversity group (45 321/141 248, 32.1%) or accidents only group (49 434/233 907, 21.1%).

Proportions of adolescents in the general population and within individual age groups by adversity [and subgroups], accidents only, and other causes groups are provided in Supplementary Table 3.

Types of adversity-related injury

Among adolescents in the adversity group (girls 72 805, boys 68 403) (Figure 1), the most common type of adversity was drug or alcohol misuse (girls 91.5%, boys 60.0%). A higher proportion of boys than girls were exposed to

[1] Defined by ICD-10 codes (Supplementary Table 2)

For peer review only - http://bmjopen.bmj.com/site/about/guidelines.xhtml

violence (girls 8.5%, boys 47.6%), but a higher proportion of girls than boys were exposed to self-harm (girls 74.6%; boys 32.9%).

Girls in the adversity group were most likely to be exposed to multiple types of adversity between 10 and 19 years old (69.2% + 2.0% + 1.2% + 0.2% = 72.6%) (Figure 1), especially self-harm and drug or alcohol misuse (69.2% of the entire adversity group, i.e., most of the 72.6%). Fewer boys in the adversity group were exposed to multiple types of adversity (38.4%), the most common combination also being self-harm and drug or alcohol misuse (24.8%).

For most of the adolescents who were exposed to multiple types of adversity, the combination of types was recorded at the same admission. For example, among the 130 adolescent girls who were exposed to violence and self-harm between 10 and 19 years old (Table 2), 64.6% had both violence and self-harm codes present simultaneously in at least one emergency admission for injury (violence and drug or alcohol misuse 78.8%, self-harm and drug or alcohol misuse 99.7%, violence, self-harm and drug or alcohol misuse 33.9%; boys: violence and self-harm 40.1%, violence and drug or alcohol misuse 84.1%, self-harm and drug or alcohol misuse 99.1%, violence, self-harm and drug or alcohol misuse 20.0%) (data not shown).

For peer review only - http://bmjopen.bmj.com/site/about/guidelines.xhtml

Table 2. Proportion of adolescents with 1, 2 or 3+ emergency admission(s) for injury or 1, 2 or 3+ admission(s) of any type, by types of adversity between 10 and 19 years old*

		Girls (%)							Boys (%)					
		Emergency admission(s) for injury			Admission(s) of any type				Emergency admission(s) for injury			Admission(s) of any type		
**Adolescent group***	**No. girls**	**1**	**2**	**3+**	**1**	**2**	**3+**	**No. boys**	**1**	**2**	**3+**	**1**	**2**	**3+**
**All**	**144,158**	**88.6**	**8.3**	**3.1**	**57.6**	**20.5**	**22.0**	**258,503**	**90.3**	**8.0**	**1.8**	**68.5**	**18.7**	**12.9**
**Adversity**	**72,805**	**82.7**	**12.0**	**5.3**	**53.8**	**21.1**	**25.1**	**68,403**	**83.5**	**12.4**	**4.1**	**64.8**	**19.4**	**15.8**
Any violence	6,211	77.2	13.9	8.9	49.1	20.7	30.1	32,799	83.2	12.8	4.0	65.6	19.6	14.8
Any self-harm	54,315	79.3	13.9	6.8	51.2	21.5	27.3	21,087	76.7	15.8	7.5	57.0	20.7	22.3
Any drug or alcohol misuse	66,645	81.9	12.5	5.6	53.6	21.1	25.3	41,014	81.1	13.6	5.3	62.9	19.5	17.6
**Single adversity**	**19,924**	**92.8**	**6.2**	**1.0**	**61.2**	**19.8**	**19.0**	**43,563**	**71.3**	**8.3**	**1.8**	**55.8**	**15.2**	**10.3**
Violence only	3,734	92.4	6.3	1.3	58.1	20.4	21.6	24,912	87.1	10.6	2.2	68.3	19.2	12.6
Self-harm only	2,296	90.3	8.1	1.6	54.4	20.7	24.9	2,260	87.3	10.4	2.3	62.3	19.8	17.9
Drug or alcohol misuse only	13,894	93.3	5.9	0.8	63.2	19.5	17.4	16,391	88.5	9.5	2.1	69.9	17.9	12.2
**Multiple adversity**	**52,881**	**78.9**	**14.2**	**6.9**	**51.0**	**21.6**	**27.4**	**24,840**	**77.3**	**16.7**	**7.5**	**59.0**	**21.0**	**21.5**
V + SH	130	70.0	20.8	9.2	42.3	26.2	31.5	217	41.6	15.4	6.1	32.8	16.0	14.2

For peer review only

For peer review only - http://bmjopen.bmj.com/site/about/guidelines.xhtml

V + DA	862	81.9	15.1	3.0	52.2	22.9	24.9	6,013	84.6	17.5	5.4	68.4	21.8	17.3
SH + DA	50,404	80.1	13.7	6.3	51.8	21.6	26.6	16,953	86.7	16.4	6.9	64.6	22.7	22.9
V + SH + DA	1,485	36.8	31.6	31.5	25.5	19.9	54.5	1,657	22.0	16.4	14.1	17.9	11.7	22.8
No adversity	71,353	94.7	4.5	0.8	61.4	19.9	18.7	190,100	92.4	6.3	0.9	69.5	18.3	11.8
**Accidents only**	**59,465**	**95.3**	**4.1**	**0.6**	**66.5**	**18.7**	**14.7**	**174,267**	**92.3**	**6.5**	**0.9**	**71.1**	**18.0**	**10.5**
**Other causes**	**11,888**	**91.5**	**6.3**	**2.1**	**35.5**	**25.8**	**38.8**	**15,833**	**93.4**	**5.0**	**1.6**	**51.5**	**22.1**	**26.3**

243 V = Violence, SH = Self-harm, DA = Drug or Alcohol Misuse
244 * Each adolescent classified by all adversity/accidents seen at any emergency admission(s) for injury between 10 and 19 years old.

For peer review only

Emergency re-admissions for injury

Adolescent girls in the adversity group (50.5% of all girls in the cohort) accounted for 50.0% of the total number of emergency admissions for injury coming from girls (data not shown), compared with girls in the accidents only group (41.3% of all girls) who accounted for 36.6%. Boys in the adversity group (26.2% of all boys in the cohort) accounted for 29.1% compared with 65.0% contributed by boys in the accidents only group (67.7% of all boys).

More adolescents in the adversity group were re-admitted for injury (i.e., had two or more emergency admissions for injury) between 10 and 19 years old (girls 17.3%, boys 16.5%) (Figure 2), than in the accidents only group (girls 4.7%; boys 7.4%). Among adolescents admitted for injuries related to multiple types of adversity (Table 2), the proportion re-admitted was even higher (multiple types: girls 21.1%, boys 24.2%; single type: girls 7.2%, boys 10.1%).

Similarly, a higher proportion of adolescents in the adversity group had two more admissions of any type (including non-emergency and non-injury) between 10 and 19 years old (girls 46.2%; boys 35.2%) (Table 2) compared with adolescents in the accidents only group (girls 33.4%; boys 28.5%). This proportion was even higher for adolescents in the adversity group who were admitted with multiple types of adversity (multiple types: girls 49.0%, boys 42.5%; single type: girls 38.8%, boys 25.5%).

For peer review only - http://bmjopen.bmj.com/site/about/guidelines.xhtml

DISCUSSION

More than one in 20 adolescents in England had at least one emergency admission for adversity-related injury between 10 and 19 years old. These adolescents accounted for a third of all adolescents with emergency admissions for injury and for a disproportionate number of re-admissions for injury, particularly adolescents admitted with multiple types of adversity-related injury. Targeting adolescents admitted with adversity-related injury could reduce their risk of future harm, the rate of re-admissions to hospital, and health care costs .[25]

Longitudinally-linked admissions allowed us to study the entire ten years of adolescence in 402 916 individuals. We were able, to distinguish between types of adversity that co-occurred during adolescence or at the same admission, and to study re-admissions. One weakness of this study was our reliance on diagnostic codes recorded in administrative data. Violence by carers, which could be coded under maltreatment, and drug or alcohol misuse have been shown to be under-recorded using ICD-10,[26 27] but false positives are rare.[15] To address under-recording, we used what we considered to be sensitive clusters of codes for adversity. Other factors related to recording or coding practices,[12 14 15 27 28] for example, new guidelines for defining maltreatment,[14] can also affect ascertainment. Because of the relative insensitivity but good specificity of the coding clusters, some adolescents who were classified in the accidents only group may in fact belong to the adversity group, but did not have their adversity recognised or recorded. Consequently, our prevalence estimates of admission for different

For peer review only - http://bmjopen.bmj.com/site/about/guidelines.xhtml

types of adversity-related injury are likely to provide a lower bound for the true prevalence.

Our prevalence estimates of admission for injury related to individual types of adversity from the general adolescent population are consistent with previous reports for emergency admissions for assault-related injury in 2004-2009 and for all admissions (emergency and non-emergency) for self-harm and drug or alcohol misuse.[1 2 24 29] Previous studies have reported higher rates of drug or alcohol misuse in boys than in girls in the general adolescent population.[24] We found higher rates in girls. This difference could indicate that girls exposed to drug or alcohol misuse are more likely to be injured, to present to hospital, or to be admitted after a hospital presentation, than boys.

Our estimated rates of re-admission of any type (including non-emergency and non-injury) for violence (girls 50.8%, boys 34.4%) (Table 2) and self-harm (girls 48.8%, boys 43.0%) were higher than previously reported (11% for violence, 33% for self-harm).[3 4] These discrepancies are likely to be because we considered the whole ten years of adolescence and re-admission of any type, whereas previous studies looked at re-attendance up to the following year and for the same type of adversity-related injury.[3 4]

The results of this study should inform policy initiatives and national guidelines. Firstly, a substantial proportion of adolescents are affected by adversity and they account for a large proportion (29.1 to 50.0%) of all emergency admissions to hospital for injury in this age group. Secondly, we show the large burden of injury admission for all three types of adversity, yet there are currently no national clinical guidelines for managing cases of violence, other than responding to violence by caregivers.[30] Finally, these

For peer review only - http://bmjopen.bmj.com/site/about/guidelines.xhtml

results show that adolescents often present with multiple types of adversity (especially in girls), even though guidelines exist only for managing individual problems.[31-33]

In addition, policy makers need to be aware of the widely varying aetiological pathways to admission with adversity-related injury. Our approach to defining this group of adolescents is not designed to reflect the complexity or severity of these cases. For example, admission for multiple types of adversity-related injury is a poor proxy indicator of severity. Effective interventions will need to be tailored to the individual based on specialist clinical assessment. However, all three types of adversity are likely to reflect a combination of underlying psychosocial need and environmental and social stressors.[34]

Hospital interventions may reduce the risk of future harm, including the incidence of other types of harm not seen in hospital, e.g., further adversity-related injury not leading to admission. Further research using linked data from health care sectors such as accident and emergency, could shed light on the overall burden of adversity-related injury on hospitals. Such analyses are limited by the quality of accident and emergency data in England (available since 2007) and the resulting problems with identifying reasons for presentation and accurately linking individuals to long-term outcomes.[35] However, longitudinally-linked datasets in other countries could provide insights into long-term outcomes for this vulnerable group of adolescents.

For peer review only - http://bmjopen.bmj.com/site/about/guidelines.xhtml

Contributors

Annie Herbert – helped to conceive and design the study, analysed and interpreted the data, drafted the article, revised it critically for important intellectual content and approved the final version to be published.

Leah Li – helped to conceive and design the study, interpreted the data, revised the article critically for important intellectual content and approved the final version to be published.

Arturo González-Izquierdo – helped to conceive and design the study, acquired and interpreted the data, revised the article critically for important intellectual content and approved the final version to be published.

Ruth Gilbert – helped to conceive and design the study, acquired and interpreted the data, revised the article critically for important intellectual content and approved the final version to be published.

Competing interests None.

Funding AH was supported by the Policy Research Unit in the Health of Children, Young People and Families, which is funded by the Department of Health Policy Research Programme (grant reference number: 109/0001). This report is an independent research commissioned by the Department of Health. The views expressed in this publication are those of the authors and not necessarily those of the Department of Health. AH is also supported by the University College London IMPACT studentship. RG is supported by awards establishing the Farr Institute of Health Informatics Research at University College London Partners from the Medical Research Council and a

For peer review only

For peer review only - http://bmjopen.bmj.com/site/about/guidelines.xhtml

consortium of funders (MR/K006584/1). The study funders played no part in the design, data analysis and interpretation of this study; the writing of the manuscript or the decision to submit the paper for publication, and the authors' work was independent of their funders.

Ethics Both Hospital Episode Statistics (HES) admissions data and Office for National Statistics (ONS) mid-year population estimates are derived from routinely collected administrative data. HES data were pseudonymised before we received them and therefore we did not require Research Ethics Committee approval.

Data sharing No additional data on HES are available. ONS mid-year population estimates may be accessed freely online: http://www.ons.gov.uk/ons/publications/all-releases.html?definition=tcm%3A77-22371.

For peer review only

For peer review only - http://bmjopen.bmj.com/site/about/guidelines.xhtml

REFERENCES

1. Bellis MA, Hughes K, Wood S, et al. National five-year examination of inequalities and trends in emergency hospital admission for violence across England. Injury prevention : journal of the International Society for Child and Adolescent Injury Prevention 2011;**17**(5):319-25 doi: 10.1136/ip.2010.030486[published Online First: Epub Date]|.
2. Hawton K, Rodham K, Evans E, et al. Deliberate self harm in adolescents: self report survey in schools in England. Bmj 2002;**325**(7374):1207-11
3. Meuleners LB, Lee AH, Hendrie D. Interpersonal violence hospitalisations for adolescents: A population-based study. Journal of paediatrics and child health 2010;**46**(11):686-90 doi: 10.1111/j.1440-1754.2010.01818.x[published Online First: Epub Date]|.
4. Lilley R, Owens D, Horrocks J, et al. Hospital care and repetition following self-harm: multicentre comparison of self-poisoning and self-injury. The British Journal of Psychiatry 2008;**192**(6):440-45
5. Snider C, Lee J. Youth violence secondary prevention initiatives in emergency departments: a systematic review. Cjem 2009;**11**(2):161-8
6. Ougrin D, Boege I, Stahl D, et al. Randomised controlled trial of therapeutic assessment versus usual assessment in adolescents with self-harm: 2-year follow-up. Archives of disease in childhood 2013;**98**(10):772-6 doi: 10.1136/archdischild-2012-303200[published Online First: Epub Date]|.
7. Crawford MJ, Patton R, Touquet R, et al. Screening and referral for brief intervention of alcohol-misusing patients in an emergency department: a pragmatic randomised controlled trial. The Lancet;**364**(9442):1334-39 doi: http://dx.doi.org/10.1016/S0140-6736(04)17190-0[published Online First: Epub Date]|.
8. Johnson SB, Bradshaw CP, Wright JL, et al. Characterizing the teachable moment: is an emergency department visit a teachable moment for intervention among assault-injured youth and their parents? Pediatric emergency care 2007;**23**(8):553-59
9. Inpatient HES Data Dictionary. Leeds: Health and Social Care Information Centre, 2010.

For peer review only - http://bmjopen.bmj.com/site/about/guidelines.xhtml

10. World Health Organisation. Adolescent Health. Secondary Adolescent Health 2013. http://www.who.int/topics/adolescent_health/en/.
11. National Health Service Act, 2006.
12. Gonzalez-Izquierdo A, Woodman J, Copley L, et al. Variation in recording of child maltreatment in administrative records of hospital admissions for injury in England, 1997-2009. Archives of disease in childhood 2010;**95**(11):918-25 doi: 10.1136/adc.2009.180216[published Online First: Epub Date]|.
13. International Statistical Classification of Diseases and Related Health Problems 10th Revision. Secondary International Statistical Classification of Diseases and Related Health Problems 10th Revision. http://apps.who.int/classifications/icd10/browse/2010/en.
14. Saperia J, Lakhanpaul M, Kemp A, et al. When to suspect child maltreatment: summary of NICE guidance. Bmj 2009;**339**:b2689 doi: 10.1136/bmj.b2689[published Online First: Epub Date]|.
15. Gonzalez-Izquierdo A, Ward A, M OD, et al. Cross-country comparison of victimisation-related injury admission in children and adolescents in England and Western Australia. BMC Health Serv Res 2013;**13**(1):260 doi: 10.1186/1472-6963-13-260[published Online First: Epub Date]|.
16. Patton GC, Viner R. Pubertal transitions in health. Lancet 2007;**369**(9567):1130-9 doi: 10.1016/S0140-6736(07)60366-3[published Online First: Epub Date]|.
17. Gill T. How old are GCSE candidates? Statistics Report Series, 2010.
18. Directgov. AS and A levels. Secondary AS and A levels 2012. http://webarchive.nationalarchives.gov.uk/20121015000000/http://www.direct.gov.uk/en/EducationAndLearning/QualificationsExplained/DG_10039018.
19. Buying Alcohol. Secondary Buying Alcohol 2013. https://www.drinkaware.co.uk/check-the-facts/alcohol-and-the-law/buying-alcohol.
20. All releases of Population Estimates for UK, England and Wales, Scotland and Northern Ireland: Office for National Statistics, 2013.
21. Office for National Statistics. Mid-year population estimates short methods guide: Office for National Statistics, 2010.
22. Radford L. Child abuse and neglect in the UK today. NSPCC Research: NSPCC, 2011.

For peer review only

For peer review only - http://bmjopen.bmj.com/site/about/guidelines.xhtml

23. *Truth hurts: report of the national inquiry into self-harm among young people*. London: Mental Health Foundation, 2006.
24. Hibell B, Guttormsson U, Ahlström S, et al. The 2011 ESPAD Report. Substance use among students in 2012;**36**
25. National Audit Office. Emergency admissions to hospital: managing the demand, 2013.
26. Scott D, Tonmyr L, Fraser J, et al. The utility and challenges of using ICD codes in child maltreatment research: A review of existing literature. Child abuse & neglect 2009;**33**(11):791-808 doi: S0145-2134(09)00202-6 [pii]
10.1016/j.chiabu.2009.08.005[published Online First: Epub Date]|.
27. McKenzie K, Harrison JE, McClure RJ. Identification of alcohol involvement in injury-related hospitalisations using routine data compared to medical record review. Australian and New Zealand journal of public health 2010;**34**(2):146-52 doi: 10.1111/j.1753-6405.2010.00499.x[published Online First: Epub Date]|.
28. Farrar S, Yi D, Sutton M, et al. Has payment by results affected the way that English hospitals provide care? Difference-in-differences analysis. Bmj 2009;**339** doi: 10.1136/bmj.b3047[published Online First: Epub Date]|.
29. Hawton K, Rodham K, Evans E, et al. Adolescents Who Self Harm: A Comparison of Those Who Go to Hospital and Those Who Do Not. Child Adol Ment H-Uk 2009;**14**(1):24-30 doi: DOI 10.1111/j.1475-3588.2008.00485.x[published Online First: Epub Date]|.
30. What to do if you're worried that a child is being abused. In: Skills DfE, ed.: DfES Publications, 2006.
31. *Self-harm. The short term physical and psychological management and secondary prevention of self-harm in primary and secondary care.* London: National Institute for Health and Clinical Excellence, 2011.
32. *Drug misuse: Psychosocial interventions*. London: National Institute for Health and Clinical Excellence, 2007.
33. Alcohol-use disorder : diagnosis, assessment and management of harmful drinking and alcohol dependence. London: National Institute for Health and Care Excellence, 2011.
34. Viner RM, Ozer EM, Denny S, et al. Adolescence and the social determinants of health. Lancet 2012;**379**(9826):1641-52 doi: 10.1016/S0140-6736(12)60149-4[published Online First: Epub Date]|.

For peer review only - http://bmjopen.bmj.com/site/about/guidelines.xhtml

35. Hospital Episode Statistics Accident & Emergency (England), 2007-. Secondary Hospital Episode Statistics Accident & Emergency (England), 2007-. http://adrn.ac.uk/catalogue/cataloguepage?sn=888040.

For peer review only

For peer review only - http://bmjopen.bmj.com/site/about/guidelines.xhtml

~~Victimisation~~Violence, self-harm and drug or alcohol misuse in adolescents admitted to hospitals in England for injury: a retrospective cohort study

Annie Herbert, Centre for Paediatric Epidemiology & Biostatistics, University College London Institute of Child Health, 30 Guilford Street, London, WC1N 1EH, Tel: +44207 905 2790, Fax: +44207 905 2793,
annie.herbert.12@ucl.ac.uk

Annie Herbert, M.Sc.[1]*, Ruth Gilbert, M.D.*[1]*, Arturo González-Izquierdo, Ph.D.*[1]*, Leah Li, Ph.D.*[1]

1. Centre for Paediatric Epidemiology & Biostatistics, University College London Institute of Child Health, 30 Guilford Street, London, WC1N 1EH, UK

Short title: Adversity in adolescents admitted to hospital for injury

Key words: Wounds and Injuries, Violence, Self-injurious Behaviour, Substance-related Disorders, Adolescent

Word count: 2,~~509~~565

No. figures: 2

No tables: 2

No references: 35~~2~~

No supplementary files for online only publication: 5~~5~~

For peer review only

ABSTRACT

Objectives

Of adolescents in the general population in England, we aimed to determine 1) the proportion that has an emergency admission to hospital for injury related to adversity (~~victimisation~~violence, self-harm or drug or alcohol misuse) and 2) the risk of recurrent emergency admissions for injury in adolescents admitted with adversity-related injury compared with those admitted with accident-related injury only.

Design

We used longitudinally-linked ~~hospital~~ administrative hospital data (Hospital Episode Statistics) to identify 10-19 year olds with emergency admissions for injury (including day cases lasting more than four hours), in England in 1998-2011. We used Office for National Statistics mid-year estimates for population denominators.

Results

Approximately 4.3% (n=141 248) of adolescents in the general population (n=3 254 046) had one or more emergency admissions for adversity-related injury (~~140 512/3 254 046;~~ girls 4.6%, boys 4.1%), accounting for ~~59.9~~50.0% of all emergency admissions for injury in girls and 29.~~6~~1% in boys. Admissions for self-harm or drug or alcohol misuse commonly occurred in the same girls and boys. Recurrent emergency admissions for injury were more common in adolescents with adversity-related injury (girls 17.3%; boys 16.5~~7~~%) than in those with accident-related injury only (girls ~~5~~4.7%; boys

For peer review only - http://bmjopen.bmj.com/site/about/guidelines.xhtml

7.4%), particularly for adolescents with adversity-related injury related to multiple types of adversity (girls 21.1%; boys 24.2%).

Conclusion

Hospital-based interventions should be developed to reduce the risk of future injury in adolescents admitted for adversity-related injury.

For peer review only

For peer review only - http://bmjopen.bmj.com/site/about/guidelines.xhtml

What is already known on this subject

- Adolescents exposed to adversity (~~victimisation~~<u>violence</u>, self-harm or drug or alcohol misuse) often use health services, and repetitively.
- Many adolescents in the general population are exposed to multiple types of adversity during their adolescence.

What this study adds

- One in 20 adolescents in England ha<u>s</u>~~ve~~ an emergency admission to hospital for adversity-related injury between 10 and 19 years old.
- Many adolescents admitted with adversity-related injury (particularly girls) are admitted with multiple types of adversity, self-harm and drug or alcohol misuse being the most common combination.
- Re<u>-</u>admission is more common in these adolescents than those admitted with accident-related injury only.

Strengths and limitations of this study

- Hospital Episodes Statistics (HES) captured data on all admissions to National Health Service hospitals in England at 10-19 years old in this study's cohort.
- The longitudinal link between admissions for each individual in HES data allowed us to study the burden of multiple emergency admissions for injury over time.
- However, ~~victimisation~~<u>violence</u>, self-harm and drug or alcohol misuse are not always recognised at an admission, or consistently recorded,

For peer review only - http://bmjopen.bmj.com/site/about/guidelines.xhtml

and therefore this study’s estimates of prevalence of adversity are likely to be underestimates.

For peer review only

For peer review only - http://bmjopen.bmj.com/site/about/guidelines.xhtml

INTRODUCTION

Many adolescents exposed to adversity such as victimisation violence(maltreatment or assault), self-harm, or drug or alcohol misuse use secondary health services,[1 2] often repetitively.[3 4] For example, in a self-report survey of 15-16 year olds in England, 12.6% of those who had self-harmed had presented to hospital.[2] It is also estimated that approximately one-third of patients attending a hospital in England for self-harm re-attend for self-harm in the following year.[4] Improved management of adolescents exposed to adversity could reduce risk of repetition as well as the burden on secondary care.[5-7]

An admission to hospital- provides the ‘teachable moment’,[8].[8] where That is, both adolescents and their families may be more likely to engage with an intervention than if they had received it elsewhere. Hospital-based interventions to reduce the risk of future harm could benefit these adolescents by reducing episodes of injury, and may reduce recurrent emergency (i.e., acute or unplanned) admissions for injury.

To -date, there is a lack of evidence on how different types of adversity--related injury occur in the same adolescents, over time. In addition, policy makers and service providers need to know how many adolescents have an emergency admission to hospital for adversity-related injury, their characteristics and their specific rates of re-admission if they are to be feasibly targeted for intervention.

In this study we used administrative hospital data and Office for National Statistics (ONS) mid-year population estimates to estimate the number of adolescents in the general population who have ever hadve an

For peer review only

For peer review only - http://bmjopen.bmj.com/site/about/guidelines.xhtml

emergency admission to hospital for injury. We then estimated the prevalence of emergency admissions for injury related to ~~victimisation~~violence, self-harm and drug or alcohol misuse (alone and co-occurring) in the general population. Finally, we determined the risk of recurrent emergency admissions for injury in adolescents who had at least one admission between 10 and 19 years for adversity-related injury compared with adolescents only ever admitted for accidental injury during the same period.

For peer review only

For peer review only - http://bmjopen.bmj.com/site/about/guidelines.xhtml

METHODS

Study population

Using administrative data from all admissions to National Health Service hospitals in England (Hospital Episode Statistics [HES]) in 1998-2011,[9] we derived a retrospective cohort of adolescents who turned 10 years old in 1998-2002, who could be observed throughout adolescence until 19 years old (Supplementary Table 1).[10] Each individual also had to have at least one emergency admission for injury between 10 and 19 years old.

Admission data

The Health and Social Care Information Centre provided pseudonymised data on hospital admissions, use of which did not require Research Ethics Committee approval.[11] An admission is defined by the National Health Service as any hospital case lasting longer than four hours, and so includes long day cases as well as overnight stays. We analysed any hospital transfers or admissions within one day after discharge as the same admission, as previously described.[12] We used the variable for method of admission ('admimeth') to define an emergency admission. We used all International Classification of Diseases-10 (ICD-10) diagnosis codes recorded during an admission to categorise admissions as being for injury related to adversity or~~,~~ an accident ~~or other causes~~ (Supplementary Table 2).[13]

Types of injury and age at emergency admission

We defined an emergency admission for injury as being ~~for~~ related to adversity, comprising ~~victimisation~~ violence

For peer review only

(maltreatment/assault/undetermined causes of injury), self-harm or drug or alcohol misuse, using mutually exclusive clusters of ICD-10 codes (Supplementary Table 2). Violence~~ctimisation~~ was defined by previously-validated codes, which would trigger consideration of ~~maltreatment~~ violence by carers~~ or of assault~~, peers or strangers.[12 14 15] We defined self-harm using codes that mentioned either 'self-harm' or 'self-poisoning'~~, and excluded 'undetermined intent'~~. Drug or alcohol misuse was defined by codes that mentioned 'alcohol', 'drugs', 'noxious substance' or 'solvent'. ~~We considered alcohol use (Z72.1) to be misuse only in adolescents younger than 18.~~ We defined an injury as being related to accidents only if no adversity codes were present but there were codes from the ICD-10 *Accidents* subchapter (V01-X59).[13]

We grouped age at each admission ~~as 10-14 years, 15-17 years and 18-19 years,~~ to reflect age of onset of puberty (10-14 years), age of sitting secondary school exams (~~15-18~~15-17 years) and the legal age for buying alcohol (18-19 years).[16-19]

Classification of adolescents according to types of injury and age at emergency admissions

We classed adolescents into groups according to all of their emergency admissions for injury between 10 and 19 years old. Adolescents were classed as belonging to the '~~AdvRI'~~adversity' group (any adversity-related injury between 10 and 19 years old), '~~AccRI~~accidents ~~Only'~~ only' group (no adversity-related injury but one or more accident-related injuries) or 'o~~O~~ther ~~C~~causes' group (no adversity-related or accident-related injuries) (Supplementary Figure 1). Among adolescents in the ~~AdvRI~~adversity group,

For peer review only - http://bmjopen.bmj.com/site/about/guidelines.xhtml

we determined the proportion exposed to ~~victimisation~~ violence-, self-harm- and drug or alcohol misuse-related injury at age 10-19, respectively. We further classified the ~~AdvRI~~adversity group into seven mutually exclusive sub-groups: ~~Victimisation~~ violence ~~Only~~only, ~~Self~~self-harm ~~Only~~only, ~~Drug~~ drug or ~~Alcohol~~ alcohol ~~Misuse~~ misuse ~~O~~only ~~(DA Only)~~, ~~Victimisation~~ violence and ~~Self~~self-harm ~~(V+SH)~~, ~~Victimisation~~ violence and ~~Drug~~ drug or ~~Alcohol~~ alcohol ~~M~~misuse ~~(V+DA)~~, ~~Self~~self-harm and ~~Drug~~ drug or ~~Alcohol~~ alcohol ~~Misuse~~ misuse, ~~(SH+ DA)~~ and ~~Victimisation~~ violence, ~~Self~~self-harm and ~~Drug~~ drug or ~~Alcohol~~ alcohol ~~M~~misuse ~~(V+SH+DA)~~.

We also grouped adolescents as above, according to their emergency admissions for injury at 10-14 years only, 15-17 years only and 18-19 years only (Supplementary Figure 2).

~~Defining an adolescent as having a chronic condition~~

~~We used a previously validated cluster of ICD-10 codes to identify adolescents who had been affected by a chronic condition in any admission between 10 and 19 years old (Supplementary Table 2).~~

Population denominators

We used ONS mid-year population estimates to derive population denominators.[20 21] These data are freely available online broken down by sex and year of age.

Analyses

We estimated the proportion of adolescents in the general population who had an emergency admission for injury between 10 and 19 years old. We~~,~~ ~~by~~ used~~ing~~ the number of adolescents in our derived retrospective cohort as the

For peer review only

numerator and ONS mid-year estimates for 10 year olds in 1998-2002 as the population denominator. We then calculated these proportions by types of injury at 10-19 years old (adversity-related [~~AdvRI~~adversity group and seven mutually exclusive subgroups] and accidents only [~~AccRI~~accidents ~~Only~~ only group]) and by age group, as described above.

We calculated the proportion of adolescents in the ~~AdvRI~~adversity group (and subgroups) and in the ~~AccRI~~accidents ~~Only~~ only group~~,~~ who had an emergency admission for injury twice and three or more times between 10 and 19 years old. We also calculated the proportions of adolescents with two or three or more admissions of any type (including non-emergency and non-injury).

We reported all results separately for girls and boys~~,~~ since differences between girls and boys have been reported for prevalence of adversity in the general population.[1 22-24] We calculated 95% confidence intervals for all proportions but did not present them here as they were all too narrow to convey any useful information (within one unit of the sample estimate). Analyses were carried out in StataSE 12.

For peer review only

For peer review only - http://bmjopen.bmj.com/site/about/guidelines.xhtml

RESULTS

There were 1 033 702 adolescents in HES admissions data in 1998-2011, of which 402 916 formed the study cohort (462 476 emergency admission for injury when considering multiple presentations from the same adolescent, 802 682 admissions of any type [including non-emergency and non-injury], 662 727 of which were overnight stays]) (Table 1), representing 12.4% (402 916/3 254 046) of the adolescent population. Twice as many boys as girls had an emergency admission for injury during adolescence (144 158/1 588 942 girls in the population [8.7%]; 258 503/1 665 104 boys [16.3%]).

For peer review only - http://bmjopen.bmj.com/site/about/guidelines.xhtml

Table 1. Characteristics of adolescents* between 10 and 19 years old whose entire ten years of adolescence (ages 10-19) occurred in 1998-2011.

	Adolescent Populationpopulation*	Adolescents with emergency admission(s) for injury between 10 and 19 years old, n (row %)			
**Characteristics**	**Total**	**Total**	**AdvRIAdversity**	**AccRIAccidents Onlyonly**	**Other Causescauses**
**All**	**3,254,046**	**402,916 (100.0)**	**141,248140,512 (35.1)(34.9)**	**233,907234,665 (58.1)(58.2)**	**27,76127,739 (6.9)(6.9)**
**Age***					
**Girls**	**1,588,942**	**144,158 (100.0)**	**72,80572,768 (50.5)(50.5)**	**59,52859,528 (41.3)(41.3)**	**11,88811,862 (8.2)(8.2)**
10-14 years	.	65,208 (100.0)	23,17823,289 (35.5)(35.7)	37,38837,303 (57.3)(57.2)	4,6424,616 (7.1)(7.1)
15-17 years	.	48,286 (100.0)	31,57331,620 (65.4)(65.5)	12,92212,882 (26.8)(26.7)	3,7913,784 (7.9)(7.8)
18-19 years	.	30,664 (100.0)	18,05417,859 (58.9)(58.2)	9,1559,343 (29.9)(30.5)	3,4553,462 (11.3)(11.3)
**Boys**	**1,665,104**	**258,503 (100.0)**	**68,40367,704 (26.5)(26.2)**	**174,267174,962 (67.4)(67.7)**	**15,83315,837 (6.1)(6.1)**
10-14 years	.	121,821 (100.0)	17,66717,660 (14.5)(14.5)	97,47897,511 (80.0)(80.0)	6,6766,650 (5.5)(5.5)
15-17 years	.	79,223 (100.0)	25,01425,004 (31.6)(31.6)	49,34549,366 (62.3)(62.3)	4,8644,853 (6.1)(6.1)
18-19 years	.	57,459 (100.0)	25,72225,040 (44.8)(43.6)	27,44428,085 (47.8)(48.9)	4,2934 [illegible]
Missing (Sex)	.	255 (100.0)	4040 (15.7)(15.7)	175175 (68.6)(68.6)	4040 (15.7)(15.7)
		Adolescents with emergency admission(s) for injury between 10 and 19 years old, n (column %)			
		**Total**	**AdvRI**	**AccRI Only**	**Other Causes**

Formatted Table

For peer review only - http://bmjopen.bmj.com/site/about/guidelines.xhtml

**All**	**3,254,046**	**402,916 (100.0)**	**140,512 (100.0)**	**234,665 (100.0)**	**27,739 (100.0)**
**Chronic Condition**	**-**	**117,190 (29.0)**	**47,001 (33.4)**	**52,509 (22.4)**	**16,706 (60.0)**

AdvRI = Adversity-related Injury, AccRI Only = Accident-related Injury Only.

*Adolescents whose entire ten years of adolescence (ages 10-19) occurred in 1998-2011.

*Based on ONS mid-year England statistics for 10 year olds in 1998-2002.[20]

**At first emergency admission for injury

For peer review only

Types of injury and age at emergency admission

One-third of the cohort (~~140 152~~141 248, 4.3% of the population) had a record of an emergency admission for adversity-related injury between 10 and 19 years old (the ~~AdvRI~~adversity group; 15~~3~~7 ~~633~~ 004 emergency admissions for adversity-related injury in total) (Table 1), with similar rates between sexes (72 ~~768~~805, 4.~~3~~6% girls in the population; ~~67 704~~68 403, 4.1% boys).

The remaining two-thirds of the cohort (26~~2~~1 ~~404,~~668, 8.1% of the adolescent population) had emergency admissions for injury which were never related to adversity (Table 1). Among these adolescents, 23~~4~~3 ~~665~~ 907 (89.4%) had an accident-related injury (the ~~AccRI~~accidents ~~Only~~ only group, 7.2% of the population) and 27 ~~739~~ 761 (10.6%) had no accident-related injury (~~Other~~ other ~~Causes~~ causes group, 0.9% of the population). A high~~er~~ proportion of the ~~Other~~ other ~~Causes~~ causes group were affected by a chronic condition[1] between 10 and 19 years old (~~16 706~~11 221/27 ~~739~~761, ~~60~~40.4%), compared with the ~~AdvRI~~adversity group (~~47 001/140 512~~45 321/141 248, 32.1~~3~~%) or ~~AccRI~~accidents ~~Only~~ only group (~~52 509~~49 434/~~234 665~~233 907, ~~22~~21.1%).

Proportions of adolescents in the general population and within individual age groups by ~~These groupings (AdvRI~~adversity [and subgroups], ~~AccRI~~accidents only, ~~Other~~ and other ~~Causes~~causes groups~~)~~ are provided in Supplementary Table 3.~~, as proportions of adolescents in the general population and within individual age groups.~~

[1] Defined by ICD-10 codes (Supplementary Table 2)

For peer review only

For peer review only - http://bmjopen.bmj.com/site/about/guidelines.xhtml

Types of adversity-related injury

Among adolescents in the ~~AdvRI~~adversity group (girls 72 ~~768~~805, boys ~~67,704~~68 403) (Figure 1), the most common type of adversity was drug or alcohol misuse (girls 91.5~~1~~%, boys ~~58~~60.0%). A higher proportion of boys than girls were exposed to ~~victimisation~~ violence (girls ~~17~~8.5%, boys ~~51.7~~47.6%), but a higher proportion of girls than boys were exposed to self-harm (girls 74.6%; boys ~~31.0~~32.9%).

For peer review only - http://bmjopen.bmj.com/site/about/guidelines.xhtml

Girls in the ~~AdvRI~~adversity group were most likely to be exposed to multiple types of adversity between 10 and 19 years old (69.2% + 2.0% + 1.2% + 0.2% = 72~~3~~.6%) (Figure 1), especially ~~SH+DA~~self-harm and drug or alcohol misuse (~~61.7~~69.2% of the entire adversity group, i.e., most of the 7~~3~~2.6%). Fewer boys in the ~~AdvRI~~adversity group were exposed to multiple types of adversity (~~36.2~~38.4%), the most common combination also being ~~SH+DA~~self-harm and drug or alcohol misuse (~~22.7~~24.8%).

For most of the adolescents who were exposed to multiple types of adversity, the combination of types was recorded at the same admission. For example, among the ~~352~~ 130 adolescent girls who were exposed to ~~victimisation~~ violence and self-harm between 10 and 19 years old (~~Figure 1~~Table 2), ~~85.3~~64.6% had both violence~~ctimisation~~ and self-harm codes present simultaneously in~~at~~ at least one emergency admission for injury (violence and drug or alcohol misuse~~V+DA 86.1~~78.8%, ~~SH+ DA~~self-harm and drug or alcohol misuse 99.7%, ~~V+ SH+ DA~~violence, self-harm and drug or alcohol misuse ~~83.2~~33.9%; boys: ~~V+SH~~violence and self-harm ~~63.2~~40.1%, violence and drug or alcohol misuse~~V+DA~~ 84.~~5~~1%, self-harm and drug or alcohol misuse~~SH+DA~~ 99.1~~0~~%, violence, self-harm and drug or alcohol misuse ~~V+SH+DA 59.1~~20.0%) (data not shown).

~~**Emergency readmissions for injury**~~

~~Adolescent girls in the AdvRI group (50.5% of all girls in the cohort) accounted for 59.9% of the total number of emergency admissions for injury coming from girls (data not shown), compared with girls in the AccRI Only group (41.3% of all girls) who accounted for 15.1%. Boys in the AdvRI group (26.2% of all~~

For peer review only - http://bmjopen.bmj.com/site/about/guidelines.xhtml

boys in the cohort) accounted for 29.6% compared with 61.7% contributed by boys in the AccRI Only group (67.7% of all boys).

More adolescents in the AdvRI group were readmitted for injury (i.e., had two or more emergency admissions for injury) between 10 and 19 years old (both girls and boys 17%) (Figure 2), than in the AccRI Only group (girls 5%; boys 7%). Among adolescents admitted for AdvRIs related to multiple types of adversity (Table 2), the proportion readmitted was even higher (multiple types: girls 21%, boys 24%; single type: girls 7%, boys 12%).

For peer review only - http://bmjopen.bmj.com/site/about/guidelines.xhtml

278 **Table 2.** Proportion of adolescents girls and boys with 1, 2 or 3+ emergency admission(s) for injury or 1, 2 or 3+ admission(s) of
279 any type, by types of adversity between 10 and 19 years old*

	Girls (%)							Boys (%)						
		Emergency admission(s) for injury			Admission(s) of any type				Emergency admission(s) for injury			Admission(s) of any type		
Adolescent group*	No. girls	1	2	3+	1	2	3+	No. boys	1	2	3+	1	2	3+
**All**	**144,158**	**88.688.6**	**8.38.3**	**3.13.1**	**57.657.6**	**20.520.5**	**22.022.0**	**258,503**	**90.390.3**	**8.08.0**	**1.81.8**	**68.568.5**	**18.718.7**	**12.912.9**
**AdvRIAdversity**	**72,80572,768**	**82.782.6**	**12.012.0**	**5.35.3**	**53.853.8**	**21.121.0**	**25.125.2**	**68,40367,704**	**83.583.4**	**12.412.5**	**4.14.1**	**64.864.7**	**19.419.4**	**15.815.9**
Any vViolence	6,21112,710	77.273.1	13.915.7	8.911.2	49.146.7	20.721.0	30.132.3	32,79935,003	83.282.6	12.812.9	4.04.5	65.664.9	19.619.4	14.815.7
Any sSelf-harmH	54,31554,315	79.379.3	13.913.9	6.86.8	51.251.2	21.521.5	27.327.3	21,08721,087	76.776.7	15.815.8	7.57.5	57.057.0	20.720.7	22.322.3
Any Ddrug or alcohol MmisuseA	66,64566,255	81.981.8	12.512.5	5.65.7	53.653.5	21.121.1	25.325.4	41,01439,264	81.180.9	13.613.7	5.35.4	62.962.6	19.519.6	17.617.8
**Single adversity**	**19,92419,194**	**92.892.9**	**6.26.1**	**1.01.0**	**61.261.2**	**19.819.6**	**19.019.2**	**43,56343,214**	**71.387.6**	**8.310.2**	**1.82.2**	**55.868.5**	**15.218.8**	**10.312.8**
Violence Onlyonly	3,7344,047	92.491.8	6.36.7	1.31.5	58.157.6	20.420.2	21.622.2	24,91225,908	87.187.0	10.610.7	2.22.3	68.368.1	19.219.2	12.612.7
Self-harmH Onlyonly	2,2962,114	90.390.8	8.17.7	1.61.5	54.454.4	20.720.8	24.924.8	2,2602,188	87.387.6	10.410.0	2.32.4	62.362.8	19.819.6	[illegible]
Drug or Aalcohol misuse Onlyonly	13,89413,033	93.393.5	5.95.7	0.80.8	63.263.4	19.519.3	17.417.3	16,39115,118	88.588.5	9.59.4	2.12.1	69.969.9	17.917.9	12.212.2

For peer review only - http://bmjopen.bmj.com/site/about/guidelines.xhtml

		Girls (%)							Boys (%)					
		Emergency admission(s) for injury			Admission(s) of any type				Emergency admission(s) for injury			Admission(s) of any type		
Adolescent group*	No. girls	1	2	3+	1	2	3+	No. boys	1	2	3+	1	2	3+
**Multiple adversity**	**52,88153,574**	**78.979.0**	**14.214.2**	**6.96.9**	**51.051.1**	**21.621.5**	**27.427.3**	**24,84024,490**	**77.376.1**	**16.716.5**	**7.57.5**	**59.058.0**	**21.020.7**	**21.521.4**
V + SH	130352	70.080.4	20.814.8	9.24.8	42.349.7	26.223.0	31.527.3	217344	41.671.8	15.421.8	6.16.4	32.853.8	16.024.4	14.221.8
V + DA	8621,373	81.984.6	15.113.0	3.02.3	52.254.0	22.922.4	24.923.6	6,0135,591	84.678.3	17.516.4	5.45.3	68.463.4	21.820.1	17.316.5
SH + DA	50,40444,911	80.181.8	13.713.1	6.35.1	51.852.9	21.621.6	26.625.5	16,95315,395	86.779.7	16.414.8	6.95.5	64.659.1	22.721.0	22.919.8
V + SH + DA	1,4856,938	36.859.5	31.621.5	31.519.0	25.538.7	19.921.2	54.540.2	1,6573,160	22.054.8	16.424.2	14.121.0	17.943.0	11.719.5	22.837.4
No adversity	71,35371,390	94.794.7	4.54.5	0.80.8	61.461.4	19.919.9	18.718.7	190,100190,799	92.492.7	6.36.4	0.90.9	69.569.8	18.318.4	11.811.8
**AccRIAccidents Onlyonly**	**59,46559,528**	**95.395.3**	**4.14.1**	**0.60.6**	**66.566.6**	**18.718.7**	**14.714.7**	**174,267174,962**	**92.392.6**	**6.56.5**	**0.90.9**	**71.171.4**	**18.018.1**	**10.510.5**
**Other Causescauses**	**11,88811,862**	**91.591.6**	**6.36.3**	**2.12.1**	**35.535.4**	**25.825.8**	**38.838.7**	**15,83315,837**	**93.493.4**	**5.05.0**	**1.61.6**	**51.551.7**	**22.122.1**	**26.326.2**

V = VictimisationViolence, SH = Self-harm, DA = Drug or Alcohol Misuse, AdvRI = Adversity-related Injury, AccRI Only = Accident-related Injury Only.
* Each adolescent classified by all adversitiesadversity/accidents seen at any emergency admission(s) for injury between 10 and 19 years old.

For peer review only

For peer review only - http://bmjopen.bmj.com/site/about/guidelines.xhtml

Emergency re-admissions for injury

Adolescent girls in the adversity group (50.5% of all girls in the cohort) accounted for 50.0% of the total number of emergency admissions for injury coming from girls (data not shown), compared with girls in the accidents only group (41.3% of all girls) who accounted for 36.6%. Boys in the adversity group (26.2% of all boys in the cohort) accounted for 29.1% compared with 65.0% contributed by boys in the accidents only group (67.7% of all boys).

More adolescents in the adversity group were re-admitted for injury (i.e., had two or more emergency admissions for injury) between 10 and 19 years old (girls 17.3%, boys 16.5%) (Figure 2), than in the accidents only group (girls 4.7%; boys 7.4%). Among adolescents admitted for injuries related to multiple types of adversity (Table 2), the proportion re-admitted was even higher (multiple types: girls 21.1%, boys 24.2%; single type: girls 7.2%, boys 10.1%).

Similarly, a higher proportion of adolescents in the AdvRIadversity group had two more admissions of any type (including non-emergency and non-injury) between 10 and 19 years old (girls 436.2%; boys 35.23%); (Table 2) compared with adolescents in the AccRIaccidents Only only group (girls 33.4%; boys 28.56%). This proportion was even higher for adolescents in the AdvRIadversity group who were admitted with multiple types of adversity (multiple types: girls 48.849.0%, boys 42.51%; single type: girls 38.8%, boys 31.625.5%).

For peer review only - http://bmjopen.bmj.com/site/about/guidelines.xhtml

DISCUSSION

More than one in 20 adolescents in England had at least one emergency admission for adversity-related injury between 10 and 19 years old. These adolescents accounted for a third of all adolescents with emergency admissions for injury and for a disproportionate number of re-admissions for injury, particularly adolescents admitted with multiple types of adversity-related injury. Targeting adolescents admitted with adversity-related injury could reduce their risk of future harm, ~~and~~ the rate of re-admissions to hospital, and ~~, which~~ ~~could reduce~~ health care ~~some of the most expensive types of healthcare and the UK government is keen to reduce~~ costs .[25]

Longitudinally-linked admissions allowed us to study the entire ten years of adolescence in 402 916 ~~adolescents~~individuals. We were able, to distinguish between types of adversity that co-occurred during adolescence or at the same admission, and to study re-admissions.

One weakness of this study was our reliance on diagnostic codes recorded in administrative data. Violence by carers, which could be coded under maltreatment, ~~Maltreatment~~ and drug or alcohol misuse have been shown to be under-recorded using ICD-10,[26 27] but false positives are rare.[15] To address under-recording, we used what we considered to be sensitive clusters of codes for adversity. Other factors related to recording or coding practices,[12 14 15 27 28] for example, new guidelines for defining maltreatment,[14] can also affect ascertainment. Because of the relative insensitivity but good specificity of the coding clusters, some adolescents who were classified in the ~~AccRI~~accidents only group may in fact belong to the ~~AdvRI~~adversity group, but did not have their adversity recognised or recorded.

For peer review only

For peer review only - http://bmjopen.bmj.com/site/about/guidelines.xhtml

Consequently, our prevalence estimates of admission for different types of adversity-related injury are likely to provide a lower bound for the true prevalence.

Our prevalence estimates of admission for injury related to individual types of adversity from the general adolescent population are consistent with previous reports for emergency admissions for assault-related injury in 2004-2009 and for all admissions (emergency and non-emergency) for self-harm and drug or alcohol misuse.[1 2 24 29] Previous studies have reported higher rates of drug or alcohol misuse in boys than in girls in the general adolescent population.[24] We found higher rates in girls. This difference could indicate that girls exposed to drug or alcohol misuse are more likely to be injured,~~,~~ to present to hospital, or to be admitted after a hospital presentation, than boys ~~be admitted after presenting to hospital, as a result~~.

Our estimated rates of re-admission of any type (including non-emergency and non-injury) for ~~victimisation~~ violence (girls ~~53.3~~50.8%, boys ~~35.1~~34.4%) (Table 2) and self-harm (girls 48.8%, boys 43.0%) were higher than previously reported (11% for ~~victimisation~~violence, 33% for self-harm).[3 4] These discrepancies are likely to be because we considered the whole ten years of adolescence and re-admission of any type, whereas previous studies looked at re-attendance up to the following year and for the same type of adversity-related injury.[3 4]

The results of this study should inform policy initiatives and national guidelines. Firstly, a substantial proportion of adolescents are affected by adversity and they account for a large proportion (29.~~6~~ 1 to -50~~9~~.0~~9~~%) of all emergency admissions to hospital for injury in this age group. Secondly, we

For peer review only - http://bmjopen.bmj.com/site/about/guidelines.xhtml

show the large burden of injury admission for all three types of adversity, yet there are currently no national clinical guidelines for managing cases of ~~assault~~violence, other than responding to violence by caregivers.[30] Finally, these results show that adolescents often present with multiple types of adversity (especially in girls), even though guidelines exist only for managing individual problems.[31-33]

In addition, policy makers need to be aware of the widely varying aetiological pathways to admission with adversity-related injury. Our approach to defining this group of adolescents is not designed to reflect the complexity or severity of these cases. For example, admission for multiple types of adversity-related injury is a poor proxy indicator of severity. Effective interventions will need to be tailored to the individual~~,~~ based on specialist clinical assessment. However, all three types of adversity are likely to reflect a combination of underlying psychosocial need and environmental and social stressors.[34] ~~This intervention~~

Hospital interventions may reduce the risk of future harm, including the incidence of other types of harm not seen in hospital, e.g., further adversity-related injury not leading to admission~~, that are likely to be more frequent in these adolescents, and which the hospital administrative data we used does not capture~~. Further research using ~~work in admissions~~ linked data ~~linked~~ from ~~to other administrative datasets, e.~~health care sectors such as~~g.,~~ accident and emergency ~~data~~, ~~could tell us if the risk of these other types of harm is also increased in adolescents admitted with adversity-related injury~~could shed light on the overall burden of adversity-related injury on hospitals~~.this, as well as.~~ ~~tell us whether the risk of future harm is increased in~~

For peer review only - http://bmjopen.bmj.com/site/about/guidelines.xhtml

~~adolescents who attend hospital with adversity-related injury but are not admitted~~. Such analyses are limited by the quality of ~~Such work in England would be challenging, as~~ accident and emergency data in England (available since 2007) and the resulting problems with identifying reasons for presentation and accurately linking individuals to long-term outcomes.~~are much sparser than national admissions data and have only been collected since 2007~~[35] However, ~~Existing~~ longitudinally-linked datasets in other countries could provide insights into long-term outcomes for this vulnerable group of adolescents.

For peer review only

For peer review only - http://bmjopen.bmj.com/site/about/guidelines.xhtml

Contributors

Annie Herbert – helped to conceive and design the study, analysed and interpreted the data, drafted the article, revised it critically for important intellectual content and approved the final version to be published.

Leah Li – helped to conceive and design the study, interpreted the data, revised the article critically for important intellectual content and approved the final version to be published.

Arturo González-Izquierdo – helped to conceive and design the study, acquired and interpreted the data, revised the article critically for important intellectual content and approved the final version to be published.

Ruth Gilbert – helped to conceive and design the study, acquired and interpreted the data, revised the article critically for important intellectual content and approved the final version to be published.

Competing interests None.

Funding AH was supported by the Policy Research Unit in the Health of Children, Young People and Families, which is funded by the Department of Health Policy Research Programme (grant reference number: 109/0001). This report is an independent research commissioned by the Department of Health. The views expressed in this publication are those of the authors and not necessarily those of the Department of Health. AH is also supported by the University College London IMPACT studentship. RG is supported by awards establishing the Farr Institute of Health Informatics Research at University College London Partners from the Medical Research Council and a

For peer review only

For peer review only - http://bmjopen.bmj.com/site/about/guidelines.xhtml

consortium of funders (MR/K006584/1). The study funders played no part in the design, data analysis and interpretation of this study; the writing of the manuscript or the decision to submit the paper for publication, and the authors' work was independent of their funders.

Ethics Both Hospital Episode Statistics (HES) admissions data and Office for National Statistics (ONS) mid-year population estimates are derived from routinely collected administrative data. HES data were pseudonymised before we received them and therefore we did not require Research Ethics Committee approval.

Data sharing No additional data on HES are available. ONS mid-year population estimates may be accessed freely online: http://www.ons.gov.uk/ons/publications/all-releases.html?definition=tcm%3A77-22371.

For peer review only

REFERENCES

1. Bellis MA, Hughes K, Wood S, et al. National five-year examination of inequalities and trends in emergency hospital admission for violence across England. Injury prevention : journal of the International Society for Child and Adolescent Injury Prevention 2011;**17**(5):319-25 doi: 10.1136/ip.2010.030486[published Online First: Epub Date]|.
2. Hawton K, Rodham K, Evans E, et al. Deliberate self harm in adolescents: self report survey in schools in England. Bmj 2002;**325**(7374):1207-11
3. Meuleners LB, Lee AH, Hendrie D. Interpersonal violence hospitalisations for adolescents: A population-based study. Journal of paediatrics and child health 2010;**46**(11):686-90 doi: 10.1111/j.1440-1754.2010.01818.x[published Online First: Epub Date]|.
4. Lilley R, Owens D, Horrocks J, et al. Hospital care and repetition following self-harm: multicentre comparison of self-poisoning and self-injury. The British Journal of Psychiatry 2008;**192**(6):440-45
5. Snider C, Lee J. Youth violence secondary prevention initiatives in emergency departments: a systematic review. Cjem 2009;**11**(2):161-8
6. Ougrin D, Boege I, Stahl D, et al. Randomised controlled trial of therapeutic assessment versus usual assessment in adolescents with self-harm: 2-year follow-up. Archives of disease in childhood 2013;**98**(10):772-6 doi: 10.1136/archdischild-2012-303200[published Online First: Epub Date]|.
7. Crawford MJ, Patton R, Touquet R, et al. Screening and referral for brief intervention of alcohol-misusing patients in an emergency department: a pragmatic randomised controlled trial. The Lancet;**364**(9442):1334-39 doi: http://dx.doi.org/10.1016/S0140-6736(04)17190-0[published Online First: Epub Date]|.
8. Johnson SB, Bradshaw CP, Wright JL, et al. Characterizing the teachable moment: is an emergency department visit a teachable moment for intervention among assault-injured youth and their parents? Pediatric emergency care 2007;**23**(8):553-59
9. Inpatient HES Data Dictionary. Leeds: Health and Social Care Information Centre, 2010.

Formatted: Font: Cambria, 14 pt, French (France), Do not check spelling or grammar

For peer review only - http://bmjopen.bmj.com/site/about/guidelines.xhtml

10. World Health Organisation. Adolescent Health. Secondary Adolescent Health 2013. http://www.who.int/topics/adolescent_health/en/.
11. National Health Service Act, 2006.
12. Gonzalez-Izquierdo A, Woodman J, Copley L, et al. Variation in recording of child maltreatment in administrative records of hospital admissions for injury in England, 1997-2009. Archives of disease in childhood 2010;**95**(11):918-25 doi: 10.1136/adc.2009.180216[published Online First: Epub Date]|.
13. International Statistical Classification of Diseases and Related Health Problems 10th Revision. Secondary International Statistical Classification of Diseases and Related Health Problems 10th Revision. http://apps.who.int/classifications/icd10/browse/2010/en.
14. Saperia J, Lakhanpaul M, Kemp A, et al. When to suspect child maltreatment: summary of NICE guidance. Bmj 2009;**339**:b2689 doi: 10.1136/bmj.b2689[published Online First: Epub Date]|.
15. Gonzalez-Izquierdo A, Ward A, M OD, et al. Cross-country comparison of victimisation-related injury admission in children and adolescents in England and Western Australia. BMC Health Serv Res 2013;**13**(1):260 doi: 10.1186/1472-6963-13-260[published Online First: Epub Date]|.
16. Patton GC, Viner R. Pubertal transitions in health. Lancet 2007;**369**(9567):1130-9 doi: 10.1016/S0140-6736(07)60366-3[published Online First: Epub Date]|.
17. Gill T. How old are GCSE candidates? Statistics Report Series, 2010.
18. Directgov. AS and A levels. Secondary AS and A levels 2012. http://webarchive.nationalarchives.gov.uk/20121015000000/http://www.direct.gov.uk/en/EducationAndLearning/QualificationsExplained/DG_10039018.
19. Buying Alcohol. Secondary Buying Alcohol 2013. https://www.drinkaware.co.uk/check-the-facts/alcohol-and-the-law/buying-alcohol.
20. All releases of Population Estimates for UK, England and Wales, Scotland and Northern Ireland: Office for National Statistics, 2013.
21. Office for National Statistics. Mid-year population estimates short methods guide: Office for National Statistics, 2010.
22. Radford L. Child abuse and neglect in the UK today. NSPCC Research: NSPCC, 2011.

For peer review only - http://bmjopen.bmj.com/site/about/guidelines.xhtml

23. *Truth hurts: report of the national inquiry into self-harm among young people*. London: Mental Health Foundation, 2006.
24. Hibell B, Guttormsson U, Ahlström S, et al. The 2011 ESPAD Report. Substance use among students in 2012;**36**
25. National Audit Office. Emergency admissions to hospital: managing the demand, 2013.
26. Scott D, Tonmyr L, Fraser J, et al. The utility and challenges of using ICD codes in child maltreatment research: A review of existing literature. Child abuse & neglect 2009;**33**(11):791-808 doi: S0145-2134(09)00202-6 [pii]
10.1016/j.chiabu.2009.08.005[published Online First: Epub Date]|.
27. McKenzie K, Harrison JE, McClure RJ. Identification of alcohol involvement in injury-related hospitalisations using routine data compared to medical record review. Australian and New Zealand journal of public health 2010;**34**(2):146-52 doi: 10.1111/j.1753-6405.2010.00499.x[published Online First: Epub Date]|.
28. Farrar S, Yi D, Sutton M, et al. Has payment by results affected the way that English hospitals provide care? Difference-in-differences analysis. Bmj 2009;**339** doi: 10.1136/bmj.b3047[published Online First: Epub Date]|.
29. Hawton K, Rodham K, Evans E, et al. Adolescents Who Self Harm: A Comparison of Those Who Go to Hospital and Those Who Do Not. Child Adol Ment H-Uk 2009;**14**(1):24-30 doi: DOI 10.1111/j.1475-3588.2008.00485.x[published Online First: Epub Date]|.
30. What to do if you're worried that a child is being abused. In: Skills DfE, ed.: DfES Publications, 2006.
31. *Self-harm. The short term physical and psychological management and secondary prevention of self-harm in primary and secondary care.* London: National Institute for Health and Clinical Excellence, 2011.
32. *Drug misuse: Psychosocial interventions*. London: National Institute for Health and Clinical Excellence, 2007.
33. Alcohol-use disorder : diagnosis, assessment and management of harmful drinking and alcohol dependence. London: National Institute for Health and Care Excellence, 2011.
34. Viner RM, Ozer EM, Denny S, et al. Adolescence and the social determinants of health. Lancet 2012;**379**(9826):1641-52 doi: 10.1016/S0140-6736(12)60149-4[published Online First: Epub Date]|.

Formatted: Font: Cambria, 14 pt, French (France), Do not check spelling or grammar

Formatted: Font: Cambria, 14 pt, French (France), Do not check spelling or grammar

For peer review only - http://bmjopen.bmj.com/site/about/guidelines.xhtml

35. Hospital Episode Statistics Accident & Emergency (England), 2007-. Secondary Hospital Episode Statistics Accident & Emergency (England), 2007-. http://adrn.ac.uk/catalogue/cataloguepage?sn=888040.

For peer review only

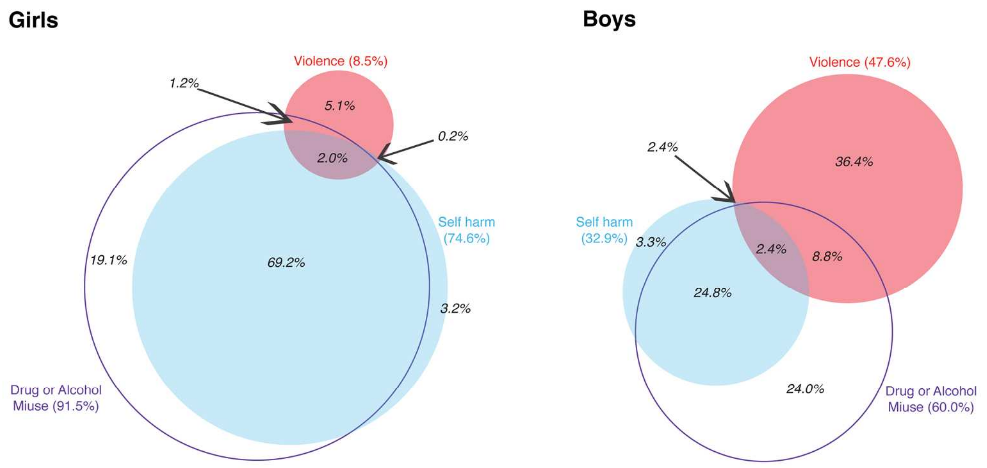

Number (%) of adolescents with adversity-related injury, by types of adversity between 10 and 19 years old* and sex

*Each adolescent classified by all adversity recorded at any emergency admission(s) for injury between10 and 19 years old

173x86mm (300 x 300 DPI)

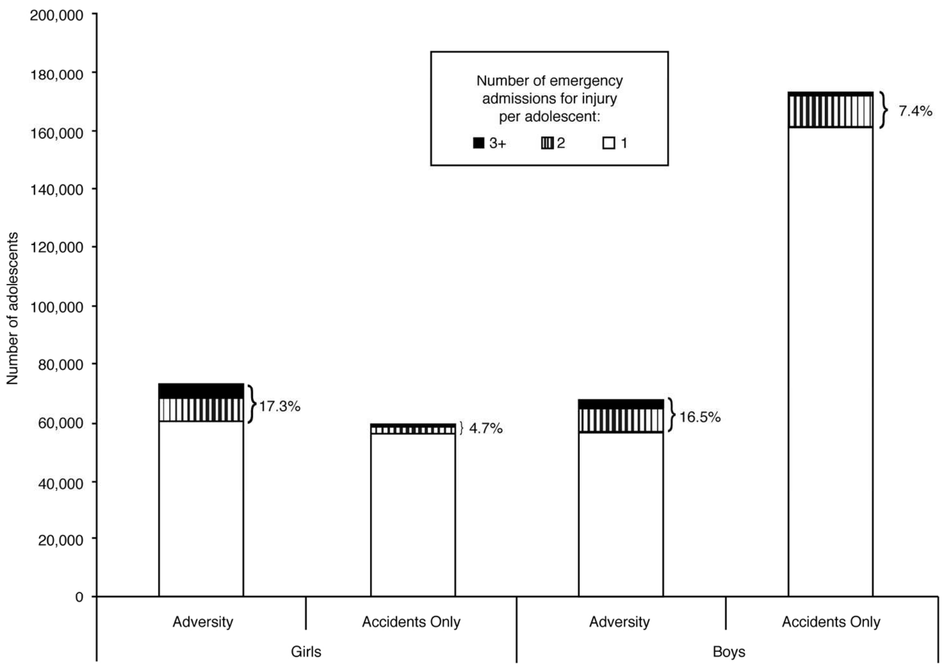

Number of adolescents with 1, 2 and 3 or more emergency admission(s) for injury between 10 and 19 years old*, by types of injury between 10 and 19 years old** and sex

*Percentages are of adolescents who have 2 or more emergency admissions for injury between 10 and 19 years old

**Each adolescent was classified by all adversity/accidents recorded at any emergency admission(s) for injury
173x125mm (300 x 300 DPI)

only

Supplementary Tables

Supplementary Table 1. Cohort (shaded) who have at least one emergency admission for injury between 10 and 19 years old in 1998-2011 (N = 402,916)

1998	*1999*	*2000*	*2001*	*2002*	…	*2007*	*2008*	*2009*	*2010*	*2011*
6	7	8	9	10	…	15	16	17	18	19
7	8	9	10	11	…	16	17	18	19	20
8	9	10	11	12	…	17	18	19	20	21
9	10	11	12	13	…	18	19	20	21	22
10	11	12	13	14	…	19	20	21	22	23

For peer review only

Supplementary Table 2. Definitions and International Classification of Diseases (ICD-10) diagnostic codes used to classify emergency admissions for injury, chronic conditions and types of adversity*

ADMISSIONS AND ADMISSION TYPES

Admission

Admissions were defined by linked episodes of inpatient care, including day cases (any case > 4 hours stay). Any admission which occurred within one day or was a hospital transfer from a previous admission was considered to be the same admission. An episode may have up to 20 diagnosis codes entered, but 88% of admissions in this cohort had episodes with four or less diagnoses recorded (data not shown).

Emergency (acute; unplanned) admission

An admission was considered to be an emergency if the method of admission variable (‘admimeth’) was 21-24 or 28.(1)

Injury

An admission was considered to be for injury if any of the diagnosis codes in the first episode of the admission were S or T codes (Chapter XIX of the ICD -10).

CHRONIC CONDITIONS

ICD-10 codes as per Hardelid et al, 2013,(2) excluding E24.4, F10-F19, G31.2, G40.5, G62.1, G72.1, K29.2, K70, K85.2, K86.0, O35.4, R78.1-R78.5, Z50.2, Z50.3, Z86.4, X64-X84, Y10-Y34, Z91.5, F30.0-F30.2, F30.8-F32.3, F32.8-F334, F33.8-F34.1, F34.8-F34.9, F38.0-F38.1, F38.8, F39-F40.2, F40.8- F41.3, F41.8-F41.9.

ADVERSITY CLUSTERS AND DESCRIPTIONS*	ICD-10 CODES
**Violence**	
Maltreatment	
Maltreatment syndromes	T74
Effects of other deprivation (extreme neglect)	T73
Perpetrator of neglect and other maltreatment syndromes	Y06, Y07
Assault	
Assault by bodily force and sexual assault	Y04, Y05
Other types of assault	X85 - Y03, Y08 - Y09
Undetermined cause	
Events of undetermined intent	Y20 - Y34
Examination and observation following other inflicted injury	Z04.5
Examination and observation for other reasons: request for expert evidence	Z04.8
**Self-harm**	
Intentional self-poisoning by and exposure to…	
…drugs	X60-X63
…other and unspecified drugs, medicaments and biological substances	X64
…alcohol	X65
…organic solvents and halogenated hydrocarbons and their vapours	X66
…other gases and vapours	X67
…pesticides	X68
…other and unspecified chemicals and noxious	X69

For peer review only

substances	
Intentional self-harm by…	
…hanging, strangulation and suffocation	X70
…drowning and submersion	X71
…firearm discharge	X72-X74
…explosive material	X75
…smoke, fire and flames, or steam, hot vapours and hot objects	X76-X77
…sharp/blunt objects	X78-X79
...jumping from a high place	X80
…jumping or lying before a moving object, or crashing a motor vehicle	X81-82
…other specified means	X83
…unspecified means	X84
Personal history of self-harm	Z91.5
**Drug or alcohol misuse**	
Drugs, medicaments and biological substances (illicit drugs)	
Mental and behavioural disorders due to psychoactive substance use	F11 - F17, F19
Finding of drugs not normally found in blood	R78.1 - R78.5
Poisoning by drugs, medicaments and biological substances	T36 - T50 (not including T50.6)
Poisoning, undetermined intent	Y10 - Y14
Drug rehabilitation	Z50.3
Drug abuse counselling and surveillance	Z71.5
Drug use	Z72.2
Environmental/ Domestic substances	
Mental and behavioural disorders due to use of volatile solvents	F18
Accidental poisoning by and exposure to noxious substances	X40 – X44, X46 - X49
Intentional self-poisoning by and exposure to noxious substances	X69
Poisoning by chemical or noxious substance, undetermined intent	Y16 - Y19
Codes mentioning both alcohol and drugs	
Special epileptic syndromes - (related to alcohol, drugs, etc.)	G40.5
Blood-alcohol and blood-drug test	Z04.0
Alcohol	
Alcohol-induced pseudo-Cushing's syndrome	E24.4
Mental and behavioural disorders due to use of alcohol	F10
Degeneration of nervous system due to alcohol	G31.2
Alcoholic polyneuropathy	G62.1
Alcoholic myopathy	G72.1
Alcoholic cardiomyopathy	I42.1
Alcoholic gastritis	K29.2
Alcoholic liver disease	K70
Alcohol-induced acute pancreatitis	K85.2

For peer review only

Alcohol-induced chronic pancreatitis	K86.0
Maternal care for (suspected) damage to fetus from alcohol	O35.4
Finding of alcohol in blood	R78.0
Poisoning: antidotes and chelating agents, not elsewhere classified	T50.6
Toxic effect of alcohol	T51
Accidental poisoning by exposure to alcohol	X45
Poisoning by exposure to alcohol, undetermined intent	Y15
Evidence of alcohol involvement determined by blood alcohol level	Y90
Evidence of alcohol involvement determined by level of intoxication	Y91
Alcohol rehabilitation	Z50.2
Alcohol abuse counselling and surveillance	Z71.4
Alcohol use	Z72.1
**Accidents**	
Transport accidents	V01 - V99
Falls	W00 - W19
Exposure to inanimate mechanical forces	W20 - W49
Exposure to animate mechanical forces	W50 - W64
Accidental drowning and submersion	W65 - W74
Other accidental threats to breathing	W75 - W84
Exposure to electric current, radiation and extreme ambient air temperature and pressure	W85 - W99
Exposure to smoke, fire and flames	X00 - X09
Contact with heat and hot substances	X10 - X19
Contact with venomous animals and plants	X20 - X29
Exposure to forces of nature	X30 - X39
Accidental poisoning by and exposure to noxious substances	X40 - X49
Overexertion, travel and privation	X50 - X57
Accidental exposure to other and unspecified factors	X58 - X59

For peer review only

Supplementary Table 3. Population prevalence* (per 100,000) of emergency admission(s) for injury, by types of adversity within age periods** and sex

	All girls	All boys	Girls			Boys		
**Adolescent group****	**10-19y**	**10-19y**	**10-14y**	**15-17y**	**18-19y**	**10-14y**	**15-17y**	**18-19y**
All	9,073	16,333‡	4,104	3,260§	2,189§	7,316‡	5,069‡§	3,852‡§
**Adversity**	**4,582**	**4,108‡**	**1,240**	**2,124§**	**1,329§**	**669‡**	**1,458‡§**	**1,748‡§**
Any violence	391	1,970‡	103	152§	110	277‡	731‡§	833‡§
Any self-harm	3,418	1,266‡	777	1,463§	923§	170‡	393‡§	478‡§
Any drug or alcohol misuse	4,194	2,463‡	1,112	1,959§	1,221§	375‡	807‡§	1,077‡§
**Single adversity**	**1,254**	**2616‡**	**495**	**687§**	**412§**	**516**	**991‡§**	**1,114‡§**
Violence only	235	1,496‡	77	99§	75	256‡	609‡§	635‡§
Self-harm only	144	136	48	63§	31§	37‡	39	34
Drug or alcohol misuse only	874	984‡	369	525§	305§	224‡	342‡§	445‡§
**Multiple adversity**	**3,328**	**1,492‡**	**745**	**1,436§**	**917§**	**153‡**	**467‡§**	**634‡§**
V+SH	8	13‡	3	3	1§	1‡	2	2
V+DA	54	361‡	17	37§	26§	19	114‡§	190‡§
SH+DA	3,172	1,018‡	720	1,384§	882§	131‡	345‡§	436‡§
V+SH+DA	93	100‡	6	13§	8	2‡	5‡§	6§
No adversity	4,491	11,417‡	2,676	1,136§	860§	6,648‡	3,611‡	2,171‡§
**Accidents only**	**748**	**10,466‡**	**2,221**	**804§**	**580§**	**5,887‡**	**3,109‡**	**1,784‡§**
**Other causes**	**3,742**	**951‡**	**455**	**332§**	**279§**	**761‡**	**503‡**	**388‡§**

For peer review only

V = Violence, SH = Self-harm, DA = Drug or Alcohol Misuse
*Due to large denominators, confidence intervals for population prevalence were too narrow to provide any meaningful interpretation and so are not shown.
**Each adolescent classified by all adversity/accidents seen at any emergency injury admission(s) at 10-19 years, 10-14 years, 15-17 years and 18-19 years, respectively.
‡Statistically significantly different from girls of the same age (according to 95% confidence intervals).
§Statistically significantly different from 10-14 year olds of the same sex (according to 95% confidence intervals).

For peer review only

REFERENCES

1.Inpatient HES Data Dictionary. Leeds: Health and Social Care Information Centre, 2010.
2.Hardelid P DN, Davey J, Primbramska I, Gilbert R. Overview of child deaths in the four UK countries. London: 2013.

For peer review only

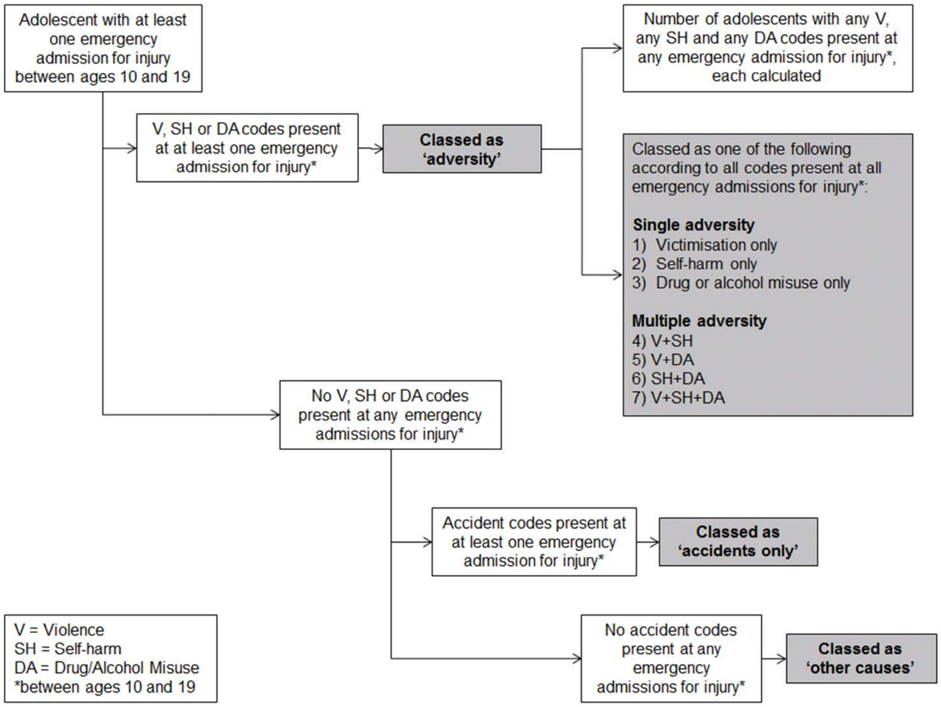

Classification of groups of adolescents

*between ages 10 and 19

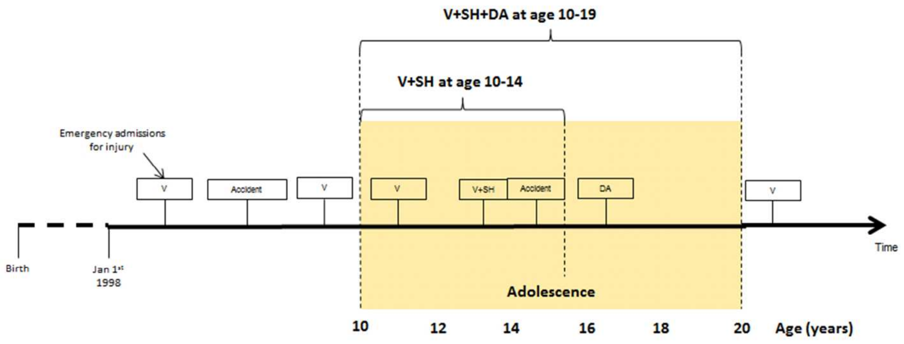

Supplementary Figure 2. Example of classification of groups of adolescents within age groups and across adolescence (ages 10-19).

V = victimisation, SH = self-harm, DA = drug or alcohol misuse.
254x102mm (96 x 96 DPI)

For peer review only

Supplementary Tables

Supplementary Table 1. Cohort (shaded) who have at least one emergency admission for injury between 10 and 19 years old in 1998-2011 (N = 402,916)

1998	*1999*	*2000*	*2001*	*2002*	…	*2007*	*2008*	*2009*	*2010*	*2011*
6	7	8	9	10	…	15	16	17	18	19
7	8	9	10	11	…	16	17	18	19	20
8	9	10	11	12	…	17	18	19	20	21
9	10	11	12	13	…	18	19	20	21	22
10	11	12	13	14	…	19	20	21	22	23

For peer review only

Supplementary Table 2. Definitions and International Classification of Diseases (ICD-10) diagnostic codes used to classify emergency admissions for injury, chronic conditions and types of adversity*

ADMISSIONS AND ADMISSION TYPES

Admission

Admissions were defined by linked episodes of inpatient care, including day cases (any case > 4 hours stay). Any admission which occurred within one day or was a hospital transfer from a previous admission was considered to be the same admission. An episode may have up to 20 diagnosis codes entered, but 88% of admissions in this cohort had episodes with four or less diagnoses recorded (data not shown).

Emergency (acute; unplanned) admission

An admission was considered to be an emergency if the method of admission variable (‘admimeth’) was 21-24 or 28.(1)

Injury

An admission was considered to be for injury if any of the diagnosis codes in the first episode of the admission were S or T codes (Chapter XIX of the ICD -10).

CHRONIC CONDITIONS

ICD-10 codes as per Hardelid et al, 2013,(2) excluding E24.4, F10-F19, G31.2, G40.5, G62.1, G72.1, K29.2, K70, K85.2, K86.0, O35.4, R78.1-R78.5, Z50.2, Z50.3, Z86.4, X64-X84, Y10-Y34, Z91.5, F30.0-F30.2, F30.8-F32.3, F32.8-F334, F33.8-F34.1, F34.8-F34.9, F38.0-F38.1, F38.8, F39-F40.2, F40.8- F41.3, F41.8-F41.9.

ADVERSITY CLUSTERS AND DESCRIPTIONS*	ICD-10 CODES
**~~Victimisation~~Violence**	
Maltreatment	
Maltreatment syndromes	T74
Effects of other deprivation (extreme neglect)	T73
Perpetrator of neglect and other maltreatment syndromes	Y06, Y07
Assault	
Assault by bodily force and sexual assault	Y04, Y05
Other types of assault	X85 - Y03, Y08 - Y09
Undetermined cause	
Events of undetermined intent	Y20 - Y34
Examination and observation following other inflicted injury	Z04.5
Examination and observation for other reasons: request for expert evidence	Z04.8
~~*Adverse social circumstances*~~	
~~Neonatal withdrawal symptoms from maternal use of drugs of addiction~~	~~P96.1~~
~~Other problems related to physical environment~~	~~Z58.8~~
~~Problem related to physical environment, unspecified~~	~~Z58.9~~
~~Homelessness~~	~~Z59.0~~
~~Inadequate housing~~	~~Z59.1~~
~~Lack of adequate food~~	~~Z59.4~~
~~Extreme poverty~~	~~Z59.5~~
~~Insufficient social insurance and welfare support~~	~~Z59.7~~
~~Problem related to housing and economic circumstances, unspecified~~	~~Z59.9~~

For peer review only

~~Problems related to social environment~~	~~Z60~~
~~Problems related to negative life events in childhood~~	~~Z61~~
~~Other problems related to upbringing~~	~~Z62~~
~~Other problems related to primary support group~~	~~Z63~~
~~Discord with counsellors~~	~~Z64.4~~
~~Problems related to other legal circumstances~~	~~Z65.3~~
~~Other specified problems related to psychosocial circumstances~~	~~Z65.8~~
~~Problem related to unspecified psychosocial circumstances~~	~~Z65.9~~
~~Problems related to lifestyle~~	~~Z72.3 - Z72.9~~
~~Problems related to care-provider dependency~~	~~Z74~~
~~Health supervision and care of foundling~~	~~Z76.1~~
~~Health supervision and care of other healthy infant and child~~	~~Z76.2~~
~~Family history of mental and behavioural disorders~~	~~Z81~~
~~Personal history of other specified risk-factors, not elsewhere classified~~	~~Z91.8~~

Self-harm

Intentional self-poisoning by and exposure to…	
…drugs	X60-X63
…other and unspecified drugs, medicaments and biological substances	X64
…alcohol	X65
…organic solvents and halogenated hydrocarbons and their vapours	X66
…other gases and vapours	X67
…pesticides	X68
…other and unspecified chemicals and noxious substances	X69
Intentional self-harm by…	
…hanging, strangulation and suffocation	X70
…drowning and submersion	X71
…firearm discharge	X72-X74
…explosive material	X75
…smoke, fire and flames, or steam, hot vapours and hot objects	X76-X77
…sharp/blunt objects	X78-X79
...jumping from a high place	X80
…jumping or lying before a moving object, or crashing a motor vehicle	X81-82
…other specified means	X83
…unspecified means	X84
Personal history of self-harm	Z91.5

~~Alcohol/d~~Drug or alcohol misuse

~~*Alcohol*~~	
~~Alcohol-induced pseudo-Cushing's syndrome~~	~~E24.4~~
~~Mental and behavioural disorders due to use of alcohol~~	~~F10~~

For peer review only

~~Degeneration of nervous system due to alcohol~~	~~G31.2~~
~~Alcoholic polyneuropathy~~	~~G62.1~~
~~Alcoholic myopathy~~	~~G72.1~~
~~Alcoholic cardiomyopathy~~	~~I42.1~~
~~Alcoholic gastritis~~	~~K29.2~~
~~Alcoholic liver disease~~	~~K70~~
~~Alcohol-induced acute pancreatitis~~	~~K85.2~~
~~Alcohol-induced chronic pancreatitis~~	~~K86.0~~
~~Maternal care for (suspected) damage to fetus from alcohol~~	~~O35.4~~
~~Fetus and newborn affected by maternal use of alcohol[‡]~~	~~P04.3~~
~~Fetal alcohol syndrome (dysmorphic)[‡]~~	~~Q86.0~~
~~Finding of alcohol in blood~~	~~R78.0~~
~~Poisoning: antidotes and chelating agents, not elsewhere classsified~~	~~T50.6~~
~~Toxic effect of alcohol~~	~~T51~~
~~Accidental poisoning by exposure to alcohol~~	~~X45~~
~~Intentional self-poisoning by and exposure to alcohol**~~	~~X65~~
~~Poisoning by exposure to alcohol, undetermined intent~~	~~Y15~~
~~Evidence of alcohol involvement determined by blood alcohol level~~	~~Y90~~
~~Evidence of alcohol involvement determined by level of intoxication[‡]~~	~~Y91~~
~~Alcohol rehabilitation~~	~~Z50.2~~
~~Alcohol abuse counselling and surveillance~~	~~Z71.4~~
~~Alcohol use‡~~	~~Z72.1~~
Drugs, medicaments and biological substances (illicit drugs)	
Mental and behavioural disorders due to psychoactive substance use	F11 - F17, F19
~~Maternal care for (suspected) damage to fetus by drugs[‡]~~	~~O35.5~~
~~Neonatal jaundice due to drugs or toxins transmitted from mother or given to newborn[‡]~~	~~P58.4~~
~~Neonatal withdrawal symptoms from maternal use of drugs of addiction[‡]~~	~~P96.1~~
Finding of drugs not normally found in blood	R78.1 - R78.5
Poisoning by drugs, medicaments and biological substances	T36 - T50 (not inc~~.~~luding T50.6)
~~Intentional self-poisoning by and exposure to antiepileptic, sedative-hypnotic, antiparkinsonism and psychotropic drugs, not elsewhere classified[**]~~	~~X61~~
~~Intentional self-poisoning by and exposure to other drugs acting on the autonomic nervous system[**]~~	~~X63~~
~~Intentional self-poisoning by and exposure to other and unspecified drugs, medicaments and biological substances[**]~~	~~X64~~
~~Assault by drugs, medicaments and biological substances[§]~~	~~X85~~
Poisoning, undetermined intent	Y10 - Y14
Drug rehabilitation	Z50.3
Drug abuse counselling and surveillance	Z71.5
Drug use	Z72.2
Environmental/ Domestic substances	

Formatted: French (France)

Mental and behavioural disorders due to use of volatile solvents	F18
Accidental poisoning by and exposure to noxious substances	X40 – X44, X46 - X49
Intentional self-poisoning by and exposure to organic solvents and halogenated hydrocarbons and their vapours[‡]	X66
Intentional self-poisoning by and exposure to noxious substances	X69
Assault by unspecified chemical or noxious substance[§]	X90
Poisoning by chemical or noxious substance, undetermined intent	Y16 - Y19
Codes mentioning both alcohol and drugs	
Special epileptic syndromes - (related to alcohol, drugs, etc.)	G40.5
Blood-alcohol and blood-drug test	Z04.0
Personal history of psychoactive substance abuse - (alcohol, drug, tobacco)[***]	Z86.4
Alcohol	
Alcohol-induced pseudo-Cushing's syndrome	E24.4
Mental and behavioural disorders due to use of alcohol	F10
Degeneration of nervous system due to alcohol	G31.2
Alcoholic polyneuropathy	G62.1
Alcoholic myopathy	G72.1
Alcoholic cardiomyopathy	I42.1
Alcoholic gastritis	K29.2
Alcoholic liver disease	K70
Alcohol-induced acute pancreatitis	K85.2
Alcohol-induced chronic pancreatitis	K86.0
Maternal care for (suspected) damage to fetus from alcohol	O35.4
Finding of alcohol in blood	R78.0
Poisoning: antidotes and chelating agents, not elsewhere classified	T50.6
Toxic effect of alcohol	T51
Accidental poisoning by exposure to alcohol	X45
Poisoning by exposure to alcohol, undetermined intent	Y15
Evidence of alcohol involvement determined by blood alcohol level	Y90
Evidence of alcohol involvement determined by level of intoxication	Y91
Alcohol rehabilitation	Z50.2
Alcohol abuse counselling and surveillance	Z71.4
Alcohol use	Z72.1
**Accidents**	
Transport accidents	V01 - V99

For peer review only

Falls	W00 - W19
Exposure to inanimate mechanical forces	W20 - W49
Exposure to animate mechanical forces	W50 - W64
Accidental drowning and submersion	W65 - W74
Other accidental threats to breathing	W75 - W84
Exposure to electric current, radiation and extreme ambient air temperature and pressure	W85 - W99
Exposure to smoke, fire and flames	X00 - X09
Contact with heat and hot substances	X10 - X19
Contact with venomous animals and plants	X20 - X29
Exposure to forces of nature	X30 - X39
Accidental poisoning by and exposure to noxious substances	X40 - X49
Overexertion, travel and privation	X50 - X57
Accidental exposure to other and unspecified factors	X58 - X59

For peer review only

* ‘Strikethrough’ (Phrase, code) indicates that this was not included in the adversity definition.
† Codes relating to foetuses or neonates (P04.3, Q86.0, P58.4 and P96) were not expected in adolescent presentations; this was checked and did not appear.
** Formed part of the self-harm cluster only; self-poisoning considered most egregious element of diagnosis.
‡ If subject under 18 years old.
§ Formed part of the victimisation cluster only; assault considered most egregious element of the diagnosis.
***Not possible to distinguish whether for drugs, alcohol or tobacco

For peer review only

Supplementary Table 3. Population prevalence* (per 100,000) of emergency admission(s) for injury, by types of adversity within age periods† ** and sex

	All girls	All boys	Girls			Boys		
**Adolescent group****†	**10-19y**	**10-19y**	**10-14y**	**15-17y**	**18-19y**	**10-14y**	**15-17y**	**18-19y**
All	9,0738.66	16,333‡16.27‡	4,1043.92	3,260§3.12§	2,189§2.11§	7,316‡7.67‡	5,069‡§5.29‡§	3,852‡§0.40‡§
**AdvRIAdversity**	**4,5824.37**	**4,108‡4.26‡**	**1,2401.31**	**2,124§2.04§**	**1,329§1.27§**	**669‡0.72‡**	**1,458‡§1.53‡§**	**1,748‡§1.74‡§**
Any Victimisationviolence	3910.76	1,970‡2.20‡	1030.25	152§0.31§	1100.19§	277‡0.33‡	731‡§0.80‡§	833‡§0.90‡§
Any sSelf-harm	3,4183.26	1,266‡1.33‡	7770.90	1,463§1.58§	923§0.97§	170‡0.22‡	393‡§0.47‡§	478‡§0.55‡§
Any dDrug or Aalcohol mMisuse	4,1943.98	2,463‡2.47‡	1,1121.16	1,959§1.87§	1,221§1.15§	375‡0.39‡	807‡§0.84‡§	1,077‡§1.01‡§
**Single adversity**	**1,2541.15**	**2616‡,2.72‡**	**4950.44**	**687§0.49§**	**412§0.32§**	**5160.53‡**	**991‡§0.99‡§**	**1,114‡§1.08‡§**
Victimisation Violence oOnly	2350.24	1,496‡1.63‡	770.09	99§0.10§	750.08§	256‡0.28‡	609‡§0.64‡§	635‡§0.69‡§
Self-harm oOnly	1440.13	1360.14	480.05	63§0.06§	31§0.03§	37‡0.05	390.04‡§	340.04‡
Drug or aAlcohol mMisuse oOnly	8740.78	984‡0.95‡	3690.29	525§0.33§	305§0.21§	224‡0.21‡	342‡§0.31‡§	445‡§0.35‡§
**Multiple adversity**	**3,3283.22**	**1,492‡1.54‡**	**7450.88**	**1,436§1.55§**	**917§0.95§**	**153‡0.19‡**	**467‡§0.54‡§**	**634‡§0.66‡§**
V+SH	80.02	13‡0.02	30.01	30.01	1§0.004§	1‡0.005‡	20.01§	20.01‡
V+DA	540.08	361‡0.35‡	170.03	37§0.03	26§0.02§	190.02‡	114‡§0.11‡§	190‡§0.15‡§
SH+DA	3,1722.70	1,018‡0.97‡	7200.72	1,384§1.34§	882§0.84§	131‡0.14‡	345‡§0.37‡§	436‡§0.45‡§
V+SH+DA	930.42	100‡0.20‡	60.12	13§0.17§	80.09§	2‡0.02‡	5‡§0.05‡§	6§0.06‡§
No adversity	4,4914.29	11,417‡12.01‡	2,6762.79	1,136§1.08§	860§0.84§	6,648‡6.95‡	3,611‡3.76‡§	2,171‡§2.26‡§
**AccidentsRI oOnly**	**7480.71**	**10,466‡1.00‡**	**2,2210.32**	**804§0.26§**	**580§0.24§**	**5,887‡0.47‡**	**3,109‡0.36‡§**	**1,784‡§0.31‡§**
**Other cCauses**	**3,7423.58**	**951‡11.01‡**	**4552.47**	**332§0.82§**	**279§0.60§**	**761‡6.48‡**	**503‡3.40‡§**	**388‡§1.95‡§**

For peer review only

V = ~~Victimisation~~Violence, SH = Self-harm, DA = Drug or Alcohol Misuse~~, AdvRI = Adversity-related Injury, AccRI Only = Accident-related Injury Only~~
*Due to large denominators, confidence intervals for population prevalence were too narrow to provide any meaningful interpretation and so are not shown.
~~‡~~**Each adolescent classified by all adversity/accidents seen at any emergency injury admission(s) at 10-19 years, 10-14 years, 15-17 years and 18-19 years, respectively.
‡Statistically significantly different from girls of the same age (according to 95% confidence intervals).
§Statistically significantly different from 10-14 year olds of the same sex (according to 95% confidence intervals).

For peer review only

REFERENCES

1.Inpatient HES Data Dictionary. Leeds: Health and Social Care Information Centre, 2010.
2.Hardelid P DN, Davey J, Primbramska I, Gilbert R. Overview of child deaths in the four UK countries. London: 2013.

For peer review only

STROBE Statement—checklist of items that should be included in reports of observational studies

	Item No	Recommendation
**Title and abstract**	1	~~(*a*) Indicate the study's design with a commonly used term in the title or the abstract~~
		~~(*b*) Provide in the abstract an informative and balanced summary of what was done and what was found~~
**Introduction**		
Background/rationale	2	~~Explain the scientific background and rationale for the investigation being reported~~
Objectives	3	~~State specific objectives, including any prespecified hypotheses~~
**Methods**		
Study design	4	~~Present key elements of study design early in the paper~~
Setting	5	~~Describe the setting, locations, and relevant dates, including periods of recruitment, exposure, follow-up, and data collection~~
Participants	6	~~(*a*) *Cohort study*—Give the eligibility criteria, and the sources and methods of selection of participants. Describe methods of follow-up~~<br>*Case-control study*—Give the eligibility criteria, and the sources and methods of case ascertainment and control selection. Give the rationale for the choice of cases and controls<br>*Cross-sectional study*—Give the eligibility criteria, and the sources and methods of selection of participants
		(*b*) *Cohort study*—For matched studies, give matching criteria and number of exposed and unexposed. N/A<br>*Case-control study*—For matched studies, give matching criteria and the number of controls per case
Variables	7	~~Clearly define all outcomes, exposures, predictors, potential confounders, and effect modifiers.~~ Give diagnostic criteria, if applicable
Data sources/ measurement	8*	~~For each variable of interest, give sources of data and details of methods of assessment (measurement).~~ Describe comparability of assessment methods if there is more than one group
Bias	9	~~Describe any efforts to address potential sources of bias~~. We only analysed those who could be observed between ages 10 and 19, so no bias due to different lengths of admission trajectories.
Study size	10	~~Explain how the study size was arrived at~~. All available administrative data.
Quantitative variables	11	~~Explain how quantitative variables were handled in the analyses. If applicable, describe which groupings were chosen and why~~
Statistical methods	12	~~(*a*) Describe all statistical methods, including those used to control for confounding~~
		~~(*b*) Describe any methods used to examine subgroups and interactions~~
		(*c*) Explain how missing data were addressed. N/A
		~~(*d*) *Cohort study*—If applicable, explain how loss to follow-up was addressed.~~ We only analysed those who could be observed between ages 10 and 19.<br>*Case-control study*—If applicable, explain how matching of cases and controls was addressed<br>*Cross-sectional study*—If applicable, describe analytical methods taking account of sampling strategy
		(*e*) Describe any sensitivity analyses. N/A

Continued on next page

For peer review only

**Results**		
Participants	13*	~~(a) Report numbers of individuals at each stage of study—eg numbers potentially eligible, examined for eligibility, confirmed eligible, included in the study, completing follow-up, and analysed~~
		(b) Give reasons for non-participation at each stage. N/A
		(c) Consider use of a flow diagram. We didn't use a flow diagram as eligibility could be described using one sentence.
Descriptive data	14*	~~(a) Give characteristics of study participants (eg demographic, clinical, social) and information on exposures and potential confounders~~
		~~(b) Indicate number of participants with missing data for each variable of interest~~
		(c) *Cohort study*—Summarise follow-up time (eg, average and total amount). N/A
Outcome data	15*	~~*Cohort study*—Report numbers of outcome events or summary measures over time.~~ We state how many emergency admissions for adversity-related injury the adversity group had. We also provide a table indicating the number of emergency admissions for injury and admissions (of any type) during ages 10-19.
		Case-control study—Report numbers in each exposure category, or summary measures of exposure
		Cross-sectional study—Report numbers of outcome events or summary measures
Main results	16	~~(*a*) Give unadjusted estimates and, if applicable, confounder-adjusted estimates and their precision (eg, 95% confidence interval). Make clear which confounders were adjusted for and why they were included.~~ We gave prevalence estimates stratified by age-group and sex.
		~~(*b*) Report category boundaries when continuous variables were categorized~~
		(*c*) If relevant, consider translating estimates of relative risk into absolute risk for a meaningful time period. N/A
Other analyses	17	~~Report other analyses done—eg analyses of subgroups and interactions,~~ and sensitivity analyses
**Discussion**		
Key results	18	~~Summarise key results with reference to study objectives~~
Limitations	19	~~Discuss limitations of the study, taking into account sources of potential bias or imprecision. Discuss both direction and magnitude of any potential bias~~
Interpretation	20	~~Give a cautious overall interpretation of results considering objectives, limitations, multiplicity of analyses, results from similar studies, and other relevant evidence~~
Generalisability	21	~~Discuss the generalisability (external validity) of the study results~~
**Other information**		
Funding	22	~~Give the source of funding and the role of the funders for the present study and, if applicable, for the original study on which the present article is based~~

*Give information separately for cases and controls in case-control studies and, if applicable, for exposed and unexposed groups in cohort and cross-sectional studies.

Note: An Explanation and Elaboration article discusses each checklist item and gives methodological background and published examples of transparent reporting. The STROBE checklist is best used in conjunction with this article (freely available on the Web sites of PLoS Medicine at http://www.plosmedicine.org/, Annals of Internal Medicine at http://www.annals.org/, and Epidemiology at http://www.epidem.com/). Information on the STROBE Initiative is available at www.strobe-statement.org.

For peer review only

For peer review only - http://bmjopen.bmj.com/site/about/guidelines.xhtml

Violence, self-harm and drug or alcohol misuse in adolescents admitted to hospitals in England for injury: a retrospective cohort study

Journal:	*BMJ Open*
Manuscript ID:	bmjopen-2014-006079.R2
Article Type:	Research
Date Submitted by the Author:	22-Jan-2015
Complete List of Authors:	Herbert, Anne; UCL Institute of Child Health, Population, Policy and Practice Programme<br>Gilbert, Ruth; UCL Institute of Child Health, Population, Policy and Practice Programme<br>Gonzalez-Izquierdo, Arturo; UCL Institute of Child Health, Population, Policy and Practice Programme<br>Li, Leah; UCL Institute of Child Health, Population, Policy and Practice Programme
**Primary Subject Heading**:	Paediatrics
Secondary Subject Heading:	Mental health, Health services research
Keywords:	MENTAL HEALTH, Non-accidental injury < PAEDIATRICS, Substance misuse < PSYCHIATRY, Suicide & self-harm < PSYCHIATRY

SCHOLARONE™
Manuscripts

For peer review only

Violence, self-harm and drug or alcohol misuse in adolescents admitted to hospitals in England for injury: a retrospective cohort study

Annie Herbert, Centre for Paediatric Epidemiology & Biostatistics, University College London Institute of Child Health, 30 Guilford Street, London, WC1N 1EH, Tel: +44207 905 2790, Fax: +44207 905 2793, annie.herbert.12@ucl.ac.uk

Annie Herbert, M.Sc.[1], *Ruth Gilbert, M.D.*[1], *Arturo González-Izquierdo, Ph.D.*[1], *Leah Li, Ph.D.*[1]

1. Centre for Paediatric Epidemiology & Biostatistics, University College London Institute of Child Health, 30 Guilford Street, London, WC1N 1EH, UK

Short title: Adversity in adolescents admitted to hospital for injury

Key words: Wounds and Injuries, Violence, Self-injurious Behaviour, Substance-related Disorders, Adolescent

Word count: 2,604

No. figures: 2

No tables: 2

No references: 35

No supplementary files for online only publication: 5

For peer review only

ABSTRACT

Objectives

Of adolescents in the general population in England, we aimed to determine 1) the proportion that has an emergency admission to hospital for injury related to adversity (violence, self-harm or drug or alcohol misuse) and 2) the risk of recurrent emergency admissions for injury in adolescents admitted with adversity-related injury compared with those admitted with accident-related injury only.

Design

We used longitudinally-linked administrative hospital data (Hospital Episode Statistics) to identify 10-19 year olds with emergency admissions for injury (including day cases lasting more than four hours), in England in 1998-2011. We used Office for National Statistics mid-year estimates for population denominators.

Results

Approximately 4.3% (n=141 248) of adolescents in the general population (n=3 254 046) had one or more emergency admissions for adversity-related injury (girls 4.6%, boys 4.1%), accounting for 50.0% of all emergency admissions for injury in girls and 29.1% in boys. Admissions for self-harm or drug or alcohol misuse commonly occurred in the same girls and boys. Recurrent emergency admissions for injury were more common in adolescents with adversity-related injury (girls 17.3%; boys 16.5%) than in those with accident-related injury only (girls 4.7%; boys 7.4%), particularly for adolescents with adversity-related injury related to multiple types of adversity (girls 21.1%; boys 24.2%).

For peer review only - http://bmjopen.bmj.com/site/about/guidelines.xhtml

For peer review only

Conclusion

Hospital-based interventions should be developed to reduce the risk of future injury in adolescents admitted for adversity-related injury.

For peer review only

For peer review only - http://bmjopen.bmj.com/site/about/guidelines.xhtml

What is already known on this subject

- Adolescents exposed to adversity (violence, self-harm or drug or alcohol misuse) often use health services, and repetitively.
- Many adolescents in the general population are exposed to multiple types of adversity during their adolescence.

What this study adds

- One in 20 adolescents in England has an emergency admission to hospital for adversity-related injury between 10 and 19 years old.
- Many adolescents admitted with adversity-related injury (particularly girls) are admitted with multiple types of adversity, self-harm and drug or alcohol misuse being the most common combination.
- Re-admission is more common in these adolescents than those admitted with accident-related injury only.

Strengths and limitations of this study

- Hospital Episodes Statistics (HES) captured data on all admissions to National Health Service hospitals in England at 10-19 years old in this study's cohort.
- The longitudinal link between admissions for each individual in HES data allowed us to study the burden of multiple emergency admissions for injury over time.
- However, violence, self-harm and drug or alcohol misuse are not always recognised at an admission, or consistently recorded, and

For peer review only

therefore this study’s estimates of prevalence of adversity are likely to be underestimates.

For peer review only

For peer review only - http://bmjopen.bmj.com/site/about/guidelines.xhtml

INTRODUCTION

Many adolescents exposed to adversity such as violence, self-harm, or drug or alcohol misuse use secondary health services,[1 2] often repetitively.[3 4] For example, in a self-report survey of 15-16 year olds in England, 12.6% of those who had self-harmed had presented to hospital.[2] It is also estimated that approximately one-third of patients attending a hospital in England for self-harm re-attend for self-harm in the following year.[4] Improved management of adolescents exposed to adversity could reduce risk of repetition as well as the burden on secondary care.[5-7]

An admission to hospital provides the 'teachable moment'.[8] That is, both adolescents and their families may be more likely to engage with an intervention than if they had received it elsewhere. Hospital-based interventions to reduce the risk of future harm could benefit these adolescents by reducing episodes of injury, and may reduce recurrent emergency (i.e., acute or unplanned) admissions for injury.

To date, there is a lack of evidence on how different types of adversity-related injury occur in the same adolescents over time. In addition, policy makers and service providers need to know how many adolescents have an emergency admission to hospital for adversity-related injury, their characteristics and their specific rates of re-admission if they are to be feasibly targeted for intervention.

In this study we used administrative hospital data and Office for National Statistics (ONS) mid-year population estimates to estimate the number of adolescents in the general population who have ever had an emergency admission to hospital for injury. We then estimated the

For peer review only - http://bmjopen.bmj.com/site/about/guidelines.xhtml

prevalence of emergency admissions for injury related to violence, self-harm and drug or alcohol misuse (alone and co-occurring) in the general population. Finally, we determined the risk of recurrent emergency admissions for injury in adolescents who had at least one admission between 10 and 19 years for adversity-related injury compared with adolescents only ever admitted for accidental injury during the same period.

For peer review only

METHODS

Study population

Using administrative data from all admissions to National Health Service hospitals in England (Hospital Episode Statistics [HES]) in 1998-2011,[9] we derived a retrospective cohort of adolescents who turned 10 years old in 1998-2002, who could be observed throughout adolescence until 19 years old (Supplementary Table 1).[10] Each individual also had to have at least one emergency admission for injury between 10 and 19 years old.

Admission data

The Health and Social Care Information Centre provided pseudonymised data on hospital admissions, use of which did not require Research Ethics Committee approval.[11] An admission is defined by the National Health Service as any hospital case lasting longer than four hours, and so includes long day cases as well as overnight stays. We analysed any hospital transfers or admissions within one day after discharge as the same admission, as previously described.[12] We used the variable for method of admission ('admimeth') to define an emergency admission. We used all International Classification of Diseases-10 (ICD-10) diagnosis codes recorded during an admission to categorise admissions as being for injury related to adversity or an accident (Supplementary Table 2).[13]

Types of injury and age at emergency admission

We defined an emergency admission for injury as being related to adversity, comprising violence (maltreatment/assault/undetermined causes of injury),

For peer review only

self-harm or drug or alcohol misuse, using mutually exclusive clusters of ICD-10 codes (Supplementary Table 2). Violence was defined by previously-validated codes, which would trigger consideration of violence by carers, peers or strangers.[12 14 15] We defined self-harm using codes that mentioned either 'self-harm' or 'self-poisoning'. Drug or alcohol misuse was defined by codes that mentioned 'alcohol', 'drugs', 'noxious substance' or 'solvent'. We defined an injury as being related to accidents only if no adversity codes were present but there were codes from the ICD-10 *Accidents* subchapter (V01-X59).[13]

We grouped age at each admission to reflect age of onset of puberty (10-14 years), age of sitting secondary school exams (15-17 years) and the legal age for buying alcohol (18-19 years).[16-19]

Classification of adolescents according to types of injury and age at emergency admissions

We classed adolescents into groups according to all of their emergency admissions for injury between 10 and 19 years old. Adolescents were classed as belonging to the 'adversity' group (any adversity-related injury between 10 and 19 years old), 'accidents only' group (no adversity-related injury but one or more accident-related injuries) or 'other causes' group (no adversity-related or accident-related injuries) (Supplementary Figure 1). Among adolescents in the adversity group, we determined the proportion exposed to violence-, self-harm- and drug or alcohol misuse-related injury at age 10-19, respectively. We further classified the adversity group into seven mutually exclusive sub-groups: violence only, self-harm only, drug or alcohol misuse only, violence and self-harm, violence and drug or alcohol misuse,

For peer review only - http://bmjopen.bmj.com/site/about/guidelines.xhtml

self-harm and drug or alcohol misuse, and violence, self-harm and drug or alcohol misuse.

We also grouped adolescents as above, according to their emergency admissions for injury at 10-14 years only, 15-17 years only and 18-19 years only (Supplementary Figure 2).

Population denominators

We used ONS mid-year population estimates to derive population denominators.[20 21] These data are freely available online broken down by sex and year of age.

Analyses

We estimated the proportion of adolescents in the general population who had an emergency admission for injury between 10 and 19 years old. We used the number of adolescents in our derived retrospective cohort as the numerator and ONS mid-year estimates for 10 year olds in 1998-2002 as the population denominator. We then calculated these proportions by types of injury at 10-19 years old (adversity-related [adversity group and seven mutually exclusive subgroups] and accidents only [accidents only group]) and by age group, as described above.

We calculated the proportion of adolescents in the adversity group (and subgroups) and in the accidents only group who had an emergency admission for injury twice and three or more times between 10 and 19 years old. We also calculated the proportions of adolescents with two or three or more admissions of any type (including non-emergency and non-injury).

We reported all results separately for girls and boys since differences between girls and boys have been reported for prevalence of adversity in the

For peer review only - http://bmjopen.bmj.com/site/about/guidelines.xhtml

general population.[1 22-24] We calculated 95% confidence intervals for all proportions but did not present them here as they were all too narrow to convey any useful information (within one unit of the sample estimate). Analyses were carried out in StataSE 12.

For peer review only

For peer review only - http://bmjopen.bmj.com/site/about/guidelines.xhtml

186 **RESULTS**

187 There were 1 033 702 adolescents in HES admissions data in 1998-2011, of
188 which 402 916 formed the study cohort (462 476 emergency admission for
189 injury when considering multiple presentations from the same adolescent, 802
190 682 admissions of any type [including non-emergency and non-injury, 662
191 727 of which were overnight stays]) (Table 1), representing 12.4% (402 916/3
192 254 046) of the adolescent population. Twice as many boys as girls had an
193 emergency admission for injury during adolescence (144 158/1 588 942 girls
194 in the population [8.7%]; 258 503/1 665 104 boys [16.3%]).

For peer review only

Table 1. Characteristics of adolescents whose entire ten years of adolescence (ages 10-19) occurred in 1998-2011.

	Adolescent population*	Adolescents with emergency admission(s) for injury between 10 and 19 years old, n (row %)			
**Characteristics**	**Total**	**Total**	**Adversity**	**Accidents only**	**Other causes**
**All**	**3,254,046**	**402,916 (100.0)**	**141,248 (35.1)**	**233,907 (58.1)**	**27,761 (6.9)**
**Age****					
**Girls**	**1,588,942**	**144,158 (100.0)**	**72,805 (50.5)**	**59,528 (41.3)**	**11,888 (8.2)**
10-14 years	.	65,208 (100.0)	23,178 (35.5)	37,388 (57.3)	4,642 (7.1)
15-17 years	.	48,286 (100.0)	31,573 (65.4)	12,922 (26.8)	3,791 (7.9)
18-19 years	.	30,664 (100.0)	18,054 (58.9)	9,155 (29.9)	3,455 (11.3)
**Boys**	**1,665,104**	**258,503 (100.0)**	**68,403 (26.5)**	**174,267 (67.4)**	**15,833 (6.1)**
10-14 years	.	121,821 (100.0)	17,667 (14.5)	97,478 (80.0)	6,676 (5.5)
15-17 years	.	79,223 (100.0)	25,014 (31.6)	49,345 (62.3)	4,864 (6.1)
18-19 years	.	57,459 (100.0)	25,722 (44.8)	27,444 (47.8)	4,293 (7.5)
Missing (Sex)	.	255 (100.0)	40 (15.7)	175 (68.6)	40 (15.7)

*Based on ONS mid-year England statistics for 10 year olds in 1998-2002.[20]
**At first emergency admission for injury

For peer review only

For peer review only - http://bmjopen.bmj.com/site/about/guidelines.xhtml

Types of injury and age at emergency admission

One-third of the cohort (141 248, 4.3% of the population) had a record of an emergency admission for adversity-related injury between 10 and 19 years old (the adversity group; 157 004 emergency admissions for adversity-related injury in total) (Table 1), with similar rates between sexes (72 805, 4.6% girls in the population; 68 403, 4.1% boys).

The remaining two-thirds of the cohort (261 668, 8.1% of the adolescent population) had emergency admissions for injury which were never related to adversity (Table 1). Among these adolescents, 233 907 (89.4%) had an accident-related injury (the accidents only group, 7.2% of the population) and 27 761 (10.6%) had no accident-related injury (other causes group, 0.9% of the population). A high proportion of the other causes group were affected by a chronic condition[1] between 10 and 19 years old (11 221/27 761, 40.4%), compared with the adversity group (45 321/141 248, 32.1%) or accidents only group (49 434/233 907, 21.1%).

Proportions of adolescents in the general population and within individual age groups by adversity [and subgroups], accidents only, and other causes groups are provided in Supplementary Table 3.

Types of adversity-related injury

Among adolescents in the adversity group (girls 72 805, boys 68 403) (Figure 1), the most common type of adversity was drug or alcohol misuse (girls 91.5%, boys 60.0%). A higher proportion of boys than girls were exposed to

[1] Defined by ICD-10 codes (Supplementary Table 2)

For peer review only - http://bmjopen.bmj.com/site/about/guidelines.xhtml

violence (girls 8.5%, boys 47.6%), but a higher proportion of girls than boys were exposed to self-harm (girls 74.6%; boys 32.9%).

Girls in the adversity group were most likely to be exposed to multiple types of adversity between 10 and 19 years old (69.2% + 2.0% + 1.2% + 0.2% = 72.6%) (Figure 1), especially self-harm and drug or alcohol misuse (69.2% of the entire adversity group, i.e., most of the 72.6%). Fewer boys in the adversity group were exposed to multiple types of adversity (38.4%), the most common combination also being self-harm and drug or alcohol misuse (24.8%).

For most of the adolescents who were exposed to multiple types of adversity, the combination of types was recorded at the same admission. For example, among the 130 adolescent girls who were exposed to violence and self-harm between 10 and 19 years old (Table 2), 64.6% had both violence and self-harm codes present simultaneously in at least one emergency admission for injury (violence and drug or alcohol misuse 78.8%, self-harm and drug or alcohol misuse 99.7%, violence, self-harm and drug or alcohol misuse 33.9%; boys: violence and self-harm 40.1%, violence and drug or alcohol misuse 84.1%, self-harm and drug or alcohol misuse 99.1%, violence, self-harm and drug or alcohol misuse 20.0%) (data not shown).

For peer review only - http://bmjopen.bmj.com/site/about/guidelines.xhtml

Table 2. Proportion of adolescents with 1, 2 or 3+ emergency admission(s) for injury or 1, 2 or 3+ admission(s) of any type, by types of adversity between 10 and 19 years old*

	Girls (%)							Boys (%)						
		Emergency admission(s) for injury			Admission(s) of any type				Emergency admission(s) for injury			Admission(s) of any type		
**Adolescent group***	**No. girls**	**1**	**2**	**3+**	**1**	**2**	**3+**	**No. boys**	**1**	**2**	**3+**	**1**	**2**	**3+**
**All**	**144,158**	**88.6**	**8.3**	**3.1**	**57.6**	**20.5**	**22.0**	**258,503**	**90.3**	**8.0**	**1.8**	**68.5**	**18.7**	**12.9**
**Adversity**	**72,805**	**82.7**	**12.0**	**5.3**	**53.8**	**21.1**	**25.1**	**68,403**	**83.5**	**12.4**	**4.1**	**64.8**	**19.4**	**15.8**
Any violence	6,211	77.2	13.9	8.9	49.1	20.7	30.1	32,799	83.2	12.8	4.0	65.6	19.6	14.8
Any self-harm	54,315	79.3	13.9	6.8	51.2	21.5	27.3	21,087	76.7	15.8	7.5	57.0	20.7	22.3
Any drug or alcohol misuse	66,645	81.9	12.5	5.6	53.6	21.1	25.3	41,014	81.1	13.6	5.3	62.9	19.5	17.6
**Single adversity**	**19,924**	**92.8**	**6.2**	**1.0**	**61.2**	**19.8**	**19.0**	**43,563**	**71.3**	**8.3**	**1.8**	**55.8**	**15.2**	**10.3**
Violence only	3,734	92.4	6.3	1.3	58.1	20.4	21.6	24,912	87.1	10.6	2.2	68.3	19.2	12.6
Self-harm only	2,296	90.3	8.1	1.6	54.4	20.7	24.9	2,260	87.3	10.4	2.3	62.3	19.8	17.9
Drug or alcohol misuse only	13,894	93.3	5.9	0.8	63.2	19.5	17.4	16,391	88.5	9.5	2.1	69.9	17.9	12.2
**Multiple adversity**	**52,881**	**78.9**	**14.2**	**6.9**	**51.0**	**21.6**	**27.4**	**24,840**	**77.3**	**16.7**	**7.5**	**59.0**	**21.0**	**21.5**
V + SH	130	70.0	20.8	9.2	42.3	26.2	31.5	217	41.6	15.4	6.1	32.8	16.0	14.2

For peer review only

For peer review only - http://bmjopen.bmj.com/site/about/guidelines.xhtml

V + DA	862	81.9	15.1	3.0	52.2	22.9	24.9	6,013	84.6	17.5	5.4	68.4	21.8	17.3
SH + DA	50,404	80.1	13.7	6.3	51.8	21.6	26.6	16,953	86.7	16.4	6.9	64.6	22.7	22.9
V + SH + DA	1,485	36.8	31.6	31.5	25.5	19.9	54.5	1,657	22.0	16.4	14.1	17.9	11.7	22.8
No adversity	71,353	94.7	4.5	0.8	61.4	19.9	18.7	190,100	92.4	6.3	0.9	69.5	18.3	11.8
**Accidents only**	**59,465**	**95.3**	**4.1**	**0.6**	**66.5**	**18.7**	**14.7**	**174,267**	**92.3**	**6.5**	**0.9**	**71.1**	**18.0**	**10.5**
**Other causes**	**11,888**	**91.5**	**6.3**	**2.1**	**35.5**	**25.8**	**38.8**	**15,833**	**93.4**	**5.0**	**1.6**	**51.5**	**22.1**	**26.3**

V = Violence, SH = Self-harm, DA = Drug or Alcohol Misuse

* Each adolescent classified by all adversity/accidents seen at any emergency admission(s) for injury between 10 and 19 years old.

For peer review only

For peer review only - http://bmjopen.bmj.com/site/about/guidelines.xhtml

Emergency re-admissions for injury

Adolescent girls in the adversity group (50.5% of all girls in the cohort) accounted for 50.0% of the total number of emergency admissions for injury coming from girls (data not shown), compared with girls in the accidents only group (41.3% of all girls) who accounted for 36.6%. Boys in the adversity group (26.2% of all boys in the cohort) accounted for 29.1% compared with 65.0% contributed by boys in the accidents only group (67.7% of all boys).

More adolescents in the adversity group were re-admitted for injury (i.e., had two or more emergency admissions for injury) between 10 and 19 years old (girls 17.3%, boys 16.5%) (Figure 2), than in the accidents only group (girls 4.7%; boys 7.4%). Among adolescents admitted for injuries related to multiple types of adversity (Table 2), the proportion re-admitted was even higher (multiple types: girls 21.1%, boys 24.2%; single type: girls 7.2%, boys 10.1%).

Similarly, a higher proportion of adolescents in the adversity group had two more admissions of any type (including non-emergency and non-injury) between 10 and 19 years old (girls 46.2%; boys 35.2%) (Table 2) compared with adolescents in the accidents only group (girls 33.4%; boys 28.5%). This proportion was even higher for adolescents in the adversity group who were admitted with multiple types of adversity (multiple types: girls 49.0%, boys 42.5%; single type: girls 38.8%, boys 25.5%).

For peer review only - http://bmjopen.bmj.com/site/about/guidelines.xhtml

DISCUSSION

More than one in 20 adolescents in England had at least one emergency admission for adversity-related injury between 10 and 19 years old. These adolescents accounted for a third of all adolescents with emergency admissions for injury and for a disproportionate number of re-admissions for injury, particularly adolescents admitted with multiple types of adversity-related injury. Targeting adolescents admitted with adversity-related injury could reduce their risk of future harm, the rate of re-admissions to hospital, and health care costs .[25]

Longitudinally-linked admissions allowed us to study the entire ten years of adolescence in 402 916 individuals. We were able to distinguish between types of adversity that co-occurred during adolescence or at the same admission, and to study re-admissions. One weakness of this study was our reliance on diagnostic codes recorded in administrative data. Violence by carers, which could be coded under maltreatment, and drug or alcohol misuse have been shown to be under-recorded using ICD-10,[26 27] but false positives are rare.[15] To address under-recording, we used what we considered to be sensitive clusters of codes for adversity. Other factors related to recording or coding practices,[12 14 15 27 28] for example, new guidelines for defining maltreatment,[14] can also affect ascertainment. Because of the relative insensitivity but good specificity of the coding clusters, some adolescents who were classified in the accidents only group may in fact belong to the adversity group, but did not have their adversity recognised or recorded. Consequently, our prevalence estimates of admission for different types of adversity-related injury are likely to provide a lower bound for the true

For peer review only - http://bmjopen.bmj.com/site/about/guidelines.xhtml

prevalence. Further, as adolescents exposed to adversity who attended the accident and emergency (A&E) department were not necessarily admitted, our prevalence estimates represent adolescents at the severe end of the adversity spectrum. Such analyses of A&E data are limited by the quality of these data in England (available since 2007) and the resulting problems with identifying reasons for presentation and accurately linking individuals to long-term outcomes.[29]

Our prevalence estimates of admission for injury related to individual types of adversity from the general adolescent population are consistent with previous reports for emergency admissions for assault-related injury in 2004-2009 and for all admissions (emergency and non-emergency) for self-harm and drug or alcohol misuse.[1 2 24 30] Previous studies have reported higher rates of drug or alcohol misuse in boys than in girls in the general adolescent population.[24] We found higher rates in girls. This difference could indicate that girls exposed to drug or alcohol misuse are more likely to be injured, to present to hospital, or to be admitted after a hospital presentation, than boys.

Our estimated rates of re-admission of any type (including non-emergency and non-injury) for violence (girls 50.8%, boys 34.4%) (Table 2) and self-harm (girls 48.8%, boys 43.0%) were higher than previously reported (11% for violence, 33% for self-harm).[3 4] These discrepancies are likely to be because we considered the whole ten years of adolescence and re-admission of any type, whereas previous studies looked at re-attendance up to the following year and for the same type of adversity-related injury.[3 4]

The results of this study should inform policy initiatives and national guidelines. Firstly, a substantial proportion of adolescents are affected by

For peer review only - http://bmjopen.bmj.com/site/about/guidelines.xhtml

adversity and they account for a large proportion (29.1 to 50.0%) of all emergency admissions to hospital for injury in this age group. Secondly, we show the large burden of injury admission for all three types of adversity, yet there are currently no national clinical guidelines for managing cases of violence, other than responding to violence by caregivers.[31] Finally, these results show that adolescents often present with multiple types of adversity (especially in girls), even though guidelines exist only for managing individual problems.[32-34]

In addition, policy makers need to be aware of the widely varying aetiological pathways to admission with adversity-related injury. Our approach to defining this group of adolescents is not designed to reflect the complexity or severity of these cases. For example, admission for multiple types of adversity-related injury is a poor proxy indicator of severity. Effective interventions will need to be tailored to the individual based on specialist clinical assessment. However, all three types of adversity are likely to reflect a combination of underlying psychosocial need and environmental and social stressors.[35]

Hospital interventions may reduce the risk of future harm, including the incidence of other types of harm not seen in hospital, e.g., further adversity-related injury not leading to admission. Further research using linked data from health care sectors such as accident and emergency, could shed light on the overall burden of adversity-related injury on hospitals. Though these data have limited quality in England, longitudinally-linked datasets in other countries could provide insights into long-term outcomes for this vulnerable group of adolescents.

For peer review only - http://bmjopen.bmj.com/site/about/guidelines.xhtml

Contributors

Annie Herbert – helped to conceive and design the study, analysed and interpreted the data, drafted the article, revised it critically for important intellectual content and approved the final version to be published.

Leah Li – helped to conceive and design the study, interpreted the data, revised the article critically for important intellectual content and approved the final version to be published.

Arturo González-Izquierdo – helped to conceive and design the study, acquired and interpreted the data, revised the article critically for important intellectual content and approved the final version to be published.

Ruth Gilbert – helped to conceive and design the study, acquired and interpreted the data, revised the article critically for important intellectual content and approved the final version to be published.

Competing interests None.

Funding AH was supported by the Policy Research Unit in the Health of Children, Young People and Families, which is funded by the Department of Health Policy Research Programme (grant reference number: 109/0001). This report is an independent research commissioned by the Department of Health. The views expressed in this publication are those of the authors and not necessarily those of the Department of Health. AH is also supported by the University College London IMPACT studentship. RG is supported by awards establishing the Farr Institute of Health Informatics Research at University College London Partners from the Medical Research Council and a

For peer review only - http://bmjopen.bmj.com/site/about/guidelines.xhtml

consortium of funders (MR/K006584/1). The study funders played no part in the design, data analysis and interpretation of this study; the writing of the manuscript or the decision to submit the paper for publication, and the authors’ work was independent of their funders.

Ethics Both Hospital Episode Statistics (HES) admissions data and Office for National Statistics (ONS) mid-year population estimates are derived from routinely collected administrative data. HES data were pseudonymised before we received them and therefore we did not require Research Ethics Committee approval.

Data sharing No additional data on HES are available. ONS mid-year population estimates may be accessed freely online: http://www.ons.gov.uk/ons/publications/all-releases.html?definition=tcm%3A77-22371.

For peer review only

For peer review only - http://bmjopen.bmj.com/site/about/guidelines.xhtml

REFERENCES

1. Bellis MA, Hughes K, Wood S, et al. National five-year examination of inequalities and trends in emergency hospital admission for violence across England. Injury prevention : journal of the International Society for Child and Adolescent Injury Prevention 2011;**17**(5):319-25 doi: 10.1136/ip.2010.030486[published Online First: Epub Date]|.
2. Hawton K, Rodham K, Evans E, et al. Deliberate self harm in adolescents: self report survey in schools in England. Bmj 2002;**325**(7374):1207-11
3. Meuleners LB, Lee AH, Hendrie D. Interpersonal violence hospitalisations for adolescents: A population-based study. Journal of paediatrics and child health 2010;**46**(11):686-90 doi: 10.1111/j.1440-1754.2010.01818.x[published Online First: Epub Date]|.
4. Lilley R, Owens D, Horrocks J, et al. Hospital care and repetition following self-harm: multicentre comparison of self-poisoning and self-injury. The British Journal of Psychiatry 2008;**192**(6):440-45
5. Snider C, Lee J. Youth violence secondary prevention initiatives in emergency departments: a systematic review. Cjem 2009;**11**(2):161-8
6. Ougrin D, Boege I, Stahl D, et al. Randomised controlled trial of therapeutic assessment versus usual assessment in adolescents with self-harm: 2-year follow-up. Archives of disease in childhood 2013;**98**(10):772-6 doi: 10.1136/archdischild-2012-303200[published Online First: Epub Date]|.
7. Crawford MJ, Patton R, Touquet R, et al. Screening and referral for brief intervention of alcohol-misusing patients in an emergency department: a pragmatic randomised controlled trial. The Lancet;**364**(9442):1334-39 doi: http://dx.doi.org/10.1016/S0140-6736(04)17190-0[published Online First: Epub Date]|.
8. Johnson SB, Bradshaw CP, Wright JL, et al. Characterizing the teachable moment: is an emergency department visit a teachable moment for intervention among assault-injured youth and their parents? Pediatric emergency care 2007;**23**(8):553-59
9. Inpatient HES Data Dictionary. Leeds: Health and Social Care Information Centre, 2010.

For peer review only - http://bmjopen.bmj.com/site/about/guidelines.xhtml

10. World Health Organisation. Adolescent Health. Secondary Adolescent Health 2013. http://www.who.int/topics/adolescent_health/en/.
11. National Health Service Act, 2006.
12. Gonzalez-Izquierdo A, Woodman J, Copley L, et al. Variation in recording of child maltreatment in administrative records of hospital admissions for injury in England, 1997-2009. Archives of disease in childhood 2010;**95**(11):918-25 doi: 10.1136/adc.2009.180216[published Online First: Epub Date]|.
13. International Statistical Classification of Diseases and Related Health Problems 10th Revision. Secondary International Statistical Classification of Diseases and Related Health Problems 10th Revision. http://apps.who.int/classifications/icd10/browse/2010/en.
14. Saperia J, Lakhanpaul M, Kemp A, et al. When to suspect child maltreatment: summary of NICE guidance. Bmj 2009;**339**:b2689 doi: 10.1136/bmj.b2689[published Online First: Epub Date]|.
15. Gonzalez-Izquierdo A, Ward A, M OD, et al. Cross-country comparison of victimisation-related injury admission in children and adolescents in England and Western Australia. BMC Health Serv Res 2013;**13**(1):260 doi: 10.1186/1472-6963-13-260[published Online First: Epub Date]|.
16. Patton GC, Viner R. Pubertal transitions in health. Lancet 2007;**369**(9567):1130-9 doi: 10.1016/S0140-6736(07)60366-3[published Online First: Epub Date]|.
17. Gill T. How old are GCSE candidates? Statistics Report Series, 2010.
18. Directgov. AS and A levels. Secondary AS and A levels 2012. http://webarchive.nationalarchives.gov.uk/20121015000000/http://www.direct.gov.uk/en/EducationAndLearning/QualificationsExplained/DG_10039018.
19. Buying Alcohol. Secondary Buying Alcohol 2013. https://www.drinkaware.co.uk/check-the-facts/alcohol-and-the-law/buying-alcohol.
20. All releases of Population Estimates for UK, England and Wales, Scotland and Northern Ireland: Office for National Statistics, 2013.
21. Office for National Statistics. Mid-year population estimates short methods guide: Office for National Statistics, 2010.
22. Radford L. Child abuse and neglect in the UK today. NSPCC Research: NSPCC, 2011.

For peer review only - http://bmjopen.bmj.com/site/about/guidelines.xhtml

23. *Truth hurts: report of the national inquiry into self-harm among young people*. London: Mental Health Foundation, 2006.
24. Hibell B, Guttormsson U, Ahlström S, et al. The 2011 ESPAD Report. Substance use among students in 2012;**36**
25. National Audit Office. Emergency admissions to hospital: managing the demand, 2013.
26. Scott D, Tonmyr L, Fraser J, et al. The utility and challenges of using ICD codes in child maltreatment research: A review of existing literature. Child abuse & neglect 2009;**33**(11):791-808 doi: S0145-2134(09)00202-6 [pii]
10.1016/j.chiabu.2009.08.005[published Online First: Epub Date]|.
27. McKenzie K, Harrison JE, McClure RJ. Identification of alcohol involvement in injury-related hospitalisations using routine data compared to medical record review. Australian and New Zealand journal of public health 2010;**34**(2):146-52 doi: 10.1111/j.1753-6405.2010.00499.x[published Online First: Epub Date]|.
28. Farrar S, Yi D, Sutton M, et al. Has payment by results affected the way that English hospitals provide care? Difference-in-differences analysis. Bmj 2009;**339** doi: 10.1136/bmj.b3047[published Online First: Epub Date]|.
29. Hospital Episode Statistics Accident & Emergency (England), 2007-. Secondary Hospital Episode Statistics Accident & Emergency (England), 2007-. http://adrn.ac.uk/catalogue/cataloguepage?sn=888040.
30. Hawton K, Rodham K, Evans E, et al. Adolescents Who Self Harm: A Comparison of Those Who Go to Hospital and Those Who Do Not. Child Adol Ment H-Uk 2009;**14**(1):24-30 doi: DOI 10.1111/j.1475-3588.2008.00485.x[published Online First: Epub Date]|.
31. What to do if you're worried that a child is being abused. In: Skills DfE, ed.: DfES Publications, 2006.
32. Self-harm: longer term management [Clinical Guideline 133]. London: National Institute for Health and Clinical Excellence, 2011.
33. Drug misuse : psychosocial interventions [Clinical Guideline 51]. London: National Institute for Health and Clinical Excellence,, 2007.
34. Alcohol-use disorder: diagnosis, assessment and management of harmful drinking and alcohol dependence [Clinical Guideline 115]. London: National Institute for Health and Clinical Excellence, 2011.

For peer review only - http://bmjopen.bmj.com/site/about/guidelines.xhtml

35. Viner RM, Ozer EM, Denny S, et al. Adolescence and the social determinants of health. Lancet 2012;**379**(9826):1641-52 doi: 10.1016/S0140-6736(12)60149-4[published Online First: Epub Date]|.

For peer review only

Violence, self-harm and drug or alcohol misuse in adolescents admitted to hospitals in England for injury: a retrospective cohort study

Annie Herbert, Centre for Paediatric Epidemiology & Biostatistics, University College London Institute of Child Health, 30 Guilford Street, London, WC1N 1EH, Tel: +44207 905 2790, Fax: +44207 905 2793, annie.herbert.12@ucl.ac.uk

Annie Herbert, M.Sc.[1], *Ruth Gilbert, M.D.*[1], *Arturo González-Izquierdo, Ph.D.*[1], *Leah Li, Ph.D.*[1]

1. Centre for Paediatric Epidemiology & Biostatistics, University College London Institute of Child Health, 30 Guilford Street, London, WC1N 1EH, UK

Short title: Adversity in adolescents admitted to hospital for injury

Key words: Wounds and Injuries, Violence, Self-injurious Behaviour, Substance-related Disorders, Adolescent

Word count: 2,~~565~~604

No. figures: 2

No tables: 2

No references: 35

No supplementary files for online only publication: 5

For peer review only

For peer review only - http://bmjopen.bmj.com/site/about/guidelines.xhtml

ABSTRACT

Objectives

Of adolescents in the general population in England, we aimed to determine 1) the proportion that has an emergency admission to hospital for injury related to adversity (violence, self-harm or drug or alcohol misuse) and 2) the risk of recurrent emergency admissions for injury in adolescents admitted with adversity-related injury compared with those admitted with accident-related injury only.

Design

We used longitudinally-linked administrative hospital data (Hospital Episode Statistics) to identify 10-19 year olds with emergency admissions for injury (including day cases lasting more than four hours), in England in 1998-2011. We used Office for National Statistics mid-year estimates for population denominators.

Results

Approximately 4.3% (n=141 248) of adolescents in the general population (n=3 254 046) had one or more emergency admissions for adversity-related injury (girls 4.6%, boys 4.1%), accounting for 50.0% of all emergency admissions for injury in girls and 29.1% in boys. Admissions for self-harm or drug or alcohol misuse commonly occurred in the same girls and boys. Recurrent emergency admissions for injury were more common in adolescents with adversity-related injury (girls 17.3%; boys 16.5%) than in those with accident-related injury only (girls 4.7%; boys 7.4%), particularly for

For peer review only

adolescents with adversity-related injury related to multiple types of adversity (girls 21.1%; boys 24.2%).

Conclusion

Hospital-based interventions should be developed to reduce the risk of future injury in adolescents admitted for adversity-related injury.

For peer review only

For peer review only - http://bmjopen.bmj.com/site/about/guidelines.xhtml

What is already known on this subject

- Adolescents exposed to adversity (violence, self-harm or drug or alcohol misuse) often use health services, and repetitively.
- Many adolescents in the general population are exposed to multiple types of adversity during their adolescence.

What this study adds

- One in 20 adolescents in England has an emergency admission to hospital for adversity-related injury between 10 and 19 years old.
- Many adolescents admitted with adversity-related injury (particularly girls) are admitted with multiple types of adversity, self-harm and drug or alcohol misuse being the most common combination.
- Re-admission is more common in these adolescents than those admitted with accident-related injury only.

Strengths and limitations of this study

- Hospital Episodes Statistics (HES) captured data on all admissions to National Health Service hospitals in England at 10-19 years old in this study's cohort.
- The longitudinal link between admissions for each individual in HES data allowed us to study the burden of multiple emergency admissions for injury over time.
- However, violence, self-harm and drug or alcohol misuse are not always recognised at an admission, or consistently recorded, and

For peer review only

77 therefore this study's estimates of prevalence of adversity are likely to

78 be underestimates.

For peer review only

For peer review only - http://bmjopen.bmj.com/site/about/guidelines.xhtml

INTRODUCTION

Many adolescents exposed to adversity such as violence, self-harm, or drug or alcohol misuse use secondary health services,[1 2] often repetitively.[3 4] For example, in a self-report survey of 15-16 year olds in England, 12.6% of those who had self-harmed had presented to hospital.[2] It is also estimated that approximately one-third of patients attending a hospital in England for self-harm re-attend for self-harm in the following year.[4] Improved management of adolescents exposed to adversity could reduce risk of repetition as well as the burden on secondary care.[5-7]

An admission to hospital provides the ‘teachable moment’.[8] That is, both adolescents and their families may be more likely to engage with an intervention than if they had received it elsewhere. Hospital-based interventions to reduce the risk of future harm could benefit these adolescents by reducing episodes of injury, and may reduce recurrent emergency (i.e., acute or unplanned) admissions for injury.

To date, there is a lack of evidence on how different types of adversity-related injury occur in the same adolescents over time. In addition, policy makers and service providers need to know how many adolescents have an emergency admission to hospital for adversity-related injury, their characteristics and their specific rates of re-admission if they are to be feasibly targeted for intervention.

In this study we used administrative hospital data and Office for National Statistics (ONS) mid-year population estimates to estimate the number of adolescents in the general population who have ever had an emergency admission to hospital for injury. We then estimated the

For peer review only - http://bmjopen.bmj.com/site/about/guidelines.xhtml

prevalence of emergency admissions for injury related to violence, self-harm and drug or alcohol misuse (alone and co-occurring) in the general population. Finally, we determined the risk of recurrent emergency admissions for injury in adolescents who had at least one admission between 10 and 19 years for adversity-related injury compared with adolescents only ever admitted for accidental injury during the same period.

For peer review only

For peer review only - http://bmjopen.bmj.com/site/about/guidelines.xhtml

METHODS

Study population

Using administrative data from all admissions to National Health Service hospitals in England (Hospital Episode Statistics [HES]) in 1998-2011,[9] we derived a retrospective cohort of adolescents who turned 10 years old in 1998-2002, who could be observed throughout adolescence until 19 years old (Supplementary Table 1).[10] Each individual also had to have at least one emergency admission for injury between 10 and 19 years old.

Admission data

The Health and Social Care Information Centre provided pseudonymised data on hospital admissions, use of which did not require Research Ethics Committee approval.[11] An admission is defined by the National Health Service as any hospital case lasting longer than four hours, and so includes long day cases as well as overnight stays. We analysed any hospital transfers or admissions within one day after discharge as the same admission, as previously described.[12] We used the variable for method of admission ('admimeth') to define an emergency admission. We used all International Classification of Diseases-10 (ICD-10) diagnosis codes recorded during an admission to categorise admissions as being for injury related to adversity or an accident (Supplementary Table 2).[13]

Types of injury and age at emergency admission

We defined an emergency admission for injury as being related to adversity, comprising violence (maltreatment/assault/undetermined causes of injury),

For peer review only

self-harm or drug or alcohol misuse, using mutually exclusive clusters of ICD-10 codes (Supplementary Table 2). Violence was defined by previously-validated codes, which would trigger consideration of violence by carers, peers or strangers.[12 14 15] We defined self-harm using codes that mentioned either ‘self-harm’ or ‘self-poisoning’. Drug or alcohol misuse was defined by codes that mentioned ‘alcohol’, ‘drugs’, ‘noxious substance’ or ‘solvent’. We defined an injury as being related to accidents only if no adversity codes were present but there were codes from the ICD-10 *Accidents* subchapter (V01-X59).[13]

We grouped age at each admission to reflect age of onset of puberty (10-14 years), age of sitting secondary school exams (15-17 years) and the legal age for buying alcohol (18-19 years).[16-19]

Classification of adolescents according to types of injury and age at emergency admissions

We classed adolescents into groups according to all of their emergency admissions for injury between 10 and 19 years old. Adolescents were classed as belonging to the ‘adversity’ group (any adversity-related injury between 10 and 19 years old), ‘accidents only’ group (no adversity-related injury but one or more accident-related injuries) or ‘other causes’ group (no adversity-related or accident-related injuries) (Supplementary Figure 1). Among adolescents in the adversity group, we determined the proportion exposed to violence-, self-harm- and drug or alcohol misuse-related injury at age 10-19, respectively. We further classified the adversity group into seven mutually exclusive sub-groups: violence only, self-harm only, drug or alcohol misuse only, violence and self-harm, violence and drug or alcohol misuse,

For peer review only - http://bmjopen.bmj.com/site/about/guidelines.xhtml

self-harm and drug or alcohol misuse, and violence, self-harm and drug or alcohol misuse.

We also grouped adolescents as above, according to their emergency admissions for injury at 10-14 years only, 15-17 years only and 18-19 years only (Supplementary Figure 2).

Population denominators

We used ONS mid-year population estimates to derive population denominators.[20 21] These data are freely available online broken down by sex and year of age.

Analyses

We estimated the proportion of adolescents in the general population who had an emergency admission for injury between 10 and 19 years old. We used the number of adolescents in our derived retrospective cohort as the numerator and ONS mid-year estimates for 10 year olds in 1998-2002 as the population denominator. We then calculated these proportions by types of injury at 10-19 years old (adversity-related [adversity group and seven mutually exclusive subgroups] and accidents only [accidents only group]) and by age group, as described above.

We calculated the proportion of adolescents in the adversity group (and subgroups) and in the accidents only group who had an emergency admission for injury twice and three or more times between 10 and 19 years old. We also calculated the proportions of adolescents with two or three or more admissions of any type (including non-emergency and non-injury).

We reported all results separately for girls and boys since differences between girls and boys have been reported for prevalence of adversity in the

For peer review only

For peer review only - http://bmjopen.bmj.com/site/about/guidelines.xhtml

general population.[1 22-24] We calculated 95% confidence intervals for all proportions but did not present them here as they were all too narrow to convey any useful information (within one unit of the sample estimate). Analyses were carried out in StataSE 12.

For peer review only - http://bmjopen.bmj.com/site/about/guidelines.xhtml

RESULTS

There were 1 033 702 adolescents in HES admissions data in 1998-2011, of which 402 916 formed the study cohort (462 476 emergency admission for injury when considering multiple presentations from the same adolescent, 802 682 admissions of any type [including non-emergency and non-injury, 662 727 of which were overnight stays]) (Table 1), representing 12.4% (402 916/3 254 046) of the adolescent population. Twice as many boys as girls had an emergency admission for injury during adolescence (144 158/1 588 942 girls in the population [8.7%]; 258 503/1 665 104 boys [16.3%]).

For peer review only - http://bmjopen.bmj.com/site/about/guidelines.xhtml

Table 1. Characteristics of adolescents whose entire ten years of adolescence (ages 10-19) occurred in 1998-2011.

	**Adolescent population***	**Adolescents with emergency admission(s) for injury between 10 and 19 years old, n (row %)**			
**Characteristics**	**Total**	**Total**	**Adversity**	**Accidents only**	**Other causes**
**All**	**3,254,046**	**402,916 (100.0)**	**141,248 (35.1)**	**233,907 (58.1)**	**27,761 (6.9)**
**Age****					
**Girls**	**1,588,942**	**144,158 (100.0)**	**72,805 (50.5)**	**59,528 (41.3)**	**11,888 (8.2)**
10-14 years	.	65,208 (100.0)	23,178 (35.5)	37,388 (57.3)	4,642 (7.1)
15-17 years	.	48,286 (100.0)	31,573 (65.4)	12,922 (26.8)	3,791 (7.9)
18-19 years	.	30,664 (100.0)	18,054 (58.9)	9,155 (29.9)	3,455 (11.3)
**Boys**	**1,665,104**	**258,503 (100.0)**	**68,403 (26.5)**	**174,267 (67.4)**	**15,833 (6.1)**
10-14 years	.	121,821 (100.0)	17,667 (14.5)	97,478 (80.0)	6,676 (5.5)
15-17 years	.	79,223 (100.0)	25,014 (31.6)	49,345 (62.3)	4,864 (6.1)
18-19 years	.	57,459 (100.0)	25,722 (44.8)	27,444 (47.8)	4,293 (7.5)
Missing (Sex)	.	255 (100.0)	40 (15.7)	175 (68.6)	40 (15.7)

*Based on ONS mid-year England statistics for 10 year olds in 1998-2002.[20]
**At first emergency admission for injury

For peer review only

For peer review only - http://bmjopen.bmj.com/site/about/guidelines.xhtml

Types of injury and age at emergency admission

One-third of the cohort (141 248, 4.3% of the population) had a record of an emergency admission for adversity-related injury between 10 and 19 years old (the adversity group; 157 004 emergency admissions for adversity-related injury in total) (Table 1), with similar rates between sexes (72 805, 4.6% girls in the population; 68 403, 4.1% boys).

The remaining two-thirds of the cohort (261 668, 8.1% of the adolescent population) had emergency admissions for injury which were never related to adversity (Table 1). Among these adolescents, 233 907 (89.4%) had an accident-related injury (the accidents only group, 7.2% of the population) and 27 761 (10.6%) had no accident-related injury (other causes group, 0.9% of the population). A high proportion of the other causes group were affected by a chronic condition[1] between 10 and 19 years old (11 221/27 761, 40.4%), compared with the adversity group (45 321/141 248, 32.1%) or accidents only group (49 434/233 907, 21.1%).

Proportions of adolescents in the general population and within individual age groups by adversity [and subgroups], accidents only, and other causes groups are provided in Supplementary Table 3.

Types of adversity-related injury

Among adolescents in the adversity group (girls 72 805, boys 68 403) (Figure 1), the most common type of adversity was drug or alcohol misuse (girls 91.5%, boys 60.0%). A higher proportion of boys than girls were exposed to

[1] Defined by ICD-10 codes (Supplementary Table 2)

For peer review only - http://bmjopen.bmj.com/site/about/guidelines.xhtml

violence (girls 8.5%, boys 47.6%), but a higher proportion of girls than boys were exposed to self-harm (girls 74.6%; boys 32.9%).

Girls in the adversity group were most likely to be exposed to multiple types of adversity between 10 and 19 years old (69.2% + 2.0% + 1.2% + 0.2% = 72.6%) (Figure 1), especially self-harm and drug or alcohol misuse (69.2% of the entire adversity group, i.e., most of the 72.6%). Fewer boys in the adversity group were exposed to multiple types of adversity (38.4%), the most common combination also being self-harm and drug or alcohol misuse (24.8%).

For most of the adolescents who were exposed to multiple types of adversity, the combination of types was recorded at the same admission. For example, among the 130 adolescent girls who were exposed to violence and self-harm between 10 and 19 years old (Table 2), 64.6% had both violence and self-harm codes present simultaneously in at least one emergency admission for injury (violence and drug or alcohol misuse 78.8%, self-harm and drug or alcohol misuse 99.7%, violence, self-harm and drug or alcohol misuse 33.9%; boys: violence and self-harm 40.1%, violence and drug or alcohol misuse 84.1%, self-harm and drug or alcohol misuse 99.1%, violence, self-harm and drug or alcohol misuse 20.0%) (data not shown).

For peer review only - http://bmjopen.bmj.com/site/about/guidelines.xhtml

Table 2. Proportion of adolescents with 1, 2 or 3+ emergency admission(s) for injury or 1, 2 or 3+ admission(s) of any type, by types of adversity between 10 and 19 years old*

		Girls (%)							Boys (%)					
		Emergency admission(s) for injury			Admission(s) of any type				Emergency admission(s) for injury			Admission(s) of any type		
**Adolescent group***	**No. girls**	**1**	**2**	**3+**	**1**	**2**	**3+**	**No. boys**	**1**	**2**	**3+**	**1**	**2**	**3+**
**All**	**144,158**	**88.6**	**8.3**	**3.1**	**57.6**	**20.5**	**22.0**	**258,503**	**90.3**	**8.0**	**1.8**	**68.5**	**18.7**	**12.9**
**Adversity**	**72,805**	**82.7**	**12.0**	**5.3**	**53.8**	**21.1**	**25.1**	**68,403**	**83.5**	**12.4**	**4.1**	**64.8**	**19.4**	**15.8**
Any violence	6,211	77.2	13.9	8.9	49.1	20.7	30.1	32,799	83.2	12.8	4.0	65.6	19.6	14.8
Any self-harm	54,315	79.3	13.9	6.8	51.2	21.5	27.3	21,087	76.7	15.8	7.5	57.0	20.7	22.3
Any drug or alcohol misuse	66,645	81.9	12.5	5.6	53.6	21.1	25.3	41,014	81.1	13.6	5.3	62.9	19.5	17.6
**Single adversity**	**19,924**	**92.8**	**6.2**	**1.0**	**61.2**	**19.8**	**19.0**	**43,563**	**71.3**	**8.3**	**1.8**	**55.8**	**15.2**	**10.3**
Violence only	3,734	92.4	6.3	1.3	58.1	20.4	21.6	24,912	87.1	10.6	2.2	68.3	19.2	12.6
Self-harm only	2,296	90.3	8.1	1.6	54.4	20.7	24.9	2,260	87.3	10.4	2.3	62.3	19.8	17.9
Drug or alcohol misuse only	13,894	93.3	5.9	0.8	63.2	19.5	17.4	16,391	88.5	9.5	2.1	69.9	17.9	12.2
**Multiple adversity**	**52,881**	**78.9**	**14.2**	**6.9**	**51.0**	**21.6**	**27.4**	**24,840**	**77.3**	**16.7**	**7.5**	**59.0**	**21.0**	**21.5**
V + SH	130	70.0	20.8	9.2	42.3	26.2	31.5	217	41.6	15.4	6.1	32.8	16.0	14.2

For peer review only

For peer review only - http://bmjopen.bmj.com/site/about/guidelines.xhtml

V + DA	862	81.9	15.1	3.0	52.2	22.9	24.9	6,013	84.6	17.5	5.4	68.4	21.8	17.3
SH + DA	50,404	80.1	13.7	6.3	51.8	21.6	26.6	16,953	86.7	16.4	6.9	64.6	22.7	22.9
V + SH + DA	1,485	36.8	31.6	31.5	25.5	19.9	54.5	1,657	22.0	16.4	14.1	17.9	11.7	22.8
No adversity	71,353	94.7	4.5	0.8	61.4	19.9	18.7	190,100	92.4	6.3	0.9	69.5	18.3	11.8
**Accidents only**	**59,465**	**95.3**	**4.1**	**0.6**	**66.5**	**18.7**	**14.7**	**174,267**	**92.3**	**6.5**	**0.9**	**71.1**	**18.0**	**10.5**
**Other causes**	**11,888**	**91.5**	**6.3**	**2.1**	**35.5**	**25.8**	**38.8**	**15,833**	**93.4**	**5.0**	**1.6**	**51.5**	**22.1**	**26.3**

V = Violence, SH = Self-harm, DA = Drug or Alcohol Misuse

* Each adolescent classified by all adversity/accidents seen at any emergency admission(s) for injury between 10 and 19 years old.

For peer review only - http://bmjopen.bmj.com/site/about/guidelines.xhtml

Emergency re-admissions for injury

Adolescent girls in the adversity group (50.5% of all girls in the cohort) accounted for 50.0% of the total number of emergency admissions for injury coming from girls (data not shown), compared with girls in the accidents only group (41.3% of all girls) who accounted for 36.6%. Boys in the adversity group (26.2% of all boys in the cohort) accounted for 29.1% compared with 65.0% contributed by boys in the accidents only group (67.7% of all boys).

More adolescents in the adversity group were re-admitted for injury (i.e., had two or more emergency admissions for injury) between 10 and 19 years old (girls 17.3%, boys 16.5%) (Figure 2), than in the accidents only group (girls 4.7%; boys 7.4%). Among adolescents admitted for injuries related to multiple types of adversity (Table 2), the proportion re-admitted was even higher (multiple types: girls 21.1%, boys 24.2%; single type: girls 7.2%, boys 10.1%).

Similarly, a higher proportion of adolescents in the adversity group had two more admissions of any type (including non-emergency and non-injury) between 10 and 19 years old (girls 46.2%; boys 35.2%) (Table 2) compared with adolescents in the accidents only group (girls 33.4%; boys 28.5%). This proportion was even higher for adolescents in the adversity group who were admitted with multiple types of adversity (multiple types: girls 49.0%, boys 42.5%; single type: girls 38.8%, boys 25.5%).

For peer review only - http://bmjopen.bmj.com/site/about/guidelines.xhtml

DISCUSSION

More than one in 20 adolescents in England had at least one emergency admission for adversity-related injury between 10 and 19 years old. These adolescents accounted for a third of all adolescents with emergency admissions for injury and for a disproportionate number of re-admissions for injury, particularly adolescents admitted with multiple types of adversity-related injury. Targeting adolescents admitted with adversity-related injury could reduce their risk of future harm, the rate of re-admissions to hospital, and health care costs .[25]

Longitudinally-linked admissions allowed us to study the entire ten years of adolescence in 402 916 individuals. We were able, to distinguish between types of adversity that co-occurred during adolescence or at the same admission, and to study re-admissions. One weakness of this study was our reliance on diagnostic codes recorded in administrative data. Violence by carers, which could be coded under maltreatment, and drug or alcohol misuse have been shown to be under-recorded using ICD-10,[26 27] but false positives are rare.[15] To address under-recording, we used what we considered to be sensitive clusters of codes for adversity. Other factors related to recording or coding practices,[12 14 15 27 28] for example, new guidelines for defining maltreatment,[14] can also affect ascertainment. Because of the relative insensitivity but good specificity of the coding clusters, some adolescents who were classified in the accidents only group may in fact belong to the adversity group, but did not have their adversity recognised or recorded. Consequently, our prevalence estimates of admission for different types of adversity-related injury are likely to provide a lower bound for the true

For peer review only - http://bmjopen.bmj.com/site/about/guidelines.xhtml

prevalence. Further, as adolescents exposed to adversity who attended the accident and emergency (A&E) department were not necessarily admitted, our prevalence estimates represent adolescents at the severe end of the adversity spectrum. Such analyses of A&E data are limited by the quality of these data in England (available since 2007) and the resulting problems with identifying reasons for presentation and accurately linking individuals to long-term outcomes.[29]

Our prevalence estimates of admission for injury related to individual types of adversity from the general adolescent population are consistent with previous reports for emergency admissions for assault-related injury in 2004-2009 and for all admissions (emergency and non-emergency) for self-harm and drug or alcohol misuse.[1 2 24 30] Previous studies have reported higher rates of drug or alcohol misuse in boys than in girls in the general adolescent population.[24] We found higher rates in girls. This difference could indicate that girls exposed to drug or alcohol misuse are more likely to be injured, to present to hospital, or to be admitted after a hospital presentation, than boys.

Our estimated rates of re-admission of any type (including non-emergency and non-injury) for violence (girls 50.8%, boys 34.4%) (Table 2) and self-harm (girls 48.8%, boys 43.0%) were higher than previously reported (11% for violence, 33% for self-harm).[3 4] These discrepancies are likely to be because we considered the whole ten years of adolescence and re-admission of any type, whereas previous studies looked at re-attendance up to the following year and for the same type of adversity-related injury.[3 4]

The results of this study should inform policy initiatives and national guidelines. Firstly, a substantial proportion of adolescents are affected by

For peer review only - http://bmjopen.bmj.com/site/about/guidelines.xhtml

adversity and they account for a large proportion (29.1 to 50.0%) of all emergency admissions to hospital for injury in this age group. Secondly, we show the large burden of injury admission for all three types of adversity, yet there are currently no national clinical guidelines for managing cases of violence, other than responding to violence by caregivers.[31] Finally, these results show that adolescents often present with multiple types of adversity (especially in girls), even though guidelines exist only for managing individual problems.[32-34]

In addition, policy makers need to be aware of the widely varying aetiological pathways to admission with adversity-related injury. Our approach to defining this group of adolescents is not designed to reflect the complexity or severity of these cases. For example, admission for multiple types of adversity-related injury is a poor proxy indicator of severity. Effective interventions will need to be tailored to the individual based on specialist clinical assessment. However, all three types of adversity are likely to reflect a combination of underlying psychosocial need and environmental and social stressors.[35]

Hospital interventions may reduce the risk of future harm, including the incidence of other types of harm not seen in hospital, e.g., further adversity-related injury not leading to admission. Further research using linked data from health care sectors such as accident and emergency, could shed light on the overall burden of adversity-related injury on hospitals. Though these data have limited quality in England, ~~Such analyses are limited by the quality of accident and emergency data in England (available since 2007) and the resulting problems with identifying reasons for presentation and accurately~~

For peer review only - http://bmjopen.bmj.com/site/about/guidelines.xhtml

linking individuals to long-term outcomes.[29] However, longitudinally-linked datasets in other countries could provide insights into long-term outcomes for this vulnerable group of adolescents.

For peer review only

For peer review only - http://bmjopen.bmj.com/site/about/guidelines.xhtml

Contributors

Annie Herbert – helped to conceive and design the study, analysed and interpreted the data, drafted the article, revised it critically for important intellectual content and approved the final version to be published.

Leah Li – helped to conceive and design the study, interpreted the data, revised the article critically for important intellectual content and approved the final version to be published.

Arturo González-Izquierdo – helped to conceive and design the study, acquired and interpreted the data, revised the article critically for important intellectual content and approved the final version to be published.

Ruth Gilbert – helped to conceive and design the study, acquired and interpreted the data, revised the article critically for important intellectual content and approved the final version to be published.

Competing interests None.

Funding AH was supported by the Policy Research Unit in the Health of Children, Young People and Families, which is funded by the Department of Health Policy Research Programme (grant reference number: 109/0001). This report is an independent research commissioned by the Department of Health. The views expressed in this publication are those of the authors and not necessarily those of the Department of Health. AH is also supported by the University College London IMPACT studentship. RG is supported by awards establishing the Farr Institute of Health Informatics Research at University College London Partners from the Medical Research Council and a

For peer review only

For peer review only - http://bmjopen.bmj.com/site/about/guidelines.xhtml

consortium of funders (MR/K006584/1). The study funders played no part in the design, data analysis and interpretation of this study; the writing of the manuscript or the decision to submit the paper for publication, and the authors' work was independent of their funders.

Ethics Both Hospital Episode Statistics (HES) admissions data and Office for National Statistics (ONS) mid-year population estimates are derived from routinely collected administrative data. HES data were pseudonymised before we received them and therefore we did not require Research Ethics Committee approval.

Data sharing No additional data on HES are available. ONS mid-year population estimates may be accessed freely online: http://www.ons.gov.uk/ons/publications/all-releases.html?definition=tcm%3A77-22371.

For peer review only

For peer review only - http://bmjopen.bmj.com/site/about/guidelines.xhtml

REFERENCES

1. Bellis MA, Hughes K, Wood S, et al. National five-year examination of inequalities and trends in emergency hospital admission for violence across England. Injury prevention : journal of the International Society for Child and Adolescent Injury Prevention 2011;**17**(5):319-25 doi: 10.1136/ip.2010.030486[published Online First: Epub Date]|.
2. Hawton K, Rodham K, Evans E, et al. Deliberate self harm in adolescents: self report survey in schools in England. Bmj 2002;**325**(7374):1207-11
3. Meuleners LB, Lee AH, Hendrie D. Interpersonal violence hospitalisations for adolescents: A population-based study. Journal of paediatrics and child health 2010;**46**(11):686-90 doi: 10.1111/j.1440-1754.2010.01818.x[published Online First: Epub Date]|.
4. Lilley R, Owens D, Horrocks J, et al. Hospital care and repetition following self-harm: multicentre comparison of self-poisoning and self-injury. The British Journal of Psychiatry 2008;**192**(6):440-45
5. Snider C, Lee J. Youth violence secondary prevention initiatives in emergency departments: a systematic review. Cjem 2009;**11**(2):161-8
6. Ougrin D, Boege I, Stahl D, et al. Randomised controlled trial of therapeutic assessment versus usual assessment in adolescents with self-harm: 2-year follow-up. Archives of disease in childhood 2013;**98**(10):772-6 doi: 10.1136/archdischild-2012-303200[published Online First: Epub Date]|.
7. Crawford MJ, Patton R, Touquet R, et al. Screening and referral for brief intervention of alcohol-misusing patients in an emergency department: a pragmatic randomised controlled trial. The Lancet;**364**(9442):1334-39 doi: http://dx.doi.org/10.1016/S0140-6736(04)17190-0[published Online First: Epub Date]|.
8. Johnson SB, Bradshaw CP, Wright JL, et al. Characterizing the teachable moment: is an emergency department visit a teachable moment for intervention among assault-injured youth and their parents? Pediatric emergency care 2007;**23**(8):553-59
9. Inpatient HES Data Dictionary. Leeds: Health and Social Care Information Centre, 2010.

Formatted: Font: Cambria, 14 pt, French (France), Do not check spelling or grammar

For peer review only - http://bmjopen.bmj.com/site/about/guidelines.xhtml

10. World Health Organisation. Adolescent Health. Secondary Adolescent Health 2013. http://www.who.int/topics/adolescent_health/en/.
11. National Health Service Act, 2006.
12. Gonzalez-Izquierdo A, Woodman J, Copley L, et al. Variation in recording of child maltreatment in administrative records of hospital admissions for injury in England, 1997-2009. Archives of disease in childhood 2010;**95**(11):918-25 doi: 10.1136/adc.2009.180216[published Online First: Epub Date]|.
13. International Statistical Classification of Diseases and Related Health Problems 10th Revision. Secondary International Statistical Classification of Diseases and Related Health Problems 10th Revision. http://apps.who.int/classifications/icd10/browse/2010/en.
14. Saperia J, Lakhanpaul M, Kemp A, et al. When to suspect child maltreatment: summary of NICE guidance. Bmj 2009;**339**:b2689 doi: 10.1136/bmj.b2689[published Online First: Epub Date]|.
15. Gonzalez-Izquierdo A, Ward A, M OD, et al. Cross-country comparison of victimisation-related injury admission in children and adolescents in England and Western Australia. BMC Health Serv Res 2013;**13**(1):260 doi: 10.1186/1472-6963-13-260[published Online First: Epub Date]|.
16. Patton GC, Viner R. Pubertal transitions in health. Lancet 2007;**369**(9567):1130-9 doi: 10.1016/S0140-6736(07)60366-3[published Online First: Epub Date]|.
17. Gill T. How old are GCSE candidates? Statistics Report Series, 2010.
18. Directgov. AS and A levels. Secondary AS and A levels 2012. http://webarchive.nationalarchives.gov.uk/20121015000000/http://www.direct.gov.uk/en/EducationAndLearning/QualificationsExplained/DG_10039018.
19. Buying Alcohol. Secondary Buying Alcohol 2013. https://www.drinkaware.co.uk/check-the-facts/alcohol-and-the-law/buying-alcohol.
20. All releases of Population Estimates for UK, England and Wales, Scotland and Northern Ireland: Office for National Statistics, 2013.
21. Office for National Statistics. Mid-year population estimates short methods guide: Office for National Statistics, 2010.
22. Radford L. Child abuse and neglect in the UK today. NSPCC Research: NSPCC, 2011.

For peer review only - http://bmjopen.bmj.com/site/about/guidelines.xhtml

23. *Truth hurts: report of the national inquiry into self-harm among young people*. London: Mental Health Foundation, 2006.
24. Hibell B, Guttormsson U, Ahlström S, et al. The 2011 ESPAD Report. Substance use among students in 2012;**36**
25. National Audit Office. Emergency admissions to hospital: managing the demand, 2013.
26. Scott D, Tonmyr L, Fraser J, et al. The utility and challenges of using ICD codes in child maltreatment research: A review of existing literature. Child abuse & neglect 2009;**33**(11):791-808 doi: S0145-2134(09)00202-6 [pii] 10.1016/j.chiabu.2009.08.005[published Online First: Epub Date]|.
27. McKenzie K, Harrison JE, McClure RJ. Identification of alcohol involvement in injury-related hospitalisations using routine data compared to medical record review. Australian and New Zealand journal of public health 2010;**34**(2):146-52 doi: 10.1111/j.1753-6405.2010.00499.x[published Online First: Epub Date]|.
28. Farrar S, Yi D, Sutton M, et al. Has payment by results affected the way that English hospitals provide care? Difference-in-differences analysis. Bmj 2009;**339** doi: 10.1136/bmj.b3047[published Online First: Epub Date]|.
29. Hospital Episode Statistics Accident & Emergency (England), 2007-. Secondary Hospital Episode Statistics Accident & Emergency (England), 2007-. http://adrn.ac.uk/catalogue/cataloguepage?sn=888040.
30. Hawton K, Rodham K, Evans E, et al. Adolescents Who Self Harm: A Comparison of Those Who Go to Hospital and Those Who Do Not. Child Adol Ment H-Uk 2009;**14**(1):24-30 doi: DOI 10.1111/j.1475-3588.2008.00485.x[published Online First: Epub Date]|.
31. What to do if you're worried that a child is being abused. In: Skills DfE, ed.: DfES Publications, 2006.
32. *Self-harm. The short term physical and psychological management and secondary prevention of self-harm in primary and secondary care.* London: National Institute for Health and Clinical Excellence, 2011.
33. *Drug misuse: Psychosocial interventions*. London: National Institute for Health and Clinical Excellence, 2007.
34. Alcohol-use disorder : diagnosis, assessment and management of harmful drinking and alcohol dependence. London: National Institute for Health and Care Excellence, 2011.

Formatted: Font: Cambria, 14 pt, French (France), Do not check spelling or grammar

Formatted: Font: Cambria, 14 pt, French (France), Do not check spelling or grammar

For peer review only - http://bmjopen.bmj.com/site/about/guidelines.xhtml

35. Viner RM, Ozer EM, Denny S, et al. Adolescence and the social determinants of health. Lancet 2012;**379**(9826):1641-52 doi: 10.1016/S0140-6736(12)60149-4[published Online First: Epub Date]|.

For peer review only

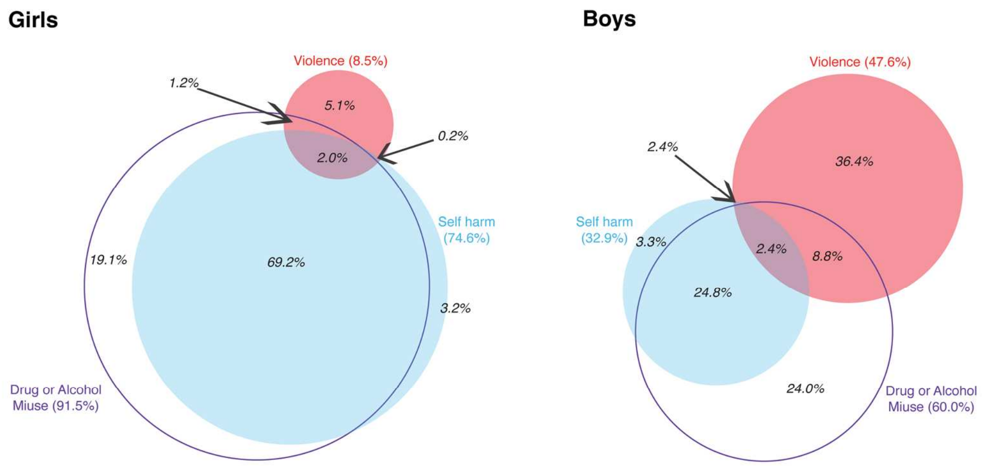

Number (%) of adolescents with adversity-related injury, by types of adversity between 10 and 19 years old* and sex

*Each adolescent classified by all adversity recorded at any emergency admission(s) for injury between10 and 19 years old
173x86mm (300 x 300 DPI)

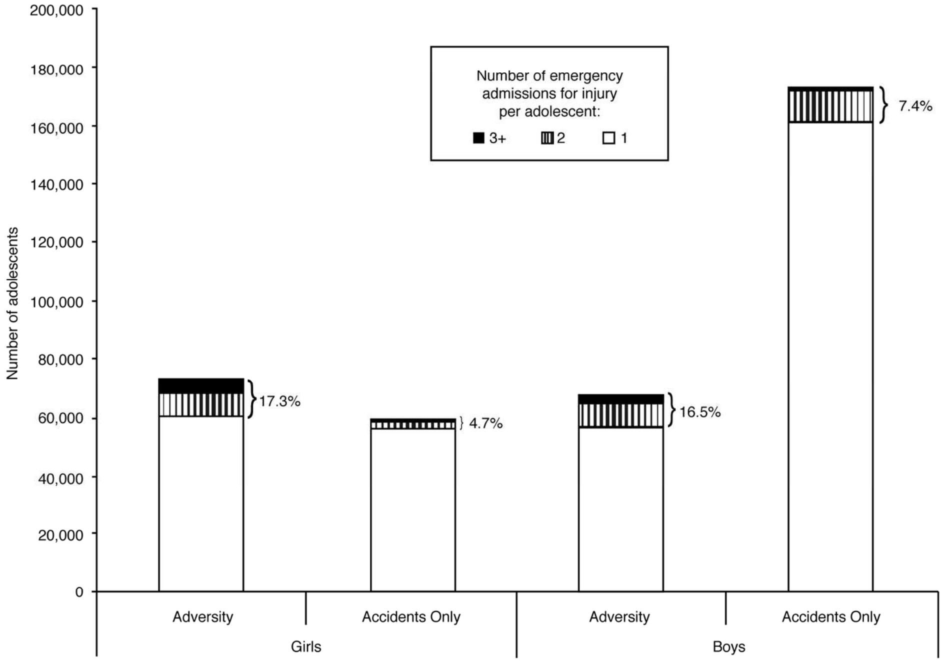

Number of adolescents with 1, 2 and 3 or more emergency admission(s) for injury between 10 and 19 years old*, by types of injury between 10 and 19 years old** and sex

*Percentages are of adolescents who have 2 or more emergency admissions for injury between 10 and 19 years old

**Each adolescent was classified by all adversity/accidents recorded at any emergency admission(s) for injury
173x125mm (300 x 300 DPI)

only

Supplementary Tables

Supplementary Table 1. Cohort (shaded) who have at least one emergency admission for injury between 10 and 19 years old in 1998-2011 (N = 402,916)

1998	*1999*	*2000*	*2001*	*2002*	…	*2007*	*2008*	*2009*	*2010*	*2011*
6	7	8	9	10	…	15	16	17	18	19
7	8	9	10	11	…	16	17	18	19	20
8	9	10	11	12	…	17	18	19	20	21
9	10	11	12	13	…	18	19	20	21	22
10	11	12	13	14	…	19	20	21	22	23

For peer review only

Supplementary Table 2. Definitions and International Classification of Diseases (ICD-10) diagnostic codes used to classify emergency admissions for injury, chronic conditions and types of adversity*

ADMISSIONS AND ADMISSION TYPES

Admission

Admissions were defined by linked episodes of inpatient care, including day cases (any case > 4 hours stay). Any admission which occurred within one day or was a hospital transfer from a previous admission was considered to be the same admission. An episode may have up to 20 diagnosis codes entered, but 88% of admissions in this cohort had episodes with four or less diagnoses recorded (data not shown).

Emergency (acute; unplanned) admission

An admission was considered to be an emergency if the method of admission variable ('admimeth') was 21-24 or 28.(1)

Injury

An admission was considered to be for injury if any of the diagnosis codes in the first episode of the admission were S or T codes (Chapter XIX of the ICD -10).

CHRONIC CONDITIONS

ICD-10 codes as per Hardelid et al, 2013,(2) excluding E24.4, F10-F19, G31.2, G40.5, G62.1, G72.1, K29.2, K70, K85.2, K86.0, O35.4, R78.1-R78.5, Z50.2, Z50.3, Z86.4, X64-X84, Y10-Y34, Z91.5, F30.0-F30.2, F30.8-F32.3, F32.8-F334, F33.8-F34.1, F34.8-F34.9, F38.0-F38.1, F38.8, F39-F40.2, F40.8- F41.3, F41.8-F41.9.

ADVERSITY CLUSTERS AND DESCRIPTIONS*	ICD-10 CODES
**Violence**	
Maltreatment	
Maltreatment syndromes	T74
Effects of other deprivation (extreme neglect)	T73
Perpetrator of neglect and other maltreatment syndromes	Y06, Y07
Assault	
Assault by bodily force and sexual assault	Y04, Y05
Other types of assault	X85 - Y03, Y08 - Y09
Undetermined cause	
Events of undetermined intent	Y20 - Y34
Examination and observation following other inflicted injury	Z04.5
Examination and observation for other reasons: request for expert evidence	Z04.8
**Self-harm**	
Intentional self-poisoning by and exposure to…	
…drugs	X60-X63
…other and unspecified drugs, medicaments and biological substances	X64
…alcohol	X65
…organic solvents and halogenated hydrocarbons and their vapours	X66
…other gases and vapours	X67
…pesticides	X68
…other and unspecified chemicals and noxious	X69

For peer review only

substances	
Intentional self-harm by…	
…hanging, strangulation and suffocation	X70
…drowning and submersion	X71
…firearm discharge	X72-X74
…explosive material	X75
…smoke, fire and flames, or steam, hot vapours and hot objects	X76-X77
…sharp/blunt objects	X78-X79
...jumping from a high place	X80
…jumping or lying before a moving object, or crashing a motor vehicle	X81-82
…other specified means	X83
…unspecified means	X84
Personal history of self-harm	Z91.5
**Drug or alcohol misuse**	
Drugs, medicaments and biological substances (illicit drugs)	
Mental and behavioural disorders due to psychoactive substance use	F11 - F17, F19
Finding of drugs not normally found in blood	R78.1 - R78.5
Poisoning by drugs, medicaments and biological substances	T36 - T50 (not including T50.6)
Poisoning, undetermined intent	Y10 - Y14
Drug rehabilitation	Z50.3
Drug abuse counselling and surveillance	Z71.5
Drug use	Z72.2
Environmental/ Domestic substances	
Mental and behavioural disorders due to use of volatile solvents	F18
Accidental poisoning by and exposure to noxious substances	X40 – X44, X46 - X49
Intentional self-poisoning by and exposure to noxious substances	X69
Poisoning by chemical or noxious substance, undetermined intent	Y16 - Y19
Codes mentioning both alcohol and drugs	
Special epileptic syndromes - (related to alcohol, drugs, etc.)	G40.5
Blood-alcohol and blood-drug test	Z04.0
Alcohol	
Alcohol-induced pseudo-Cushing's syndrome	E24.4
Mental and behavioural disorders due to use of alcohol	F10
Degeneration of nervous system due to alcohol	G31.2
Alcoholic polyneuropathy	G62.1
Alcoholic myopathy	G72.1
Alcoholic cardiomyopathy	I42.1
Alcoholic gastritis	K29.2
Alcoholic liver disease	K70
Alcohol-induced acute pancreatitis	K85.2

For peer review only

Alcohol-induced chronic pancreatitis	K86.0
Maternal care for (suspected) damage to fetus from alcohol	O35.4
Finding of alcohol in blood	R78.0
Poisoning: antidotes and chelating agents, not elsewhere classified	T50.6
Toxic effect of alcohol	T51
Accidental poisoning by exposure to alcohol	X45
Poisoning by exposure to alcohol, undetermined intent	Y15
Evidence of alcohol involvement determined by blood alcohol level	Y90
Evidence of alcohol involvement determined by level of intoxication	Y91
Alcohol rehabilitation	Z50.2
Alcohol abuse counselling and surveillance	Z71.4
Alcohol use	Z72.1
**Accidents**	
Transport accidents	V01 - V99
Falls	W00 - W19
Exposure to inanimate mechanical forces	W20 - W49
Exposure to animate mechanical forces	W50 - W64
Accidental drowning and submersion	W65 - W74
Other accidental threats to breathing	W75 - W84
Exposure to electric current, radiation and extreme ambient air temperature and pressure	W85 - W99
Exposure to smoke, fire and flames	X00 - X09
Contact with heat and hot substances	X10 - X19
Contact with venomous animals and plants	X20 - X29
Exposure to forces of nature	X30 - X39
Accidental poisoning by and exposure to noxious substances	X40 - X49
Overexertion, travel and privation	X50 - X57
Accidental exposure to other and unspecified factors	X58 - X59

For peer review only

Supplementary Table 3. Population prevalence* (per 100,000) of emergency admission(s) for injury, by types of adversity within age periods** and sex

	All girls	All boys	Girls			Boys		
**Adolescent group****	**10-19y**	**10-19y**	**10-14y**	**15-17y**	**18-19y**	**10-14y**	**15-17y**	**18-19y**
All	9,073	16,333‡	4,104	3,260§	2,189§	7,316‡	5,069‡§	3,852‡§
**Adversity**	**4,582**	**4,108‡**	**1,240**	**2,124§**	**1,329§**	**669‡**	**1,458‡§**	**1,748‡§**
Any violence	391	1,970‡	103	152§	110	277‡	731‡§	833‡§
Any self-harm	3,418	1,266‡	777	1,463§	923§	170‡	393‡§	478‡§
Any drug or alcohol misuse	4,194	2,463‡	1,112	1,959§	1,221§	375‡	807‡§	1,077‡§
**Single adversity**	**1,254**	**2616‡**	**495**	**687§**	**412§**	**516**	**991‡§**	**1,114‡§**
Violence only	235	1,496‡	77	99§	75	256‡	609‡§	635‡§
Self-harm only	144	136	48	63§	31§	37‡	39	34
Drug or alcohol misuse only	874	984‡	369	525§	305§	224‡	342‡§	445‡§
**Multiple adversity**	**3,328**	**1,492‡**	**745**	**1,436§**	**917§**	**153‡**	**467‡§**	**634‡§**
V+SH	8	13‡	3	3	1§	1‡	2	2
V+DA	54	361‡	17	37§	26§	19	114‡§	190‡§
SH+DA	3,172	1,018‡	720	1,384§	882§	131‡	345‡§	436‡§
V+SH+DA	93	100‡	6	13§	8	2‡	5‡§	6§
No adversity	4,491	11,417‡	2,676	1,136§	860§	6,648‡	3,611‡	2,171‡§
**Accidents only**	**748**	**10,466‡**	**2,221**	**804§**	**580§**	**5,887‡**	**3,109‡**	**1,784‡§**
**Other causes**	**3,742**	**951‡**	**455**	**332§**	**279§**	**761‡**	**503‡**	**388‡§**

For peer review only

V = Violence, SH = Self-harm, DA = Drug or Alcohol Misuse
*Due to large denominators, confidence intervals for population prevalence were too narrow to provide any meaningful interpretation and so are not shown.
**Each adolescent classified by all adversity/accidents seen at any emergency injury admission(s) at 10-19 years, 10-14 years, 15-17 years and 18-19 years, respectively.
‡Statistically significantly different from girls of the same age (according to 95% confidence intervals).
§Statistically significantly different from 10-14 year olds of the same sex (according to 95% confidence intervals).

For peer review only

REFERENCES

1.Inpatient HES Data Dictionary. Leeds: Health and Social Care Information Centre, 2010.
2.Hardelid P DN, Davey J, Primbramska I, Gilbert R. Overview of child deaths in the four UK countries. London: 2013.

For peer review only

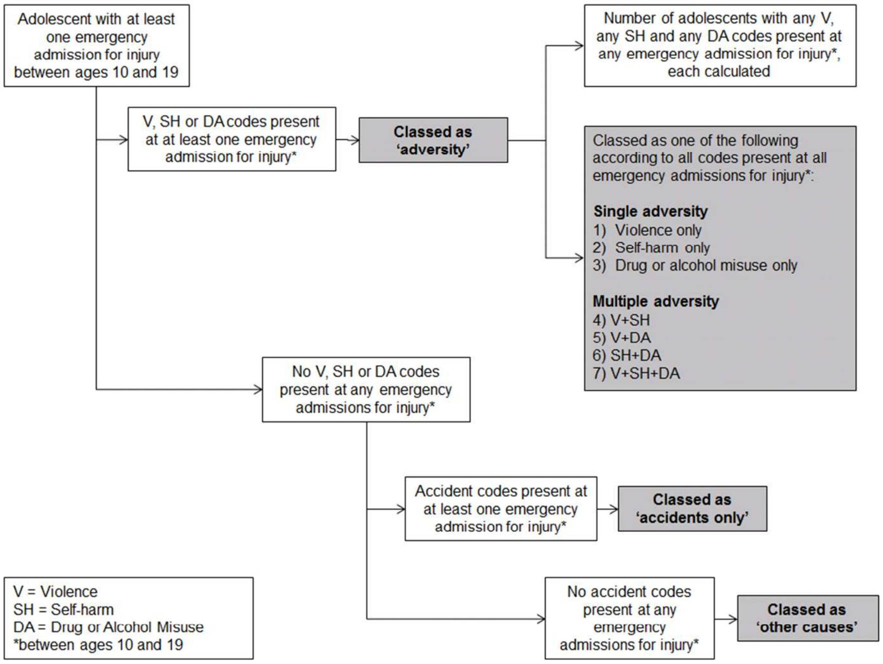

Classification of groups of adolescents.
282x212mm (96 x 96 DPI)

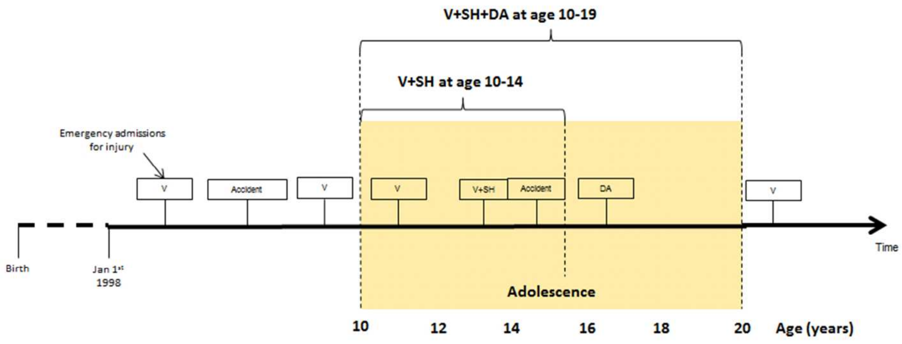

Supplementary Figure 2. Example of classification of groups of adolescents within age groups and across adolescence (ages 10-19).

V = victimisation, SH = self-harm, DA = drug or alcohol misuse.
254x102mm (96 x 96 DPI)

STROBE Statement—checklist of items that should be included in reports of observational studies

	Item No	Recommendation
**Title and abstract**	1	(*a*) Indicate the study's design with a commonly used term in the title or the abstract
		(*b*) Provide in the abstract an informative and balanced summary of what was done and what was found
**Introduction**		
Background/rationale	2	Explain the scientific background and rationale for the investigation being reported
Objectives	3	State specific objectives, including any prespecified hypotheses
**Methods**		
Study design	4	Present key elements of study design early in the paper
Setting	5	Describe the setting, locations, and relevant dates, including periods of recruitment, exposure, follow-up, and data collection
Participants	6	(*a*) *Cohort study*—Give the eligibility criteria, and the sources and methods of selection of participants. Describe methods of follow-up<br>*Case-control study*—Give the eligibility criteria, and the sources and methods of case ascertainment and control selection. Give the rationale for the choice of cases and controls<br>*Cross-sectional study*—Give the eligibility criteria, and the sources and methods of selection of participants
		(*b*) *Cohort study*—For matched studies, give matching criteria and number of exposed and unexposed. N/A<br>*Case-control study*—For matched studies, give matching criteria and the number of controls per case
Variables	7	Clearly define all outcomes, exposures, predictors, potential confounders, and effect modifiers. Give diagnostic criteria, if applicable
Data sources/ measurement	8*	For each variable of interest, give sources of data and details of methods of assessment (measurement). Describe comparability of assessment methods if there is more than one group
Bias	9	Describe any efforts to address potential sources of bias. We only analysed those who could be observed between ages 10 and 19, so no bias due to different lengths of admission trajectories.
Study size	10	Explain how the study size was arrived at. All available administrative data.
Quantitative variables	11	Explain how quantitative variables were handled in the analyses. If applicable, describe which groupings were chosen and why
Statistical methods	12	(*a*) Describe all statistical methods, including those used to control for confounding
		(*b*) Describe any methods used to examine subgroups and interactions
		(*c*) Explain how missing data were addressed. N/A
		(*d*) *Cohort study*—If applicable, explain how loss to follow-up was addressed. We only analysed those who could be observed between ages 10 and 19.<br>*Case-control study*—If applicable, explain how matching of cases and controls was addressed<br>*Cross-sectional study*—If applicable, describe analytical methods taking account of sampling strategy
		(*e*) Describe any sensitivity analyses. N/A

Continued on next page

For peer review only - http://bmjopen.bmj.com/site/about/guidelines.xhtml

**Results**		
Participants	13*	~~(a) Report numbers of individuals at each stage of study—eg numbers potentially eligible, examined for eligibility, confirmed eligible, included in the study, completing follow-up, and analysed~~
		(b) Give reasons for non-participation at each stage. N/A
		(c) Consider use of a flow diagram. We didn't use a flow diagram as eligibility could be described using one sentence.
Descriptive data	14*	~~(a) Give characteristics of study participants (eg demographic, clinical, social) and information on exposures and potential confounders~~
		~~(b) Indicate number of participants with missing data for each variable of interest~~
		(c) *Cohort study*—Summarise follow-up time (eg, average and total amount). N/A
Outcome data	15*	~~*Cohort study*—Report numbers of outcome events or summary measures over time.~~ We state how many emergency admissions for adversity-related injury the adversity group had. We also provide a table indicating the number of emergency admissions for injury and admissions (of any type) during ages 10-19.
		Case-control study—Report numbers in each exposure category, or summary measures of exposure
		Cross-sectional study—Report numbers of outcome events or summary measures
Main results	16	~~(*a*) Give unadjusted estimates and, if applicable, confounder-adjusted estimates and their precision (eg, 95% confidence interval). Make clear which confounders were adjusted for and why they were included.~~ We gave prevalence estimates stratified by age-group and sex.
		~~(*b*) Report category boundaries when continuous variables were categorized~~
		(*c*) If relevant, consider translating estimates of relative risk into absolute risk for a meaningful time period. N/A
Other analyses	17	~~Report other analyses done—eg analyses of subgroups and interactions,~~ and sensitivity analyses
**Discussion**		
Key results	18	~~Summarise key results with reference to study objectives~~
Limitations	19	~~Discuss limitations of the study, taking into account sources of potential bias or imprecision. Discuss both direction and magnitude of any potential bias~~
Interpretation	20	~~Give a cautious overall interpretation of results considering objectives, limitations, multiplicity of analyses, results from similar studies, and other relevant evidence~~
Generalisability	21	~~Discuss the generalisability (external validity) of the study results~~
**Other information**		
Funding	22	~~Give the source of funding and the role of the funders for the present study and, if applicable, for the original study on which the present article is based~~

*Give information separately for cases and controls in case-control studies and, if applicable, for exposed and unexposed groups in cohort and cross-sectional studies.

Note: An Explanation and Elaboration article discusses each checklist item and gives methodological background and published examples of transparent reporting. The STROBE checklist is best used in conjunction with this article (freely available on the Web sites of PLoS Medicine at http://www.plosmedicine.org/, Annals of Internal Medicine at http://www.annals.org/, and Epidemiology at http://www.epidem.com/). Information on the STROBE Initiative is available at www.strobe-statement.org.

For peer review only

For peer review only - http://bmjopen.bmj.com/site/about/guidelines.xhtml
